# Therapeutic effect of mesenchymal stem cells and their derived exosomes in diseases

**DOI:** 10.1186/s43556-025-00277-4

**Published:** 2025-06-04

**Authors:** Yang Wang, Huanhui Wang, Jing Tan, Zhijie Cao, Qun Wang, Hongkun Wang, Shouwei Yue, Wei Li, Dong Wang

**Affiliations:** 1 Department of Rehabilitation, Binzhou Medical University Hospital, Binzhou, China; 2 Binzhou Medical University, Yantai, China; 3 Department of Ultrasound Medicine, Binzhou People’s Hospital, Binzhou, China; 4 Department of Physical Medicine and Rehabilitation, Qilu Hospital, Medical School of Shandong University, Jinan, China; 5 Department of Cardiology, Binzhou Medical University Hospital, Binzhou, China

**Keywords:** Mesenchymal stem cell, Exosome, Cardiovascular diseases, Neurological disorders, Autoimmune diseases, Musculoskeletal disorders

## Abstract

Mesenchymal stem cells (MSCs) are multipotent stem cells characterized by their robust proliferative capacity, homing ability, differentiation potential, and low immunogenicity in vitro. MSCs can be isolated from a variety of tissues, primarily including but not limited to bone marrow, adipose tissue, umbilical cord, placenta, and dental pulp. Although there have been a large number of clinical studies on the treatment of diseases by MSCs and MSCs-derived exosomes (MSCs-EXO), the large-scale clinical application of MSCs and MSCs-EXO have been limited due to the heterogeneity of the results among various studies. This review provides a detailed description of the classification and characterization of MSCs and MSCs-EXO, as well as their extraction methods. Furthermore, this review elaborates on three key mechanisms of MSCs and MSCs-EXO: paracrine mechanisms, immunomodulatory and anti-inflammatory effects, as well as their promotion of tissue regeneration. This review also examines the role of MSCs and MSCs-EXO in cardiovascular diseases, neurological disorders, autoimmune diseases, musculoskeletal disorders, and other systemic diseases over the past five years, while discussing the challenges and difficulties associated with their clinical application. Finally, we systematically summarized and analyzed the potential causes of the various heterogeneous results currently observed. Additionally, we provided an in-depth discussion on the challenges and opportunities associated with the clinical translation of disease treatment approaches based on MSCs, MSCs-EXO, and engineered exosomes.

## Introduction

The concept of stem cell was first proposed in 1868 to describe the properties of fertilized egg [1]. It was identified in murine bone marrow and characterized based on their multilineage differentiation potential [2]. The term of mesenchymal stem cells (MSCs) was formally introduced and separated in 1991 as a distinct category of stem cells, and has been widely utilized in research and clinical applications since then [3]. MSCs are pluripotent stem cells with the capacity of proliferative and self-renewal, homing, pluripotency and low immunogenicity. MSCs come from a wide range of sources. In addition to bone marrow, they can usually be isolated in adipose tissue, dental pulp, umbilical cord and placenta [4].

In 1967, extracellular vesicles (EVs) secreted by chondrocytes was first observed by E Bonucci [5]. EVs are a class of lipid bilayer particles secreted by cells, which are primarily composed of exosomes (50–150 nm) and micro-vesicles (50–1000 nm) [6]. Exosomes, a subtype of EVs, exhibit a topological structure similar to that of cells [7]. In the 1980s, it was established that the normal synthesis and secretion of exosomes are essential for certain physiological processes [8]. Furthermore, subsequent research has indicated that exosome secretion may play a role in the quality control of specific plasma membrane proteins [9]. An increasing body of research has demonstrated that exosomes can mediate intercellular communication via receptor-ligand interactions, participate in a wide range of cellular processes, and play a significant role in the pathogenesis and progression of various diseases [10–12].

In recent years, MSCs and the MSCs-derived exosomes (MSCs-EXO) have demonstrated significant research advances in the treatment of a variety of diseases, such as cardiovascular diseases (CVDs), neurological disorders, autoimmune diseases (ADs), musculoskeletal disorders (MSDs) and some other diseases. This review will focus on the properties and features of different types of MSCs and MSCs-EXO. In addition, we described the mechanism of MSCs and MSCs-EXO in treating diseases. Moreover, we summarize the recent application of MSCs and MSCs-EXO in the clinical treatment of the aforementioned diseases. Finally, we discuss the current status, limitations and future challenges for the application of MSCs and MSCs-EXO in clinical.

## Classification and characterization of MSCs

MSCs, a type of multipotent stromal cells, can adhere to the bottom of the petri dish in cell culture. It can express surface molecules such as CD105, CD73 and CD90, and hardly expresses CD45, CD34, CD14 or CD11b, CD79α or CD19^+^, HLA-DR^+^ [13]. Initially, MSCs were predominantly isolated from bone marrow for applications in disease treatment and tissue repair. However, due to the process of bone marrow-derived MSCs (BM-MSCs) is invasive and painful, researchers have gradually shifted the focus to isolating MSCs from other alternative tissues such as adipose tissue, umbilical cord, and dental pulp. According to their sources, MSCs can be classified into the following categories: BM-MSCs, adipose tissue-derived MSCs (AD-MSCs), umbilical cord-derived MSCs (UC-MSCs), placenta-derived MSCs (P-MSCs), dental pulp-derived MSCs (DP-MSCs), and MSCs from other sources [14]. This section will provide a comprehensive overview of the sources, properties, and characteristics of the above common cell types of MSCs.

### Bone marrow-derived MSCs

**Fig. 1:**
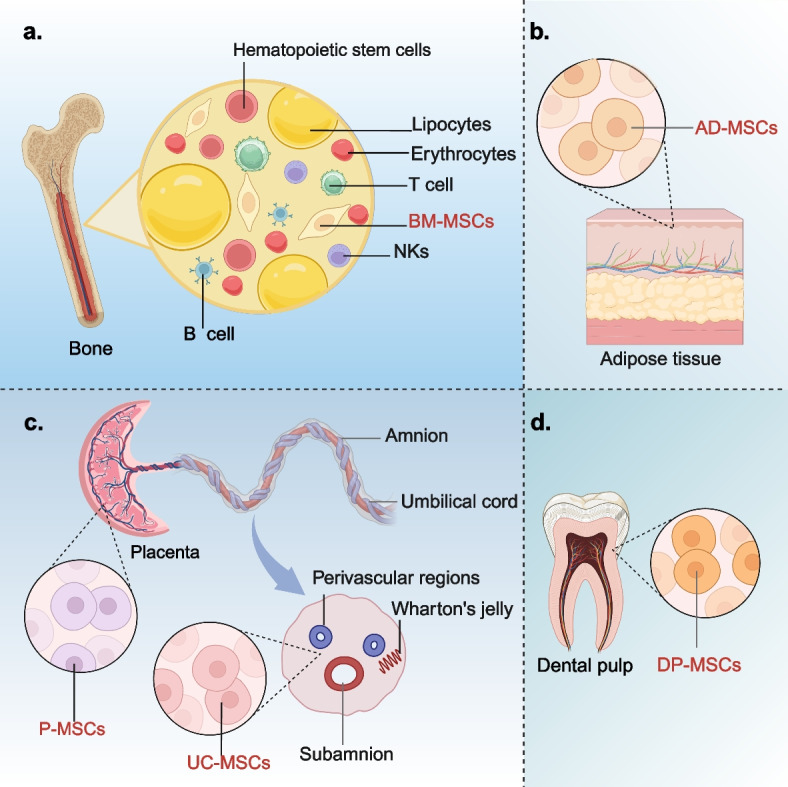
Overview of the classification and characteristics of MSCs. Different types of MSCs are derived from different tissues, mainly from bone marrow (**a**), adipose tissues (**b**), umbilical cord and placenta (**c**), and dental pulp (**d**). MSCs: mesenchymal stem cells; MSCs-EXO: MSCs-derived exosomes; BM-MSCs: bone marrow-derived MSCs; AD-MSCs: adipose tissue-derived MSCs; UC-MSCs: umbilical cord-derived MSCs; P-MSCs: placenta-derived MSCs; DP-MSCs: dental pulp-derived MSCs

In 1976, Friedenstein et al. [15] initially discovered MSCs in the bone marrow of adult mice and named them fibroblasts precursor cells for the first time, also known as BM-MSCs (Fig. 1a). Although the content of BM-MSCs in bone marrow is very small, BM-MSCs are widely used in the treatment of multiple diseases due to their capacity of rapid proliferation, highly stable genetic characteristics, low immunogenicity and excellent multiline differentiation potential [16–18]. The cellular properties of BM-MSCs are usually closely related to the physical health status, pathophysiological status and the age of donor. For example, as donor age increases, indicators of aging in BM-MSCs also rise, which include oxidative damage, the elevated levels of reactive oxygen species (ROS), and the increased expression of p21 and p53 [19]. Consequently, BM-MSCs exhibit enhanced differentiation tendencies. This means that the adaptability and therapeutic effectiveness of BM-MSCs will decline with the increasing of donor ages.

Bone marrow is a complex tissue comprising various cell types. In addition to BM-MSCs, it also contains non-MSCs populations such as hematopoietic stem cells, red blood cells, and various immune-related cells. These diverse cell types collaborate to support hematopoiesis and the immune system. Traditional methods for the isolation and culture of BM-MSCs primarily rely on purification based on intrinsic cellular characteristics, including adhesion time, growth patterns, and tolerance to digestive enzymes during passaging. However, BM-MSCs are not the dominant cell population in the bone marrow. It is hard to develop BM-MSCs into the dominant cell population in the process of isolation and culture. Furthermore, BM-MSCs are frequently co-isolated with other cell types, which poses significant challenges for their purification and subsequent clinical application. In recent years, with the continuous innovation of biological technology, new methods such as density gradient centrifugation, hyaluronic acid hydrogel and flow cytometry have greatly improved the isolation purity of BM-MSCs [20]. These new methods provide a guarantee for further study of the function and clinical application of BM-MSCs. Among the standards established by the International Society for Cell Therapy (ISCT), the three minimal criteria for identifying BM-MSCs are as follows: First, cells must exhibit adherence to plastic substrates. Second, cells must express specific surface antigens, including positivity for CD105, CD73, and CD90, and negativity for CD45, CD34, CD14 or CD11b, and CD79a or CD19. Third, under standardized in vitro culture conditions, these cells must demonstrate the potential to differentiate into osteoblasts, adipocytes, or chondrocytes [21].

It is worth mentioning that the separation method of density gradient centrifugation is usually applicable to the separation of liquid cells and can accurately separate BM-MSCs separated from bone marrow cell suspensions. However, it cannot be applied to tissue matrices characterized by solids such as fat or umbilical cord and placenta [22]. Furthermore, compared with the density gradient centrifugation method, directly isolating BM-MSCs from bone marrow by leveraging their characteristic adhesion to culture plastic is a more advantageous approach. This method can significantly enhance the expression level of HLA-DR, which may serve as a potential marker for evaluating the quality control of MSCs in certain contexts [23]. In addition, during the amplification of BM-MSCs, cell density serves as a critical parameter. Specifically, when the cell density in the ranges of T25 culture flask between 10,000 and 100,000 cells/cm^2^, the expansion rate of MSCs can be significantly enhanced, thereby facilitating large-scale cell culture [24]. After being cultured in vitro for 5 to 7 days, plastic-adherent MSCs may exhibit heterogeneous morphologies resembling fibroblasts, including endothelial cells (ECs), under microscopic observation. During the amplification process, the heterogeneity of adherent MSCs gradually diminishes, with fibroblast-like cells progressively becoming the predominant cell population [21]. The common traditional conditioned medium used for the in vitro culture of BM-MSCs mainly include DMEM/F12, α-MEM and DMEM which supplemented with 10%−15% fetal bovine serum (FBS) [25–27]. However, it has been shown that traditional conditioned medium of BM-MSCs can repair damaged tissues and has the characteristics of high immune compatibility, there are still some safety problems such as allergy in clinical application. In this context, FBS is frequently employed as a supplementary component in traditional culture medium for MSCs. But its use may introduce risks of immunogenicity or contamination. In contrast, human platelet lysate (HPL) has been demonstrated to be a safe and efficient alternative to FBS. HPL effectively mitigates the risks of immune reactions and infections associated with FBS, supports robust MSCs expansion, and holds promise as a viable substitute for FBS in MSCs culture [24]. In recent years, the traditional conditioned medium has been progressively supplanted by emerging serum-free conditioned medium. Compared with the traditional conditioned medium, serum-free conditioned medium can better expand BM-MSCs in vitro, maintain the pluripotency of BM-MSCs, and shorten the culture cycle [28]. This provides a safe guarantee for the clinical application of BM-MSCs.

### Adipose tissue-derived MSCs

AD-MSCs are pluripotent stem cells derived from adipose tissues with the potential to differentiate into various cell lineages (Fig. 1b). More than 90% of the surface antigens on the cell membrane of AD-MSCs are identical with BM-MSCs [29, 30]. Compared to BM-MSCs, AD-MSCs have a broader range of tissue sources. Additionally, the yield of MSCs from adipose tissue is significantly higher than that from bone marrow, and the quantity of AD-MSCs obtained in a single extraction is sufficient to meet the requirements of clinical applications [31]. It can be speculated that the use of freshly isolated cells is likely to be safer compared to cells subjected to in vitro culture and secondary expansion. This is due to the fact that during prolonged in vitro culture, cumulative genetic and epigenetic alterations may occur, potentially affecting cellular function and biological properties [32]. BM-MSCs extraction from bone marrow is a painful process, which often puts stress on the donor. However, the extraction of AD-MSCs from adipose tissue is less psychologically stressful for the donor and more acceptable. Therefore, this also increases the motivation for donors to donate AD-MSCs. Compared with BM-MSCs, AD-MSCs have more stable morphological and genetic characteristics, higher proliferative activity and lower senescence rate under the same in vitro culture conditions [33, 34]. Unlike the isolation of BM-MSCs, the isolation of AD-MSCs necessitates the use of digestive enzymes to separate AD-MSCs from the surrounding fascia, adipocytes, and vascular ECs [35]. Consequently, the process of isolating AD-MSCs demands precise control over the duration of enzymatic digestion to prevent significant adverse effects on cell viability and reparative capacity.

### Umbilical cord-derived and placenta-derived MSCs

In the 1990 s, UC-MSCs were first isolated from connective tissue [36]. The current study shows that UC-MSCs are isolated from four main sites in the umbilical cord: Wharton’s jelly, perivascular regions, subamnion and amnion. Wharton’s jelly region is the main source regions of UC-MSCs (Fig. 1c) [37]. Li et al. compared MSCs from four different sources and found that UC-MSCs from Wharton’s jelly proliferate the fastest which are easy to be isolated and culture, and can maintain the characteristics through multiple generations in vitro [38]. In addition, UC-MSCs express almost no MHC II antigens associated with homoimmune rejection and have undergone barely any cellular ageing [39]. Therefore, compared with adult MSCs, UC-MSCs exhibit lower immunogenicity, non-tumorigenicity, decreased heterogeneity, and enhanced pluripotency. [40]. This means that UC-MSCs have better clinical application potential than adult MSCs. In vitro, The isolation and culture of UC-MSCs can be obtained not only by enzymolysis of collagenase, hyaluronidase or other proteases, but also by explants. UC-MSCs are easy to proliferate and culture, and no ethical concerns about their collection [41, 42]. Compared with UC-MSCs obtained by enzymatic hydrolysis, the number of UC-MSCs obtained from explants was higher due to the significant up-regulation of mitosis and cell cycle related gene expression in acquired cells [43]. These results indicate that enzymatic hydrolysis technology has certain limitations due to its ability to destroy cell surface structures and proteins and reduce cell viability. Explants culture is a milder technique for the isolation and culture of UC-MSCs than enzymatic hydrolysis.

Consistent with the umbilical cord, the placenta also originates from mesenchymal tissue during embryonic development and shares a similar developmental biological background [44]. Therefore, whether it is UC-MSCs or P-MSCs (Fig. 1c), they all have similar immunomodulatory characteristics, low immunogenicity, and consistency in cell morphology [45]. However, owing to the complexity of the placental tissue structure, this typically entails that when preparing P-MSCs, more sophisticated isolation and purification procedures are required to achieve P-MSCs with higher purity [46].

### Dental pulp-derived MSCs

In recent years, DP-MSCs, as an alternative cell source, have demonstrated significant therapeutic potential and have garnered increasing attention from researchers. DP-MSCs are a unique pluripotent cell population with significant mesenchymal characteristics [47]. Different from the origin of BM-MSCs and AD-MSCs, DP-MSCs are mainly derived from the embryonic layer of neural crest and have certain neurophilic characteristics (Fig. 1d) [48]. It is easily isolated and obtained from human third molars removed for orthodontic reasons, and can also be isolated and extracted from the pulp of animals (including dogs) [49]. The abundant cell supply and painless stem cells collection method of DP-MSCs make them a rich source of cells for regenerative medicine with less risk of complications. In addition, DP-MSCs also have advantages such as high proliferation capacity, good differentiation potential, favorable paracrine, minimal invasion, and immune regulation [50]. As a mesenchymal cell population in dental pulp, DP-MSCs have stem cells’ properties expressed by specific markers [51]. In a preclinical study, DP-MSCs were found to express embryonic stem cell markers such as SKIL, MEIS1, and JARID [52]. This suggested that DP-MSCs have the same immunomodulatory and regenerative characteristics as embryonic stem cells. However, there are still other cells in the pulp, such as fibroblasts, highly differentiated dentin-forming odontoblasts, mesenchymal progenitor cells, and perivascular cells. When isolating DP-MSCs, surface markers for positive characterization of MSCs (primarily CD73, CD90, and CD105) are commonly shared with these cells [21]. The detection of a single marker is insufficient to determine the purity of DP-MSCs, and multiparameter flow cytometry addresses this limitation by simultaneously detecting multiple molecular markers [51]. Colony separation is a cell isolation technique that distinguishes the proliferative and self-renewal capacities of stem cells based on cell surface markers, as well as the size and morphology of colonies formed during cell culture. This method is primarily used to isolate DP-MSCs with distinct mesenchymal surface markers; however, it is not suitable for obtaining homogeneous populations of DP-MSCs [53]. Homogeneous populations of DP-MSCs may express similar or identical surface markers, and may have a high degree of morphological and functional consistency during cell culture [54]. When cultured under 3D conditions that more closely mimic the in vivo environment, DP-MSCs can better preserve their original properties, in contrast to the conventional 2D culture environment typically used in standard cell culture dishes [55]. The difference of cell culture density has different effects on the maintenance of stem cell characteristics. The confluent culture condition had a negative effect on the maintenance of stem cell characteristics, while the sparse culture condition had the opposite effect [56]. However, in the densely cultured DP-MSCs, the expression of PI3 K was upregulated [55]. PI3 K catalyzes the intracellular conversion of PIP2 to PIP3. PIP3 recruits AKT to the cell membrane and promotes its activation. Activated AKT can affect the differentiation direction of DP-MSCs and promote their proliferation [57]. Perhaps when DP-MSCs are used for some tissue regeneration, confluent culture may be an alternative strategy.

### Other sources

In recent years, researchers have gradually discovered that MSCs can be successfully isolated from other tissues such as the endometrium [58], peripheral blood [59], ectodermal tissues (such as keratin of the cornea, [60] skin and accessory organs of the eye [61]), skeletal muscle and muscle [59, 62], venous vessels [62], peripheral blood [63] and the fetus including the heart, intestinal epithelium and dermal tissue [64]. However, these tissues do not have a stable source. Ethically, it is also not suitable as a tissue source for large-scale clinical production of MSCs.

## Exosomes and MSCs-derived exosomes

### Characterization and isolation of exosomes

Exosomes are small membrane-bound vesicles of endocytic origin, which can be produced by nearly all organisms and cell types [65]. Specific proteins, lipids, nucleic acids, and glycoconjugates formed the structure of exosomes [66]. In less than five decades since their discovery, exosomes have emerged as a significant research focus due to their advantages such as high safety, easy preservation and transportation, no ethical controversy, and obvious therapeutic effect. Currently, the extraction methods of exosomes mainly include differential ultracentrifugation, ultrafiltration, size-exclusion chromatography, polymer precipitation, microfluidics and immunoaffinity capture. Among these, the gradient ultrafast centrifugation is the most common extraction method [67]. This technique involves the sequential application of increasing centrifugal forces to separate exosomes from other cellular components based on their buoyant densities. The process typically involves multiple rounds of centrifugation, yielding purified exosomes in the final pellet [68]. However, this method comes with several drawbacks, including the high cost of equipment, the time-consuming nature of the process, and the instability in the number of exosomes obtained. In recent years, the method of polymerization-induced precipitation has gained considerable popularity among researchers due to its cost-effectiveness and high yield, and has emerged as a viable alternative, gradually becoming the second choice for exosome extraction, as evident in various studies [69]. The principle of polymerization-induced precipitation is similar to Ethanol-mediated nucleic acid precipitation which forms a hydrophobic microenvironment through the interaction of highly hydrophilic polymers with water molecules around exosomes. This causes the exosomes to precipitate [70]. However, compared with differential ultracentrifugation, this method can lead to non-specific protein contamination and may influence the analysis of downstream results [71]. For exosomes that have been subjected to extraction and purification, a comprehensive quality assessment is typically performed from the perspectives of particle size distribution, quantification, and biological activity. When performing quality assessment, a range of advanced techniques can be employed, such as nanoparticle tracking analysis (NTA) [72], transmission electron microscopy (TEM) [73], atomic force microscopy (AFM) [74], and Western blotting [74]. These techniques not only enable precise evaluation of the size distribution, morphological characteristics, purity level, and protein composition of exosomes but also ensure that the extracted exosomes comply with the anticipated quality and purity standards. Moreover, exosomes demonstrate remarkable resilience to various storage conditions. They can be preserved under freezing conditions or via freeze-drying technology, which substantially decreases costs associated with manufacturing, storage, and transportation [74]. Therefore, selecting an appropriate exosome extraction method, as well as quality control and storage approaches, is of particular importance for ensuring the accuracy of subsequent experimental results.

### Roles of exosomes in cell communication

The production process of exosomes primarily involves several key steps: the formation of the plasma membrane, the sorting of endosomes and multivesicular bodies, and the release of exosomes through the fusion of multivesicular bodies with the plasma membrane [75]. During the biogenesis of exosomes, various substances, including nucleic acids (miRNA, long non-coding RNA, circRNA, and DNA), proteins, lipids, and even mitochondria, are encapsulated and subsequently delivered to target cells [76]. MiRNAs, as one of the key molecular cargos delivered by exosomes, are initially transcribed into primary miRNAs in the nucleus. These primary miRNAs are subsequently processed by the Drosha enzyme to generate precursor miRNAs, which are then transported to the cytoplasm via Exportin-5. In the cytoplasm, these precursors are further processed into mature miRNAs. During exosome biogenesis, mature miRNAs can associate with RNA-induced silencing complexes (RISCs) and subsequently be encapsulated within exosomes, thereby becoming functional components of these EVs [77]. MiRNAs not only protect exosomes from enzymatic degradation during delivery but also facilitate their transfer to target cells via endocytosis or by binding to specific receptors. Additionally, miRNAs can bind to the 3'UTR region of target-cell mRNA, thereby inhibiting translation or inducing degradation and consequently modulating the expression of downstream genes [78].

Mitochondria, as important organelles within cells, can regulate energy metabolism, apoptosis and cell signal transduction. The transcellular transfer of mitochondria is the key mechanism to ensure the smooth execution of its functions. Exosomes, as one of the microtubule cytoskeletons with common intracellular movement patterns, can provide significant impetus for the transport complexes on the cytoskeleton to drive mitochondrial movement [79]. Under the stimulation of energy stimulation, oxidative stress or DNA damage, the intracellular ROS level increases, and exosomes can achieve mitochondrial transcellular transfer. Research has found that astrocytes can release a large number of exosomes containing mitochondria under the conditions of stimulating ischemic stroke in vivo and excreting serum in vitro, reducing peripheral nerve damage [80]. In a mouse model of lipopolysaccharide-induced lung injury, exosome-mediated functional mitochondrial transfer can promote anti-inflammatory and antioxidant stress in mouse macrophages [81]. As a transmembrane protein, the NAD +/CD38/cADPR/Ca^2+^ pathway mediated by CD38 is the key to exosome-mediated mitochondrial transcellular transport. This pathway can not only be involved in the endocytosis stage of exosomes, but also in the exocytosis stage, influencing intercellular communication and material exchange [82]. The inhibition of endocytosis will lead to a reduction in mitochondrial transfer from BM-MSCs to damaged alveolar epithelial cells. Furthermore, Connexin 43 (CX43), as a connexin of mitochondria, the interstitial junction channels it mediates can significantly enhance the efficiency of mitochondrial transcellular transfer. Under certain conditions, it can protect myocardial and brain tissues from ischemia–reperfusion injury and is involved in the endocytosis of reticin-dependent exosomes [83].

Exosomes are secreted from parent cells and their targeting mechanisms remain incompletely understood. Research has demonstrated that exosomes can be internalized by recipient cells through protein–protein interactions (such as fusion with the plasma membrane and direct interaction with surface receptors) or receptor-ligand interactions (including caveolae-dependent or caveolae-independent endocytosis), thereby performing their functional roles [84]. In the first way, integrins, tetraspanins, and other adhesion proteins play a crucial role in exosome targeting and the selective uptake by recipient cells [85]. Integrins are cell adhesion molecules that play an indispensable role in regulating cell growth and function. Targeting integrins α6β4 and αvβ5 can reduce the uptake of exosomes by lung-tropic and liver-tropic tumor cells, thereby decreasing metastasis to the lung and liver, respectively [86]. Connexins are another class of tetraspanins that facilitate intercellular communication on exosome membranes, in addition to integrins. Connexins are primarily composed of two extracellular loops and one cytoplasmic loop, enabling their biological functions through connexin-integrin cross-talk. Notably, CX43, a key member of the connexin family, plays a significant role in regulating exosome release and molecular transfer [87, 88]. CD81, CD9, and CD63 were initially identified as tetraspanin markers of exosomes. However, subsequent research revealed that only CD63 is a specific protein marker for exosomes, whereas CD9 and CD81 are not [89]. Moreover, recent studies have shown that in certain cell types, the absence of these three proteins has minimal impact on the protein composition of exosomes [90]. Previous studies have demonstrated that inhibiting endocytosis in recipient cells can reduce their uptake of exosomes. However, the relationship between endocytosis and exosome release within the same cell remains undetermined. Recent research indicates that endocytosis can inhibit the secretion of vesicular exosome marker proteins (CD81, CD9, and CD63). Additionally, endocytosis can trigger the degradation of exosomes containing CD9 and CD81 [91]. This suggests that cells can either primarily release exosomes as parent cells or simultaneously receive exosomes. This finding provides valuable insights for future research on the production and modification of post-therapeutic exosomes. Exosomes, as secretory vesicles, can be utilized by adjacent cells to exert their functions. For instance, after entering the bloodstream, exosomes can be transported over long distances to reach lesion sites and deliver therapeutic effects. However, hepatic accumulation of exosomes can diminish their therapeutic efficacy [92]. Therefore, engineering exosomes holds significant potential for enhancing their therapeutic performance. Currently, researchers have demonstrated that blocking Scavenger Receptor Class A (a novel monocyte/macrophage uptake receptor for EVs) with dextran sulfate significantly reduces the clearance of EVs in the liver [93]. This finding is crucial for enhancing the effective utilization of exosomes.

### MSCs-derived exosomes

**Fig. 2:**
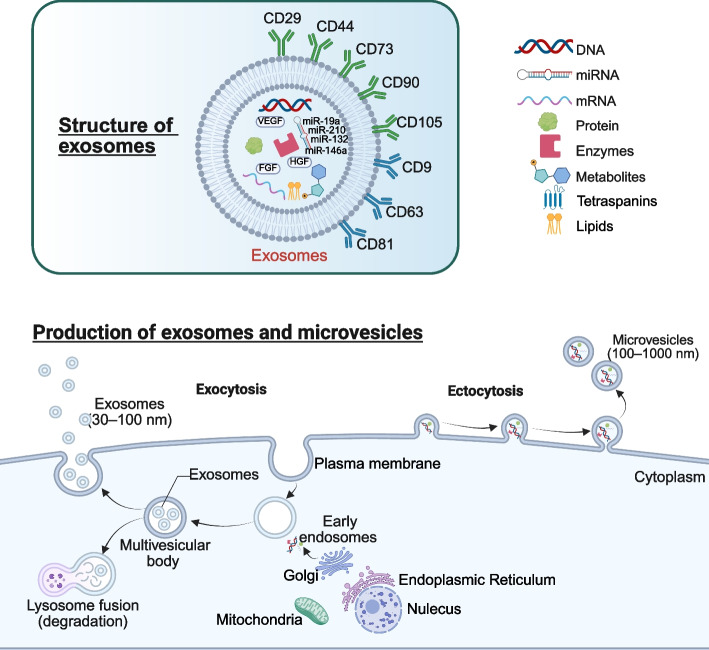
Overview of the characteristics and formation process of exosomes. The formation process of exosomes mainly includes: the formation of plasma membrane, the sorting endosomes and multivesicular body and the release of exosomes by the combination of multivesicular body and plasma membrane

MSCs can secrete a lot of secretomes, including cytokines, growth factors and chemokines, and extracellular nanoparticles dominated by exosomes [48]. Compared to the challenges of iatrogenic tumorigenesis, immune rejection, vascular blockage, and ethical concerns faced by MSCs in clinical applications, MSCs-EXO not only effectively mitigate these issues but also exhibit comparable reparative effects to those of MSCs [94]. MSCs-EXO carry not only the characteristic markers of MSCs (such as CD29, CD44, CD73, CD90, and CD105), but also the characteristic markers of exosomes (such as CD9, CD63, and CD81) [95]. MSCs-EXO primarily exerts its functions by transferring various bioactive molecules, including growth factors (such as VEGF, FGF, HGF), anti-inflammatory proteins, anti-apoptotic proteins, cell survival-promoting factors, angiogenic proteins, microRNAs (such as miR-19a, miR-132, miR-146a, miR-210), long non-coding RNAs, circRNAs, mRNAs, and lipids (Fig. 2) [76, 96–104]. This is usually achieved by the target cell internalizing MSCs-EXO into the cell through endocytosis. After the proteomics analysis of AD-MSCs-derived exosomes (AD-MSCs-EXO), BM-MSCs-derived exosomes (BM-MSCs-EXO), UC-MSCs-derived exosomes (UC-MSCs-EXO) and DP-MSCs-derived exosomes (DP-MSCs-EXO). Zheng-Gang Wang et al. found 771, 457 and 431 proteins respectively, 355 of which were common proteins [105]. This study indirectly suggests that different MSCs-EXO may have different therapeutic effects on the same disease. In addition, miRNA is also an important component of MSCs-EXO and plays an important role in the treatment of various diseases [106–108]. The types and amounts of proteins and miRNAs in MSCs change with the aging of MSCs [109, 110]. Therefore, it can be speculated that the contents of MSCs-EXO will also change with the aging of MSCs, and the cell viability of MSCs will directly affect the therapeutic effect of MSCs-EXO.

## Mechanisms Underlying the Therapeutic Effects of MSCs and MSCs-EXO

### Paracrine Signaling

Paracrine pathway is considered to be the primary pathway for stem cell therapy. In a narrow sense, paracrine factors with diameters less than 10 nm secreted by stem cells are referred to as paracrine substances, and the term paracrine encompasses all secretory products, including exosomes and other cellular vesicles in a broader context [111]. Extensive research has demonstrated that stem cells secrete a wide array of bioactive molecules, such as anti-inflammatory factors, growth factors, cytokines, and specific active factors [112, 113]. These secretomes play crucial roles in regulating stem cell homing, enhancing self-survival, promoting anti-apoptotic effects, stimulating angiogenesis, modulating immune responses, inhibiting excessive fibrosis, and facilitating cell proliferation, survival, and differentiation [114, 115].

The conditioned medium from stem cells demonstrates a therapeutic efficacy comparable to that of stem cells themselves, providing robust evidence that the paracrine mechanism plays a crucial role in the disease-treating effects of stem cells. For instance, the conditioned medium from DP-MSCs not only alleviates cardiomyocyte apoptosis in ischemia–reperfusion (I/R) mouse models but also inhibits I/R-induced cardiomyocyte apoptosis in vitro [116]. For AD-MSCs, their conditioned medium could attenuate I/R-induced cardiac injury through the microRNA-221/222/PUMA/ETS-1 pathway [117]. In addition, conditioned medium from AD-MSCs could also protect neuronal and oligodendroglia cells exposed to oxygen–glucose deprivation (OGD) [118]. It is well established that external stimuli enhance the paracrine activity of MSCs, offering valuable insights for leveraging this property in disease treatment. Therefore, hypoxia [119], ischemia [120], carbon monoxide exposure [121], physical factors [122], beneficial chemical elements [123], cytokines [124], and pharmacological agents [125] can be employed to enhance the paracrine effect and efficacy of MSCs. For example, under ischemic conditions, MSCs can mitigate cardiomyocyte apoptosis by secreting exosomes enriched with miR-22 [126, 127]. Compared with UC-MSCs in normal oxygen environment, hypoxia-preconditioned UC-MSCs have better angiogenesis in the treatment of myocardial infarction (MI) mice. Furthermore, the latter conditioned medium can better promote the formation of human umbilical vein EC tubules in vitro [128]. In the osteochondral defect model, hypoxia-preconditioned BM-MSCs facilitate cartilage regeneration and attenuate joint inflammation through the mediation of EVs [129]. MSCs pretreated with metformin can enhance their proliferative capacity and activity by augmenting their paracrine effects, thereby mitigating inflammation and fibrosis in chronic kidney disease (CKD) models [130].

The combination of MSCs with gene editing technology, or the integration of MSCs with other therapeutic modalities, represents another important strategy for enhancing paracrine activity. For example, the genes associated with hypoxia resistance and enhanced migration, including HIF-1α [131], SDF-1 [132], HO-1 [133], CXCR4 [134], and specific miRNAs [135–137], were transferred into MSCs through gene editing technology could improve the repair ability of MSCs. The combination of pharmacological agents, physical factors, and novel polymeric materials with MSCs can improve therapeutic efficacy and augment immune modulation [138–142]. Although the synergistic mechanisms underlying combination therapy have not been fully elucidated, several studies suggest that they may be associated with enhanced paracrine effects of MSCs [143, 144]. Therefore, actively elucidating the mechanisms by which combination therapy achieves a synergistic effect on paracrine signaling will be a key focus of future research.

### Immunomodulatory and anti-inflammatory

Innate immunity is an important part of immune response. Cells involved in innate immunity primarily include neutrophils, monocytes, macrophages, and dendritic cells (DCs), as well as specific lymphocyte populations such as natural killer (NK) cells and γδ T cells. These cells secrete inflammatory mediators like ROS, IL-6, and TNF-α in response to various exogenous and endogenous stimuli, thereby inducing an inflammatory response [145].

Within the first few hours following tissue damage, innate immune responses will gradually be initiated. During this process, in addition to growth factors, chemokines, and pro-fibrotic factors, the damaged tissues can also release a large number of inflammatory factors. The change of microenvironment and the accumulation of inflammatory factors can lead to the increase of vascular permeability and drive inflammatory cells to gather at the damaged area [146]. Various pro-inflammatory cytokines and chemokines released by activated macrophages co-create a pro-inflammatory environment with cytokines released by damaged tissue. This pro-inflammatory environment attracts neutrophils to the damaged area to participate in early inflammatory responses. This process usually occurs within 6 h after tissue damage [147]. Adaptive immunity, primarily mediated by B and T lymphocytes, enables the recognition of specific pathogens and the generation of pathogen-specific antibodies. For example, in the treatment of malignant tumor diseases, MSCs can stimulate central granulocytes to secrete IL-10 and PGE2, thereby inhibiting the proliferation of T lymphocytes in the tumor microenvironment [148]. This immune response plays a crucial regulatory role in the pathogenesis and progression of diseases such as atherosclerosis, systemic lupus erythematosus (SLE), cancer and osteoarthritis (OA) [148–152].

The immune response can both inflict damage on tissues and trigger the wound healing process, including the formation of scar tissue [153]. Therefore, there exists a delicate balance between the damaging and reparative effects during the inflammatory response. If this balance is disrupted, it can lead to either an excessive inflammatory response or inadequate repair of tissue damage. Consequently, maintaining the equilibrium between pro-inflammatory and anti-inflammatory responses is essential for optimizing the protective effect on injured cells. For example, on the first day after myocardial damage, the activated M1 macrophages and their secreted cytokines, together with attracted neutrophils, create a more intense pro-inflammatory environment. More immune cells are attracted and additional damage is caused to cardiomyocytes [147]. However, macrophages can also change from M1 type to M2 type, and the cytokines secreted by M2 type macrophages are mainly anti-inflammatory, wound healing and angiogenesis [154]. This is crucial for the protection and preservation of damaged cardiomyocytes. In recent years, a substantial body of research has elucidated the significant role of MSCs and MSCs-EXO in immunomodulatory and anti-inflammatory across various diseases, including MSDs, CVDs, neurological disorders, ADs, and others.

**Fig. 3:**
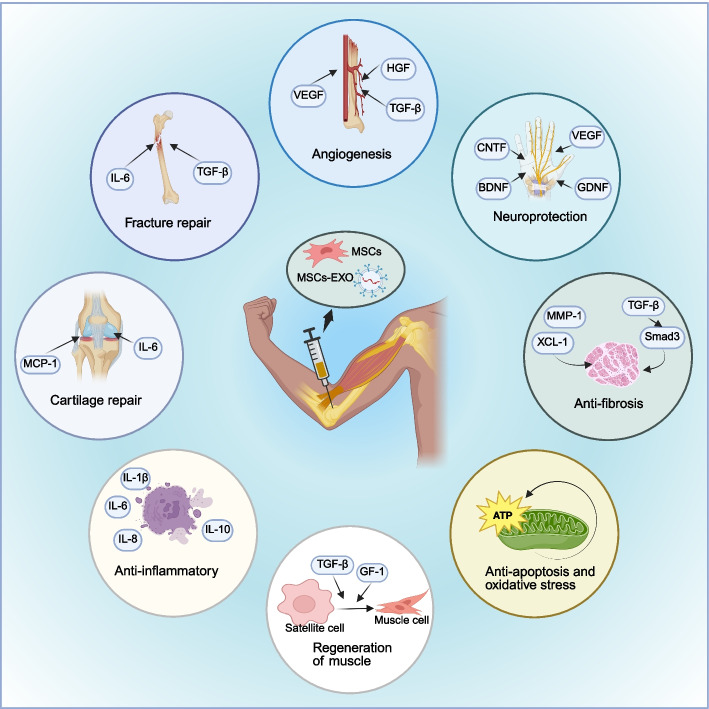
Application of MSCs and MSCs-EXO in MSDs. MSCs and MSCs-EXO are primarily administered via intra-articular injection to the affected site, exerting therapeutic effects such as fracture repair, cartilage regeneration, anti-inflammatory responses, muscle regeneration, anti-apoptosis, oxidative stress reduction, anti-fibrosis, neuroprotection, and angiogenesis for the treatment of musculoskeletal diseases. MSCs: mesenchymal stem cells; MSCs-EXO: MSCs-derived exosomes; MSDs: musculoskeletal disorders

OA is a representative disease of MSDs. It is a complex, multifactorial degenerative condition that arises from the interplay of biomechanical factors and inflammatory processes. MSCs and MSCs-EXO can attenuate the activity of pro-inflammatory immune cells and modulate local and systemic biomarkers, thereby regulating the immune response in OA (Fig. 3) [155]. Mitochondria are key organelles in oxidative stress. Mitochondria derived from MSCs can reverse abnormalities in energy metabolism and mitochondrial dynamics in chondrocytes in OA, thereby enhancing chondrocytes'resistance to oxidative stress and apoptosis [156]. In addition, MSCs-EXO can alleviate cartilage degradation and synovial inflammation in OA through ferroptosis [157]. Furthermore, the synergistic integration of biological scaffolds composed of polymer materials with MSCs can augment their anti-inflammatory effects in the OA microenvironment [158, 159].

**Fig. 4:**
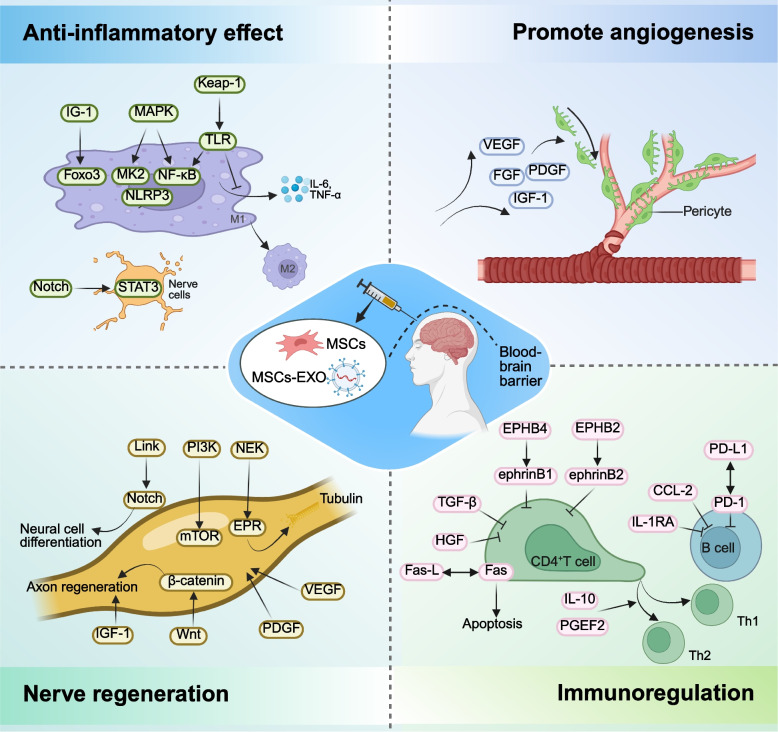
Application of MSCs and MSCs-EXO in neurological disorders. MSCs and MSCs-EXO can exert therapeutic effects by reducing inflammation, promoting angiogenesis, facilitating nerve regeneration, and modulating immune responses. MSCs: mesenchymal stem cells; MSCs-EXO: MSCs-derived exosomes

Neurological disorders are frequently associated with neuroinflammation (Fig. 4). Neuroinflammation is activated by damage-associated molecular patterns (DAMPs) or pathogen-associated molecular patterns (PAMPs) [160]. Upon damage to neurons and glial cells, the DAMPs secreted by these cells can activate microglia and astrocytes, leading to changes in their morphology and function [161]. Microglia and astrocytes serve as the primary effector cells of the innate immune response in the central nervous system. Their activation subsequently enables these cells to exert their immunomodulatory effects. Similar to macrophages, activated microglia exhibit two distinct phenotypes: M1 and M2. The M1 phenotype is primarily associated with pro-inflammatory responses, whereas the M2 phenotype is predominantly involved in anti-inflammatory and neuroprotective functions [162]. Astrocytes can also produce pro-inflammatory or immunomodulatory mediators depending on their polarization phenotype [163]. MSCs represent an ideal cell source for neural reparation and inflammation reduction due to their low immunogenicity and paracrine effects. MSCs primarily modulate neuroinflammation through their interactions with microglia and astrocytes. For example, MSCs-EXO can inhibit inflammation and promote the recovery of neurological function by entering microglia and suppressing their activation [164]. Furthermore, MSCs-EXO enriched in miR-216a-5p can also regulate microglial polarization by modulating the TLR4/NF-κB/PI3 K/AKT signaling pathway [165]. GFAP and IBA1 serve as markers for astrocytes and microglia, respectively. Exosome therapy can significantly reduce the transcription levels of inflammatory markers in astrocytes and microglia, indicating a diminished inflammatory state following spinal cord injury (SCI) [166]. Post-stroke inflammatory injury is a critical factor that exacerbates stroke-induced brain damage. MSCs overexpressing HO-1 exhibit a significant therapeutic effect on hyperinflammatory injury following stroke in mice, ultimately promoting recovery after ischemic stroke [167].

**Fig. 5:**
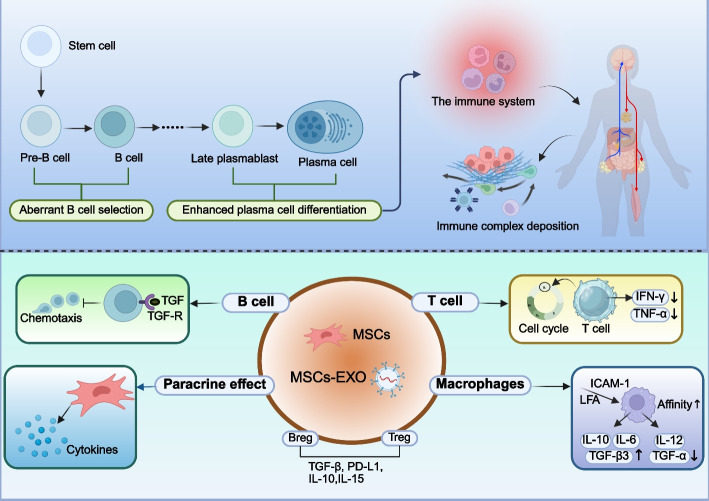
Application of MSCs and MSCs-EXO in ADs. ADs primarily arise from aberrant B cell selection and enhanced plasma cell differentiation, leading to the immune system attacking normal human tissues. This results in the deposition of immune complexes. MSCs can inhibit the proliferation of B cells, T cells, and macrophages, promote the release of anti-inflammatory factors, and reduce the production of pro-inflammatory factors. Furthermore, MSCs facilitate the differentiation of T cells and B cells into regulatory subsets, such as Tregs and Bregs. ADs: autoimmune disease; MSCs: mesenchymal stem cells; MSCs-EXO: MSCs-derived exosomes; Treg: regulatory T cells; Breg: regulatory B cells

ADs comprise a group of chronic inflammatory disorders characterized by the immune system attacking the body's own tissues, leading to the formation and deposition of immune complexes [168]. MSCs and MSCs-EXO can elicit either inhibitory or stimulatory immune responses, thereby participating in immune regulation and modulation. The aberrant expression of autoantibodies is a key mechanism underlying the occurrence and development of AD, and the emergence of these autoantibodies is frequently attributed to aberrant B cell selection or enhanced plasma cell differentiation, indicating a breakdown in self-tolerance (Fig. 5) [169]. MSCs and MSCs-EXO contribute to the treatment of AD by modulating the aberrant expression of autoantibodies [170]. BM-MSCs can inhibit antigen-dependent proliferation and differentiation into plasma cells of follicular and marginal zone B cells in SLE mice via the IFN-γ and PD-1/PD-L1 pathways, thereby contributing to the prevention of glomerular damage [171].

CD4 + T cells also play a crucial role in the pathogenesis of multiple AD, contributing to target organ damage through the production of cytokines such as IFN-γ, TNF-α, IL-4, IL-17, IL-21, and IL-23 [171, 172]. For example, MSCs can promote the expression of IL-10, downregulate the expression of IL-18 and Th17 cells, thereby correcting the Th17/Treg (a type of CD4 + T) imbalance to exert immunomodulatory effects [173]. Currently, glucocorticoids serve as the cornerstone therapy for the treatment of most AD. Dexamethasone-integrated MSCs and MSCs-EXO can treat SLE by inhibiting the release of IFN-γ and TNF-α from CD4 + T cells and upregulating the expression of CRISPLD2 [174]. Moreover, Dexamethasone-integrated MSC-based biomimetic liposomes enhance their affinity for polarized macrophages via the LFA-1/ICAM-1 interaction, thereby improving the therapeutic efficacy in rheumatoid arthritis (RA) [175].

In ischemic heart disease, MSCs can reduce the production of inflammatory cytokines, including TNF-α, IL-1β, and IL-6. This finding further demonstrates the potential therapeutic value of MSCs in cardiac repair [176]. In addition, MSCs-EXO carried by specific miRNA may play a regulatory role in the development of inflammation after MI [177, 178]. These anti-inflammatory effects are mainly achieved through the paracrine mechanisms of MSCs and MSCs-EXO [179]. For example, AD-MSCs-EXO carrying miR-93-5p may prevent myocardial damage by inhibiting TLR4-mediated inflammatory responses [180]. Zilun et al. showed that MSCs-EXO carrying miR-181a inhibited c-FOS protein through cell targeting properties and the immunosuppressive effect of miR-81a, thus inhibiting inflammatory response [181]. Neutrophils are inflammatory cells that accumulate in the border area early after MI, so it is essential to investigate the relationship between MSCs and them. Although some studies have shown that BM-MSCs activated by TLR3 and TLR4 in vitro can reduce neutrophil apoptosis through the combined action of IL-6, IL-β and granulocyte macrophage colony-stimulating factor [182]. This does not fully explain why MSCs can inhibit the inflammatory response after myocardial damage. At present, the mainstream view is that MSCs can play a role by inhibiting the activity of neutrophils and accelerating their apoptosis. For example, Dongsheng Jiang et al. suggested that MSCs could phagocytic ICAM1-dependent neutrophils, resulting in a decrease in the number of neutrophils [183]. Other studies have shown that MSCs-EXO can inhibit the excessive spread of inflammation by inhibiting the overactivity of neutrophils [184]. Moreover, the conditioned medium of MSCs have also been found to induce neutrophil apoptosis in vitro [185]. Macrophages are one of the important immune cells that regulate the occurrence and development of inflammation after myocardial damage. Studies have shown that MSCs can accelerate the transformation of M1 macrophages into M2 macrophages in infarct myocardium, it can achieve the purpose of modulating the immunologic environment, accelerating regeneration and inhibiting inflammation [186]. In addition, specifically genetically modified or upregulated MSCs and MSCs-EXO can the duration of inflammation and reduce cardiomyocyte damage by promoting transformation between macrophage subtypes [187, 188]. In summary, it is of great significance to explore how MSCs and MSCs-EXO promote the transformation of M1 macrophages to M2 macrophages and further find the balance between anti-inflammatory and pro- inflammatory.

### Promotion of Tissue Regeneration

**Fig. 6:**
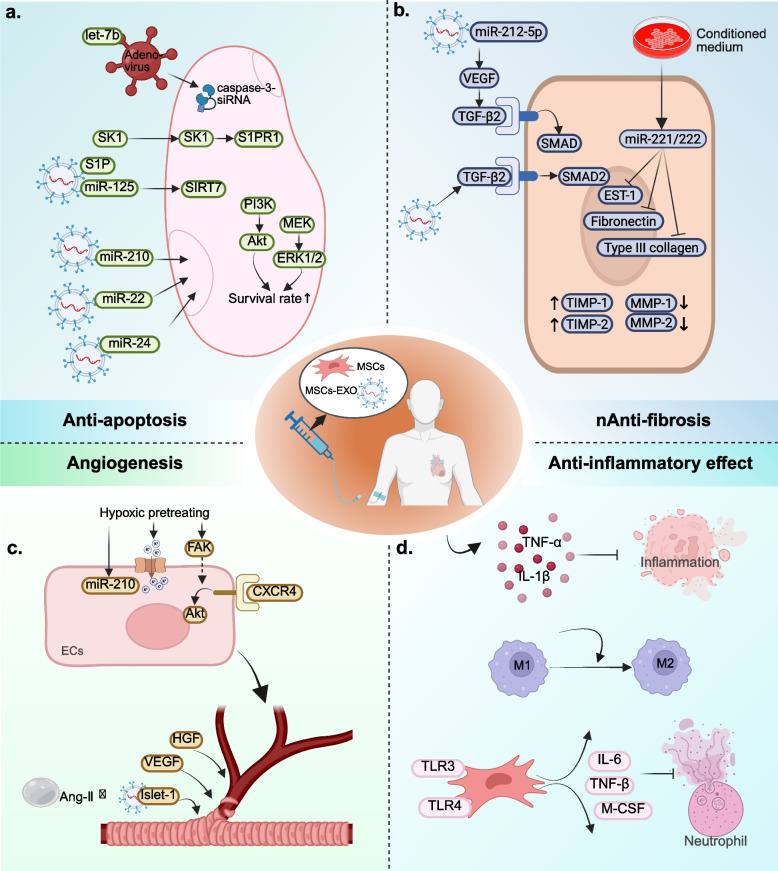
Application of MSCs and MSCs-EXO in MI. **a** Overview of anti-apoptotic of MSCs and MSCs-EXO; **b** Overview of anti-fibrosis of MSCs and MSCs-EXO; **c** Overview of angiogenesis of MSCs and MSCs-EXO; **d** Overview of anti-inflammatory effect of MSCs and MSCs-EXO. MSCs: mesenchymal stem cells; MSCs-EXO: MSCs-derived exosomes; MI: myocardial infarction

Inhibition of apoptosis, promotion of angiogenesis, targeted differentiation into damaged tissues, and suppression of excessive inflammation constitute four key mechanisms that facilitate tissue regeneration (Fig. 6).

Apoptosis is a complex biological process, and the majority of the mechanisms underlying apoptosis involve a specific type of intracellular enzyme known as the cysteine aspartic acid specific protease (caspase) [189]. The Caspase cascade system plays a crucial role in the induction, transduction and amplification of apoptosis signals within cells. Its activation and function are tightly regulated by a variety of protein molecules and ions including apoptosis inhibitor proteins, Bcl-2 family proteins, calpain, and Ca^2+^ [190]. At least 7 of the 14 known caspases proteins play a direct role in regulating apoptosis in mammals, while others can also indirectly influence cell death through inflammatory response [191]. Using MI as an example, previous studies have shown that the application of non-selective caspase inhibitors can protect cardiomyocytes from fatal I/R damage in the early stage of MI [192]. Of these known proteins, the activation of caspase-3 is the most crucial in regulating apoptosis following MI. The activation of caspase-3 and the increase of cardiac natriuretic peptides in serum can be detected in non-infarcted areas 1 day after MI and can sustain for up to 4 weeks. At the same time, the Bcl-2/Bax ratio shifted in the direction of pro-apoptotic Bax direction [193]. Chandrashekhar et al. also obtained similar research results through experiments [194]. This suggested that heart failure (HF) may occur in the early stage of MI. Using caspase-3-siRNA to intervene in caspase-3 expression in acute MI (AMI) mice can significantly reduce infarct size and decrease the apoptotic index. Additionally, it improves ventricular function in AMI mice [195]. MSCs transduced with adenovirus-mediated human tissue kallikrein gene can enhance their resistance to hypoxia-induced apoptosis and decrease caspase-3 activity. Moreover, treatment of AMI mice with these cells can improve ventricular function, reduce infarct size, attenuate ventricular remodeling, and promote angiogenesis [196]. Ham et al. found that, compared with conventionally cultured MSCs, mice treated with let-7b-transfected MSCs exhibited improved left ventricular function and increased microvascular density. This effect is achieved by protecting the transplanted MSCs from apoptosis and autophagy through direct targeting of the caspase-3 signaling pathway by let-7b [197]. These studies indicate that MSCs can reduce cardiomyocyte apoptosis after MI by decreasing the activity of caspase-3.

Not only MSCs have anti-apoptotic effect, but MSCs-EXO can also reduce the oxidative stress after MI and improve the anti-apoptotic ability of cardiomyocytes in the peri-infarction area. Regardless of the choice of intramuscular injection [198, 199], intra-coronary injection [200], intravenous injection (IT) [201, 202] or intracardiac injection [203], MSCs-EXO can play a role in reducing the apoptosis of endogenous cardiomyocytes. Pretreated or genetically modified MSCs-EXO have better anti-apoptotic ability than untreaded or unmodified MSCs-EXO. For example, MSCs under anoxia can release exosomes enriched with miR-210 and reduce cardiomyocyte apoptosis after MI [204]. MSCs-EXO can ameliorate myocardial injury and reduce apoptosis after MI by activating S1P/SK1/S1PR1 signaling and promoting M2 macrophage transformation [205]. Zhang et al. [206] also showed that the miR-24 released level of BM-MSCs-EXO hypoxic preconditioning was significantly higher than that of normal-oxygen pretreating of BM-MSCs. After the injection of hypoxic BM-MSCs-EXO, the level of miR-24 in AMI mice showed significant differences. The MI size was reduced and the left ventricular function improved. Other studies have shown that genetically modified MSCs-EXO with miR-125b can target SIRT7 binding to prevent myocardial injury and apoptosis reduced by I/R [207]. Luo et al. showed that AD-MSCs-EXO with overexpression of miR-126 could effectively reduce cardiomyocyte apoptosis in myocardial border area and prevent myocardial damage [208]. The above studies indicate that MSCs-EXO exhibits a diverse array of delivery pathways, a remarkable homing capacity, and significant anti-apoptotic properties. Furthermore, they provide a valuable research foundation for the potential clinical application use of MSCs-EXO in the treatment of cardiomyocyte apoptosis after MI.

During MI-induced cell apoptosis, not only does oxidative stress play a role, but there is also a loss of ATP and NADH. Treatment with MSCs-EXO increased ATP and NADH levels, reduced oxidative stress, and enhanced activation of the PI3 K/AKT pro-survival signaling pathway. Additionally, MSCs-EXO treatment decreased the phosphorylation of c-JNK, a key activator of pro-apoptotic signaling [209]. This suggests that MSCs-EXO reduces apoptosis at least in part by restoring the bioenergetics of target cardiomyocytes and reducing oxidative stress.

The inflammatory response has been comprehensively detailed in the preceding Sect." Immunomodulatory and anti-inflammatory"and will not be reiterated here.

The new small capillaries are hollow tubular structures surrounded by ECs which are supported by pericytes. The outsourced basal cells and extracellular matrix (ECM) can further enhance the structural stability of small capillaries [210]. In the physiological state, ECs maintain inactive and the surrounding basal cells and pericytes also maintain the normal order of ECs to perform its functions. However, when the environmental and mechanical stresses in the injured tissue change, these stable structures are destroyed [211]. Preexisting ECs in the infarct margin area will produce initial new capillaries by budding [146]. The budding process begins with the breakdown of basement membrane by proteolytic enzymes and the isolation of pericytes. After that, ECs can be activated. With the help of VEGF and other factors, activated ECs escaped from the original capillaries and invaded the temporary scaffold matrix to participate in budding. Their cell characteristics and molecular expression profiles are altered, and they will differentiate into tip and stalk cells with different morphology and functions [212, 213]. The tip cells have a large number of filamentous feet, which are mainly responsible for migration and coordinating the direction of branch formation. During bud elongation, stalk cells proliferate and form new primitive capillary lumen with the proliferation of stem tip cells [213, 214]. The stable budding then begins to transform into mature blood vessels and fuse with existing capillaries. The ECM also gathers around the nascent original capillaries and recruits pericytes to cover the ECs [215]. Ultimately, the blood vessels achieve stability and blood flow normalizes.

Homing ability is the basis for MSCs to participate in angiogenesis [216]. MSCs can migrate and adhere to ischemic tissues through differentiation, direct contact or paracrine, and play a role in promoting angiogenesis [217, 218]. For example, hypoxia and reoxygenation can accelerate the state recovery of MSCs, improve the expression of pro-survival genes and various nutritional factors in MSCs, and indirectly increase the homing ability and angiogenesis of MSCs [219, 220]. In addition, hypoxia pretreating of BM-MSCs can enhance their migration capacity by activating Kv2.1 channels and FAK [221]. The above studies indicate that hypoxia pretreating can improve the survival rate of transplanted MSCs in the infarct junction and promote homing ability and angiogenesis. It provides a good experimental basis and theoretical basis for us to further explore the relationship and mechanism between hypoxia pretreating, homing ability and angiogenesis of MSCs.

In addition to hypoxia, several genes related to angiogenesis can also be directly transferred into MSCs to enhance the angiogenic ability of MSCs. Using MI as an example, MSCs transfected with HGF or VEGF can promote angiogenesis and reduce fibrosis, thereby improving ventricular function in MI-induced porcine models [222]. Compared with MI mice injected with ordinary MSCs, MI mice injected with Ang-1 overexpressing MSCs had a better effect of angiogenic (approximately 11–35% more than the control group) [223]. Other studies have shown that basic fibroblast growth factor can control the migration of MSCs and improve the integration of MSCs with ECs to promote angiogenesis [224, 225]. These studies collectively suggest that appropriate genetic engineering of MSCs is essential to enhance their angiogenic potential.

The regenerative capacity of MSCs is also associated with their differentiation potential. In addition to osteogenic, chondrogenic, and adipogenic differentiation potential, MSCs are also capable of differentiating into steroidogenic cell lineages [226], myogenic cells [227], hepatocyte-like cells [228], and cardiomyocytes [229]. However, whether MSCs can differentiate into ECs is still controversial in current studies. At present, only a few studies support that MSCs can differentiate into ECs-like cells [230–232]. Most scholars believe that MSCs and MSCs-EXO can promote angiogenesis mainly because they can interact directly with damaged ECs or in paracrine form [233, 234]. After MSCs come into direct contact with the damaged ECs, tunneling nanotube-like structures are formed. This structure allows MSCs to move frequently and almost unidirectionally towards the damaged ECs, thereby resulting in the rescue of aerobic respiration and protection of ECs from apoptosis [235]. In the paracrine studies of MSCs and MSCs-EXO for the promotion of tissue regeneration, MSCs-EXO has been the most extensively investigated in recent years. The current research mainly focuses on the following three aspects: (1) Combining MSCs-EXO with some new molecular materials can improve the therapeutic effect of MSCs-EXO. For example, if MSCs-EXO overexpressing Islet-1 is combined with Ang-1 gel, the retention of MSCs-EXO in the heart can be increased, and its anti-apoptotic effect can be further enhanced to promote angiogenesis, to enhance the anti-apoptotic effect and promoting angiogenesis [236]. After the injection of magnetic iron oxide nanoparticles with MSCs-EXO mixed reagents into MI's heart, the inflammatory period can be transformed into a repair period in advance under magnetic guidance. This results in a reduction of apoptosis, an alleviation of fibrosis, the promotion of angiogenesis, and the recovery of ventricular function [237]. After all, MSCs-EXO mixed new materials are exogenous implants, and whether they have adverse effects on the human body still needs long-term follow-up observation, so we still need to be cautious about their use. (2) MSCs-EXO (or pretreating of MSCs-EXO) were modified by cell engineering will promote angiogenesis and tissue repair. For example, up-regulation of CXCR4 expression in MSCs-EXO can promote angiogenesis through AKT signaling pathway [238]. MSCs pretreated with atorvastatin can improve the survival rate of ECs and increase the expression of VEGF and ICAM-1 gene to improve the therapeutic effect [239]. However, it is unclear whether modification or pretreatment affects the genetic properties of MSCs-EXO, so its clinical application remains to be further investigated. Optimizing the delivery mode of MSCs-EXO can improve the utilization rate and promote angiogenesis. The emergence of new delivery methods, such as intra pericardial delivery [240] and exosome spray [241], has changed the traditional concept that MSCs-EXO can only be used by injection. These advancements may pave the way for new therapeutic strategies and could become a major focus of future research and development in the field of MSCs-EXO.

## Therapeutic applications in disease models

**Fig. 7:**
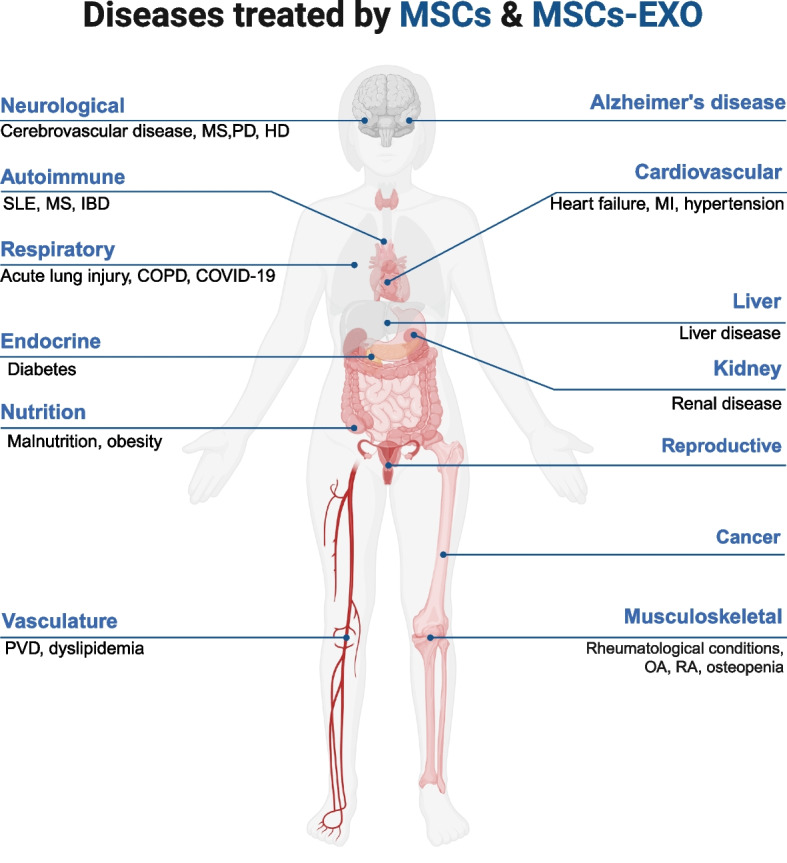
Overview of MSCs and MSCs-EXO applications in Disease Models. MSCs and MSCs-EXO can exert therapeutic effects in a wide range of diseases, including those affecting the nervous system, respiratory system, circulatory system, digestive system, urinary system, reproductive system, musculoskeletal system, immune system, and many others. MSCs: mesenchymal stem cells; MSCs-EXO: MSCs-derived exosomes; MS: multiple sclerosis; PD: parkinson's disease; HD: huntington's disease; SLE: systemic lupus erythematosus; IBD: inflammatory bowel disease; COPD: chronic obstructive pulmonary disease; PVD: peripheral vasculature disease; MI: myocardial infarction; OA: osteoarthritis; RA: rheumatoid arthritis

Currently, MSCs and MSCs-EXO have been extensively utilized in clinical studies owing to their promising therapeutic effects. This section will summarize their applications in CVDs, neurological disorders, ADs, MSDs, and other conditions (Fig. 7).

### Therapeutic applications in CVDs

CVD, a broad term encompassing disorders that affect the heart and blood vessels, is a significant cause of morbidity and mortality, posing a major threat to human health and well-being. A retrospective study from 204 countries reported that the number of patients with CVD increased from 271 to 523 million, and the number of deaths with CVD increased from 12.1 million to 18.6 million between 1990 and 2019 [242]. This is a huge challenge for the global health system. MI is an important component of CVD. MI refers to the condition where reduced coronary blood flow, due to various causes, results in ischemia and hypoxia of cardiomyocytes, leading to necrosis in the myocardial region supplied by the affected coronary artery [243]. MI can be classified into AMI and chronic MI (CMI) based on the course and nature of the disease. The primary cause of AMI is acute thrombosis. When a coronary artery is abruptly occluded by an acutely dislodged thrombus, myocardial necrosis can occur within minutes to hours. However, CMI is a long-term pathophysiological process characterized by the gradual expansion of coronary atherosclerotic plaques, leading to progressive luminal narrowing and resultant myocardial ischemic injury. The exact timeframe for CMI development remains variable [244]. Cardiomyocyte apoptosis and subsequent inflammatory response will lead to local tissue fibrosis, scar tissue formation and poor ventricular remodeling. They can have adverse effects on the regeneration of the heart, and some patients may progress to HF or even death [245]. All the above pathophysiological processes occur throughout the progression of the disease, both in the AMI and CMI. Therefore, it is of great significance to explore a new biologic therapy to alter or reverse these pathophysiological processes.

In recent years, MSCs and MSCs-EXO have emerged as promising therapeutic modalities in the clinical treatment of CVDs, including MI. We have summarized the clinical application of MSCs in Table 1 and found that BM-MSCs are the most commonly utilized type for the treatment of MI, followed by AD-MSCs and UC-MSCs. In contrast, DP-MSCs are primarily used in basic research, with clinical studies predominantly focusing on pulp and bone regeneration following tooth injury. Although a large number of preclinical studies have shown that MSCs have a positive effect on the treatment of MI, the results of existing clinical trials have been polarized. For example, after autologous BM-MSCs transplantation in 10 AMI patients who received standard therapy, Bodo et al. found that, compared with MI patients receiving standard therapy, those in the cell therapy group exhibited a significant reduction in the percentage of infarct size relative to the total left ventricular area and a significant increase in the velocity of ventricular wall motion in the border zone [246]. This suggests that BM-MSCs may possess reparative effects on cardiomyocytes following MI. According to another study involving 69 patients with AMI who were randomized to receive either BM-MSCs or saline following percutaneous coronary intervention (PCI) within 12 h of symptom onset, left ventricular function was significantly improved in the BM-MSCs treatment group (n = 34) [247]. With the deepening of research, more and more clinical studies have confirmed the safety of MSCs in clinical application and its protective effect on myocardial tissues after MI [248, 249]. However, some studies have shown that MSCs have a limited therapeutic effect on MI. For example, Wollert et al. [250] showed that patients treated with MSCs did not show a significant increase in left ventricular ejection fraction (LVEF) compared to controls. In addition, according to another study in Norway, Patients in the acute stage of MI did not significantly improve left ventricular function after intra coronary injection of BM-MSCs [251]. Other studies have demonstrated that, while patients treated with MSC injections exhibited improved LVEF, reduced left ventricular end-systolic volume, enhanced stroke output, and smaller MI scar areas, there were no significant differences in overall mortality or cardiovascular mortality compared to those receiving conventional therapy [252]. Although the above data indicate that MSCs are not ideal for the clinical treatment of MI, most studies show that the effect of MSCs on the clinical treatment of MI cannot be ignored, compared with untreated group, LVEF and ventricular function were improved significantly. We analyzed the reasons for the large heterogeneity in the above studies as follows: (1) The total number of participants in the study was too small for statistical analysis; (2) Different amounts of imported MSCs or different injection sites lead to heterogeneity of treatment outcomes [253]; (3) Different preparation techniques and algebras of MSCs lead to different therapeutic effects; (4) There are differences in the treatment time window.

**Table 1:** Clinical application of MSCs and MSCs-EXO in CVDs

**Nation**	**NCT**	**Duration**	** type**	**MSCs**	**source**	**Sample size**	**Experimental group**			**Control group**			**Time**	**Primary outcomes**	**Result**	**References**
							**Sample size**	**Male**	**Therapy**	**Sample size**	**Male**	**Therapy**				
China		Nov 2002– May 2003	AMI	BM-MSCs	autologous	69	34	32	48-60×10^9^ BM-MSCs therapy	35	34	Received placebo injection	6m	LVF	Improvement in LVF.	[247]
USA	NCT 00114452	Mar 2005– May 2006	AMI	BM-MSCs	autologous	53	34	28	E1: 0.5×10^6^ BM-MSCs/kg; E2: 1.6×10^6^ BM-MSCs/kg; E3: 5×10^6^ BM-MSCs/kg.	19	15	Standard therapy	6m	LVEF; LVV; MACE	Improvement in LVEF and reversal of ventricular remodeling. Similar MACE.	[248]
Korea	IRB SCH 2011 -006	Jan 2012– May 2015	STEMI	BM-MSCs	autologous	26	14	14	7.2±0.90×10^7^ BM-MSCs therapy	12	12	Standard therapy	1y	LVEF	Improvement in LVEF.	[249]
Iran	IRCT20201116049408N1	Jan 2021–Nov 2021	STEMI	WJ-MSCs	allogeneic	65	40	36	E1:10×10^6^ WJ-MSCs injection; E2: WJ-MSCs double injection (10 days apart).	25	22	Standard therapy	6m	LVEF	Significant improvement in LVEF and the effect can be enhanced by second therapy.	[254]
China	NCT 04421274	Mar 2008–Jul 2010	STEMI	BM-MSCs	autologous	43	21	20	2.0-5.0×10^6^ BM-MSCs therapy	22	19	Standard therapy	1y	Myocardial activity; LVF; MACE	No significant improvement of LVF and myocardial vitality. No MACE.	[255]
Korea	NCT01392105	May 2007– Sept 2010	AMI	BM-MSCs	autologous	58	30	27	7.2±0.90×10^7^ BM-MSCs therapy	28	25	Standard therapy	6m	LVEF; MACE	Modest improvement in LVEF and no MACE.	[256]
China	NCT01291329	Feb 2011– Jan 2011	AMI	WJ-MSCs	allogeneic	116	58	55	6×10^6^ WJ-MSCs therapy	58	51	Received placebo injection	18m	MACE; LVEF; Myocardial viability and perfusion of the infarcted region.	No difference in MACE; Improvement of LVEF and myocardial viability and perfusion.	[257]
India	NCT00883727	July 2009– Nov 2011	STEMI	BM-MSCs	allogeneic	20	10	10	2×10^6^ BM-MSCs therapy	10	8	Received placebo injection	6m	LVEF; IS; MACE	Improvement of LVEF; No difference in MACE and IS.	[258]
China		Nov 2002– May 2003	AMI	BM-MSCs	autologous	69	34	32	8×10^9^ BM-MSCs therapy	35	34	Received placebo injection	6m	LVF; MACE	Improvement of LVF. No difference in MACE.	[259]
USA		July 2009– Nov 2011	OMI	MSCs	autologous	16	8	7	MSCs therapy	8	8	Standard therapy	6m	LVF; NYHA; MACE	Improvement in LVF and NYHA; No difference in MACE.	[260]
China		May 2008– Nov 2009	STEMI	BM-MSCs	autologous	43	21	21	3.08±0.52×10^7^ BM-MSCs therapy	22	19	Standard therapy	2y	Myocardial activity and perfusion;^、^LVEF	No improvement of LVEF and no different in myocardial activity and perfusion.	[261]
USA		Sept 2009– Jul 2013	ICM	BM-MSCs	autologous	30	19	18	BM-MSCs therapy	11	10	Standard therapy	1y	MACE; 6MWD; MLHFQ; LVV; LVEF	No MACE occurred; Improvement of 6MWD and MLHFQ. No improvement of LVEF and LVV.	[262]
Greece			MI	BM-MSCs	allogeneic	22	11	11	1-2×10^6^ BM-MSCs therapy	11	11	Standard therapy	4m	Ventricular wall motion scoring index; Myocardial contractility; uptake of Tc(99m) sestamibi.	Significantly lower ventricular wall motion score index; Improvement of myocardial contractility; Recovery of uptake of Tc sestamibi (99 m).	[263]
Netherlands	NTR1553	2008-2013	STEMI	BM-MSCs	autologous	54	9	7	10×10^6^ BM-MSCs therapy	45	35	Historical comparison	5y	LVF; MACE	LVF improved within 5 years. No difference in MACE.	[264]
China			AMI	BM-MSCs	autologous	16	8	7	1.22±1.77×10^7^ BM-MSCs therapy	8		1.32±1.76×10^7^ BM-MSCs for RCA infarction.	6m	LVF; MACE; myocardial perfusion; viable cardiomyocytes.	No MACE occurred; Improvement of LVF and myocardial perfusion; All the surviving cardiomyocytes were detected.	[265]
China		Jul 2008– Oct 2009	AMI	BM-MSCs	autologous	58	28	19	20×10^7^ BM-MSCs therapy	30	16	Received placebo injection	6m	LVEF; LVDd; IS; MACE	No significant differences in LVEF, LVD and IS. Decrease in MACE.	[266]
USA	NCT0076545	Mar 2008– Mar 2013	AMI	BM-MSCs	autologous	100	45	41	1.9±1.5×10^6^ BM-MSCs therapy	55	41	Standard therapy	1y	LVEF	Minor improvement in LVEF.	[267]
USA	NCT00442806		STEMI	AD-MSCs		13	9	7	17.4±4.1×10^6^ AD-MSCs therapy	4	4	Standard therapy		LVEF; MACE; myocardial perfusion.	Improvement of LVF and myocardial perfusion; No MACE occurred.	[268]
Germany	NCT00669227	Oct 2005– Jan 2009	AMI	BM-MSCs	autologous	42	29		324×10^6^ BM-MSCs therapy	13		Received placebo injection	3y	LVEF	Improvement of LVEF within3 years.	[269]
Germany, Switzerland	NCT00279175	Apr 2004– Oct 2005	AMI	BM-MSCs	autologous	204	101	84	236±174×10^6^ BM-MSCs therapy	103	83	Standard therapy	2y	LVEF; MACE	Significant improvement of LVEF; Reduction of MACE.	[270]
Belgium	NCT00264316	May 2003– Dec 2005	STEMI	BM-MSCs	autologous	67	33	28	476×10^6^ BM-MSCs therapy	34	28	Standard therapy	4m	LVEF; IS; myocardial perfusion and oxidative metabolism.	No significant differences in LVEF; Reduction of IS; No difference in both myocardial perfusion and oxidative metabolism.	[271]
Romania			STEMI	BM-MSCs	autologous	18	9	7	1.66±0.32×10^9^ BM-MSCs therapy	9	5	Received placebo injection	4y	The plaque count; PB; CCS	Significant reduction of plaque count, PB and CCB.	[272]
Poland	BN-001/122/05	Dec 2010– Jun 2014	STEMI	BM-MSCs	autologous	34	15		8.37×10^6^ BM-MSCs therapy	19		Standard therapy	1y	LVEF; BNP; MACE; troponin T levels	Improvement in LVEF and decrease in troponin T levels and BNP; No MACE occurred.	[273]
Norse	NCT00199823	Dec 2003– Jun 2005	STEMI	BM-MSCs	autologous	28	15	12	BM-MSCs therapy	13	10	Received placebo injection	6m	LVF	No Significant improvement of LVF.	[274]
Germany		Sept 2005– May 2006	AMI	BM-MSCs	autologous	60	30	20	24.6±9.4×10^8^ BM-MSCs therapy	30	22	Standard therapy	18m	LVEF	Improvement in LVEF.	[275]
Norse	NCT 00199823	Sept 2003– May 2005	STEMI	BM-MSCs	autologous	100	50	42	54-130×10^6^ BM-MSCs therapy	50	42	Standard therapy	6m	maximal symptom-limited bicycle ergometer exercise tests; the Short Form 36 health survey.	Significantly i improvement of the response of exercise time and heart rate to exercise.	[276]
USA			MI	BM-MSCs	autologous	49	23	21	Below 60 years of age: a certain amount of MSCs therapy	29	23	Aged 60 and above: equal amount of MSCs therapy	1y	6 MWD; MLHFQ; IS.	Improved 6 MWD and MLHFQ, and reduced IS.	[277]
USA	NCT 00893360	May 2009– Dec 2010	AMI	MSCs	autologous	31	23		12.5-25×10^6^ BM-MSCs therapy	8		Standard therapy	1y	LVEF; scar mass; ESV; viable heart mass	Significantly reduction in scar mass and increases in viable heart mass. ESV and LVEF did not differ.	[278]
USA	NCT 02013674	Apr 2014– May 2010	ICM	BM-MSCs	autologous	30	30	27	E1:2×10^7^ BM-MSCs; E2: 10×10^7^ BM-MSCs.				1y	Scar size; MACE	Both cell doses reduced scar size, only the 100 million doses increased LVEF.	[279]
USA	NIHMS559241		chronic ischemic cardiomyopathy	MSCs	autologous allogeneic	30	30		E1: 2×10^7^ autologous BM-MSCs; E2: 2×10^7^ allogeneic BM-MSCs.				13m	Scar size; LVEF	Improvement of LVEF and decrease in scar size.	[280]
USA	NCT01087996	Apr 2010– Sept 2011	MI	BM-MSCs	autologous	31	31	27	E1: 2×10^7^ BM-MSCs; E2: 1×10^8^ BM-MSCs; E3: 2×10^8^ BM-MSCs.				13m	MACE; 6 MWD; MLHFQ; exercise peak VO2; LVV; LVEF; EED	No MACE occurred; Improvement of 6 MWD and MLHFQ; No improvement of exercise peak VO2 and LVEF; Decrease in EED.	[281]
China	NCT 04056819	Aug 2019– Aug 2020	STEMI	UC-MSCs	allogeneic	6	6	6	UC-MSCs therapy: the first dose (1×10^7^) on the first day and the second dose (1.9×10^7^) on the second day.				1y	LVF; MACE; NT-pro BNP; the wall motion score.	Improvement in LVEF and decrease in NT-pro BNP and the wall motion score. No MACE occurred**.**	[282]
China		May 2010– June 2011	MI	UC-MSCs	allogeneic	15	15	9	E1: 3×10^6^ UC-MSCs; E2: 4×10^6^ UC-MSCs; E3: 5×10^6^ UC-MSCs.				2y	LVEF; LVD; IS; MACE	No MACE occurred, improvement in LVEF and LVD and decrease in IS.	[283]
Germany		Mar 2019– Mar 2020	MI	WJ-MSCs	allogeneic	1	1	1	12.5×10^6^ WJ-MSCs coronary artery implantation therapy				1y	Scar mass; LVEF; MACE	9% reduction in scar mass in the treated area, no MACE occurred and improvement in LVEF.	[284]

*WJ-MSCs* Wharton jelly-derived mesenchymal stem cells, *STEMI* ST-segment elevation myocardial infarction, *MI* myocardial infarction, *OMI* old myocardial infarction, *LVEF* left ventricular ejection fraction, *MACE* major adverse cardiovascular events, *LVV* left ventricular volumes, *LVF* left ventricular function, *NYHA* New York Heart Association, *LVDd* left ventricular end-diastolic dimensions, *IS* infarct size, *ESV *end-systolic volume, *PB* plaque burden, *CCS* congestion score, *BNP* brain natriuretic peptide, *6 MWD* 6-min walk distance, *MLHFQ* Minnesota Living With Heart Failure Questionnaire, *EED* early enhancement defect, *LVD* left ventricular dysfunction, *m* month, *y* year, *USA* United States of America

Data sources -- published literature

It is noteworthy that our review of clinical studies revealed that the majority of trials utilized coronary artery administration, whereas IT and transendocardial stem cell injection (TESI) were employed in only a minority of cases. In recent years, stem cell pericardial implantation technology has emerged as a novel approach and has gradually garnered attention. Although coronary artery administration remains the predominant method for stem cell therapy in MI, significant variability in clinical outcomes persists. A study by HOPP et al. demonstrated that, compared with the control group receiving sham injections, the experimental group of 15 patients who received intra-coronary injections of BM-MSCs did not exhibit significant improvements in cardiac function, and the reduction in infarct size was not statistically significant [274]. Although a few studies have indicated that MSCs treatment may be ineffective, it is important to note that no serious adverse reactions have been observed following autologous transplantation of MSCs. This suggests that, despite the controversy surrounding its efficacy, the safety of MSCs in clinical applications is well-established. In addition to autologous transplantation, MSCs are also suitable for allotransplantation. While allogeneic MSCs exhibit low immunogenicity, immune rejection may still occur in some cases. For instance, it has been demonstrated that recipients of completely mismatched allogeneic MSCs may develop memory T cells that can trigger immune rejection [285]. The survival time of allogeneic MSCs post-transplantation is typically shorter than that of autologous MSCs, which may influence therapeutic outcomes. Additionally, MSCs derived from different tissue sources exhibit variations in biological properties, culture characteristics, and differentiation potential, potentially leading to differences in therapeutic efficacy.

Although MSCs-EXO has shown certain potential in clinical studies of atomized inhalation in the treatment of novel coronavirus pneumonia and other diseases [286], its application in clinical studies is still insufficient compared with MSCs. The administration route and dosage optimization of MSCs-EXO need to be further explored. In addition, MSCs-EXO is rapidly cleared in the body, has poor stability, and is susceptible to temperature, pH and mechanical stimulation [287]. Therefore, it is crucial to develop effective delivery systems to enhance their persistence in vivo and to establish appropriate preservation methods to ensure their efficacy and stability. Future research should further investigate the clinical applications of MSCs and MSCs-EXO to fully realize their therapeutic potential.

### Therapeutic applications in neurological disorders

Neurological disorders rank fourth in the global disease burden [288]. Its common clinical manifestations include sensory and motor dysfunction, as well as impaired cognitive function. Given the limited regenerative capacity of nerve cells following damage, these conditions often result in long-term or even lifelong challenges for patients [289]. In therapeutic interventions, the timely and effective repair of injured neurons is crucial for maintaining normal neural function. MSCs represent an ideal cell source for neural regeneration and repair. By secreting a diverse array of bioactive factors, including miRNAs, proteins, and growth factors, MSCs not only promote neuronal survival and nerve regeneration but also inhibit inflammatory responses, thereby significantly enhancing neural function [290].

In recent years, MSCs and MSCs-EXO have been widely used as an alternative therapy for the clinical treatment of neurological diseases. We summarized the recent clinical applications of MSCs and MSCs-EXO in neurological diseases in Table 2. Spinal muscular atrophy (SMA) is a neurodegenerative disorder resulting from mutations in the SMN1 gene [291]. In patients with SMA, the capacity of MSCs to cross the blood–brain barrier can be leveraged to maximize the therapeutic efficacy of IT [292]. Meanwhile, intravenous injection (IV) is more appropriate for targeting organs such as the lungs, spleen, liver, bone marrow, and thymus, which are characterized by their propensity for cellular diffusion [293]. Studies have demonstrated that IV of MSCs promotes the release of neurotrophic factors and cytokines, facilitating their entry into pathological tissues. This approach has been shown to significantly improve the tibial nerve motor amplitude response in patients with SMA, thereby demonstrating the tolerability and safety of MSCs in SMA treatment [294].

**Table 2:** Clinical application of MSCs and MSCs-EXO in Neurological Disorders

Nation	NCT	Duration	type	MSCs	source	Sample size	Experimental group			Control group			Time	Primary outcomes	Result	References
							**Sample size**	**Male**	**Therapy**	**Sample size**	**Male**	**Therapy**				
Iran	NCT02855112	2015	SMA	AD-MSCs	autologous	10	5		IV1×10^6^, 2×10^6^ and 5×10^6^ cells/kg/15 days, 3 times	5		Not received intervention	24m	AE; the side effects; HINE; Ballard score; EDX	No AE and the side effects occurred; HINE, Ballard score and EDX improved.	[294]
USA	NCT0335536	Aug 2018–Jun 2020	MS	MSC-NPs	autologous	54	27	9	Six IT injections of 1×10^7^ MSC-NPs	27	7	Six IT injections of saline	12m	EDSS Plus	No difference in EDSS Plus improvements.	[295]
Korea	20150196587		Asian patients with traumatic optic neuropathy	UC-MSCs	allogeneic	4	4		5×10^7^ UC-MSCs/mL subtendinous transplantation				12m	AE and vision	Visual acuity improved; No AE occurred.	[296]
Canada			MS	MSCs	autogenous	20	9	6	Plasma suspension 5% albumin 10% DMSO and 1-2×10^6^/kg	11	7	Plasma suspension 5% albumin 10% DMSO	48w	Cortical motor excitability and motor conduction	MSCs did not significantly improve clinical and neurophysiology.	[297]
USA	NCT03635450	Mar 2019-Aug 2020	HIE	UC-MSCs	allogeneic	5	5	2	IV 2×10^6^cells/kg/ time, 2 times, 2 months apart				12m	Bayley's score	It was well tolerated but had a low developmental score.	[298]
Israel	NCT02166021		MS	BM-MSCs	autologous	48	30		1×10^6^/kg IV(n=15),1×10^6^/kg IT(n=15)	14		Same amount of saline	12m	NF-L and CXCL13 in the CSF.	NF-L decreased significantly, while CXCL13 decreased without any difference.	[299]
Türkiye	NCT04877067	Apr 2019-Apr 2021	toxic optic neuropathy via	WJ-MSCs	allogeneic	18	6		only WJ-MSCs in both eyes	12		WJ-MSCs and rEMS combination in one eye; only rEMS in one eye.	4m	BCVA; FPDI; VEP; GCC	WJ-MSC and rEMS application can improve BCVA, FPDI, and reduce VEP and GCC.	[300]
Poland		2015-2018	ALS	BM-MSCs	autologous	8	8	5	10×10^6^ IT/3 months, 3times				6m	ALSFRS-R;	The progression of the disease slows down.	[301]
Jordan	NCT04288934		SCI	BM-MSCs, UC-MSCs	autologous, allogeneic	20	11	9	10×10^8^ BM-MSCs/month, 3 times	9	8	10×10^8^ UC-MSCs/month, 3 times	22.65m	AE; ASIA total score;	No long-term AE; The ASIA score improves significantly.	[302]
Spanish	NCT03003364		SCI	WJ-MSCs	allogeneic	10	10	7	10×10^5^±20% WJ-MSCs and saline were cross-injected twice				6m	AE; ASIA total score; MEP; SEP; Ampiveen questionnaire; Rome III diagnostic questionnaire; Rome III diagnostic questionnaire.	Acupuncture sensation improved. Only other clinically relevant effect was observed at the individual level. Not observed movement function, cramps, MEP, SEP and bowel function, quality of life index changes or independence.	[303]
Iran	IRCT20160110025930N	2018	SCI	BM-MSCs	autologous	3	3		A combination of OEC and 1.5×10^6^ BM-MSCs/mL was received by lumbar puncture.				2y	AE, ASIA; SCIM III	No AE occurred; The AIS and SCIM III scores did not show any change.	[304]
China	ChiCTR-TRC-14005093	Jan 2015-Dec 2017	TON	UC-MSCs	allogeneic	20	10	9	MSCs-gelatin sponge scaffolds were implanted and the optic canal was decompressed.	10	8	Only the optic canal was decompressed	6m	AE; BCVA	No AE occurred; No statistical difference in the improvement of BCVA.	[305]
Malaysia	NCT01461720	May 2012-Mar 2017	MCA infarct	BM-MSCs	autologous	17	9	8	2×10^6^ cells/kg BM-MSCs	8	2	Standard medical care	12m	NIHSS, mRS, BI and total infarct volume	No improvement in neurological recovery or function in the BM-MSCs group, only a difference in infarct size.	[306]
China	ChiCTR1800016554	Aug 2014-Dec 2016	CP	UC-MSCs	allogeneic	40	20	14	IV 4.5~5.5×10^7^/7 days, 4 times	20	14	IV 50 ml of NS with 1% human serum albumin	12m	AE, ADL, CFA and GMFM	No difference in AE; Significant improvements in ADL, CFA and GMFM.	[307]
Korean	NCT01716481	Nov 2012-Feb 2018	Ischemic Stroke	BM-MSCs	autologous	54	39	17	IV 1×10^6^ cells/kg	15	10	Injection of placebo	3m	AE; mRS Score; motor recovery	No AE occurred; No significant difference in the change of mRS Score； Recovery of lower extremity motor function.	[308]
China	ChiCTR210005502	2020	PD	OM-MSCs	autologous	5	5		5×10^7^/time, 2-3 times				6m	Clinically relevant experimental indicators	Neurological function is restored and the neuroinflammatory response is regulated.	[309]
Netherlands	NCT03356821	Feb 2020-Apr 2021	PAIS	BM-MSCs	autologous	10	10		45-50×10^6^ was administered intranasal				3m	vital signs, blood markers, and the occurrence of toxicity, AE, and unexpected cerebral abnormalities.	No AE occurred; No significant difference in blood inflammatory markers, and no unexpected cerebral abnormalities were observed.	[310]
USA	NCT03308565	Jun 2017-Oct 2021	SCI	AD-MSCs	autologous	10	10		IT AD-MSCs				2y	AE	No AE occurred.	[311]
Korea	NCT02054208	Mar 2014-Jun 2017	AD	UC-MSCs	allogeneic	9	3	1	1.0×10^7^ cells/2 mL/4 weeks,3 times	6	2	1.0 × 10^7^ cells/2 mL/4 weeks,3times	36m	AE	No serious AE occurred.	[312]
USA	NCT03280056	Aug 2017-Sept 2020	ALS	MSC-NTF	allogeneic	189	95	68	Received MSC-NTF	94	59	Received placebo	28w	AE; cerebrospinal biomarkers	No significant difference was observed in AE, and cerebrospinal biomarkers improved significantly.	[313]
9countries	NCT01745783	July 2012-July 2019	MS	BM-MSCs	autologous	144	69	28	Ex-vivo expanded 1-2×10^8^ /kg	75	29	Suspension medium placebo	48w	AE; GELs counted	No significant difference was observed in AE, and no significant difference was observed in GELs.	[314]
Spanish	NCT03173638		NA-AION	BM-MSCs	allogeneic	5	5	2	1.5×10^6^ cells/mL Intravitreal injection				1y	Ophthalmic examination	Visual and VEP improvement.	[315]
Korea	NCT01716481		Ischemic Stroke	MSCs	autologous	54	31	15	Received MSCs	13	9	Received placebo	90d	Motor function assessment and neuroimaging examination	MSCs can promote the recovery of motor function.	[316]
Jordan	NCT03326505		MS	UC-MSCs	allogeneic		20	12	First IV 50×10^6^, and one month later the IT 100×10^6^.	15	3	IV 50×10^6^ and IT 100×10^6^ for one time.	12m	AE; Whole transcriptome analysis	No AE occurred; The down-regulation error of inflammation-related and antigen presentation (HLA-B) genes has increased. TNF-α, TAP-1 and miR-142 were significantly downregulated.	[317]
Iran	NCT03795974		CP	UC-MSCs	allogeneic	72	36	21	2×10^7^ cells IT	36	19	Small needle pricks to the lower back.	12m	GMFM-66	The differences in GMFM-66 are obvious.	[318]
USA	NCT03473301	Rom Api 2018	CP	UC-MSCs	allogeneic	68	23	17	2 × 10^6^ UC-MSCs /kg/3 months, 3 times	45	23	1 × 10^7^ /kg was injected at baseline and 12 months	6m and 12m	AE; GMFM-66	No significant difference in AE. The GMFM-66 score showed no difference in June, but there was a difference in December.	[319]
Iran	IRCT20200502047277N1	Oct 2020-Nov 2021	SCI	UC-MSCs-EXO	allogeneic	9	9	5	IT 300 μg of UC-MSCs-EXO				12m	ASIA; SCIM-III; NBD; MAS	ASIA pinprick, light touch, SCIM III total score, and NBD score have a significant improvement.	[320]
USA	NCT03799718	Mar 2019-Mar 2021	MS	BM-MSCs	autologous	18	18	10	IT 1×10^8^-1.25×10^8^, 3 times				38w	AE; CFS	No significant difference in AE. The neuroprotective factors in CSF increase and the inflammatory biomarkers decrease.	[321]
Poland		until Mar 2020	ALS	WJ-MSCs	allogeneic	67	67	37	230×10^6^ UC-MSCs /2 months, 3 times	67	37	From the PRO-ACT database	until Mar 2020	AE; ALSFRS-R scale	No AE occurred; The disease progresses slowly.	[322]
	NCT01716481		Stroke	MSCs	autologous	54	39		MSCs IV	15		Standard of care	3m	Fugl-Meyer Assessment	MSCs increased circulating EV and improved motor function and magnetic resonance imaging metrics.	[323]
Spanish	NCT01678534	Dec 2014-Dec 2017	AMASCIS	AD-MSCs	allogeneic	19	9	1	1×10^6^ cells/kg AD-MSCs	10	3	Injection of placebo	24m	AE, neurologic and systemic complications, and tumor development; mRS; NIHSS	No serious AE was observed; No significant difference in NIHSS and RS scores.	[324]
France	NCT00875654	2013	Subacute Ischemic Stroke	BM-MSCs	allogeneic	31	16	11	Received 1×10^8^ cells	15	11	Received no MSCs	2y	AE; Overall and motor recovery	There are significant differences in AE. Improve exercise recovery.	[325]
Israel	NCT04821479	2016-2019	ALS	BM-MSCs	autologous	20	20	16	1×10^6^ /kg 1 to 4 intravaginal injections, 3-6 months apart				12m	AE; ALSFRS-R	No AE occurred; ALSFRS-R score improved.	[326]

*SMA* Spinal muscular atrophy, *HINE* the Hammersmith Infant Neurological Examination, *EDX* electrodiagnostic, *MS* multiple sclerosis, *MSC-NPs* Mesenchymal stem cell-neural progenitors, *EDSS plus* Expanded Disability Status Scale plus, *HIE *hypoxic ischemic encephalopathy, *CSF *cerebrospinal fluid, *rEMS* repetitive electromagnetic stimulation, *BCVA* best corrected visual acuity, *FPDI* fundus perimetry deviation index, *VEP* visual evoked potential, *GCC* ganglion cell complex, *ALS* amyotrophic lateral sclerosis, *ALSFRS-R* the Revised Amyotrophic Lateral Sclerosis Functional Rating Scale, *SCI* Spinal cord injury, *MEP* motor evoked potentials, *SEP* sensory evoked potentials, *TON* traumatic optic neuropathy, *MCA* middle cerebral artery, *CP* cerebral palsy, *ADL* activities of daily living, *CFA* comprehensive function assessment, *GMFM *gross motor function measure, *PD* Parkinson's disease, *hOM-MSCs* hypoxia-preconditioned olfactory mucosa-MSCs, *PAIS* Perinatal arterial ischaemic stroke, *AD* Alzheimer's disease, *GELs* gadolinium-enhancing lesions, *NA-AION* acute non-arteritic ischemic optic neuropathy, *OEC* olfactory ensheathing cell, *AMASCIS* acute ischemic stroke, *d* day, *w* weeks, *m* month, *y* year, *USA* United States of America

Data sources -- published literature

MSCs treated by IV injection in combination with IT is considered a promising therapeutic strategy for treating neurological disorders. Lu Z et al. [327] investigated the use of long-established IV administration in combination with IT infusion of UC-MSCs for treating relapsing–remitting multiple sclerosis (RRMS) and neuromyelitis optica (NMO). During a 10-year follow-up of patients with NMO spectrum disorder (NMOSD), no adverse reactions indicative of intolerance, such as tumor formation or peripheral organ/tissue disease, were observed in either RRMS or NMOSD patients. This suggests that the combination of IV and IT administration is safe and feasible. Additionally, the study by Jamali F et al. [317] found that in patients with multiple sclerosis (MS) treated with UC-MSCs, those receiving two doses of IV and IT administration exhibited significantly greater improvement in clinical symptoms compared to the control group, which received a single dose of IV and IT treatment. The downregulation of inflammation-related genes, including TNF-α, TAP-1, and miR-142, indicates that the combination of IV and IT administration is effective. However, while the current study supports the effectiveness of combining IV and IT administration of MSCs for neurological disorders, further clinical trials are necessary to verify its long-term effects and determine the optimal dosing regimen.

### Therapeutic applications in ADs

ADs constitute a complex class of disorders whose etiology remains incompletely understood. Although there may be some similarities in the clinical presentation of different ADs, each condition exhibits its own distinct characteristics [328]. ADs are typically characterized by the activation of autoreactive T and B cell clones [329]. In these conditions, the immune system erroneously targets the body's own normal cells, resulting in a diverse array of symptoms and pathological changes.

SLE is one of the most prevalent ADs. Recent studies have demonstrated that MSCs and MSCs-EXO exhibit potent immunomodulatory effects in the treatment of SLE, influencing immune-related cells such as T cells, B cells, NK cells, and macrophages. The immunomodulatory properties and multidirectional differentiation potential of MSCs and MSCs-EXO make them valuable tools for treating ADs. However, due to concerns regarding the carcinogenic risks associated with genetic mutations, genetic instability, and excessive proliferation and differentiation of MSCs, MSCs-EXO is considered a more favorable option for future SLE treatments [330]. Studies have demonstrated that in a non-obese diabetic (NOD) rat model, treatment with AD-MSCs-EXO and BM-MSCs-EXO can effectively repair damaged neurons and astrocytes, specifically inhibit the overactivation of T and B lymphocytes, and mitigate autoimmune damage to islet cells. This results in improved cognitive function and reduced blood glucose levels [331]. We summarized the clinical application of MSCs and MSCs-EXO in ADs in the past 5 years (Table 3). In the trial conducted by Kamen DL et al. [350], six women with refractory SLE were treated with MSCs at a dose of 1 × 10^6^ cells/kg. The results indicated a downward trend in autoantibody levels, while the GARP-TGF-β index in B-cell serum increased. These findings suggest that MSCs exert a systemic immunomodulatory effect consistent with the observed clinical treatment outcomes. In a triple-blind, placebo-controlled, randomized trial conducted by Fernandez et al. [351], patients with secondary progressive multiple sclerosis (SPMS) were followed for 12 months after infusion of autologous AD-MSCs. Only one serious adverse event (a urinary tract infection, deemed unrelated to the study treatment) was observed. These findings suggest that the use of AD-MSCs in treating SPMS is both safe and feasible. However, data from this trial did not yield significant results for potential markers of therapeutic effect in immune testing. Therefore, larger studies are necessary to further investigate the therapeutic benefits of MSCs. In the trial conducted by Chun S et al. [352], seven patients with lupus nephritis (LN) received an intravenous infusion of 3.0 × 10^6^ cells/kg BM-MSCs, with no dose-limiting toxicity observed. Additionally, in animal models, BM-MSCs distribution in the kidneys persisted until day 7. These findings demonstrated the safety and tolerability of BM-MSCs in LN patients and identified 3.0 × 10^6^ cells/kg as the maximum tolerated dose for this patient population. Given the relatively limited number of patients with ADs currently treated with MSCs and MSCs-EXO, the existing results may be subject to bias. Therefore, it is essential to conduct further experimental validation in larger patient populations.

**Table 3:** Clinical application of MSCs and MSCs-EXO in ADs

**Nation**	**NCT**	**Duration**	**type**	**MSCs**	**source**	**Sample size**	**Experimental group**			**Control group**			**Time**	**Primary outcomes**	**Result**	**References**
							**Sample size**	**Male**	**Therapy**	**Sample size**	**Male**	**Therapy**				
Iran	NCT04559295	May 2019-Feb2021	Chronic Low Back Pain	BM-MSCs	autologous	80	40		BM-MSCs therapy	40		Placebo injection	12m	AE; the side effects; HINE; Ballard score; EDX	No AE and the side effects occurred; HINE, Ballard score and EDX improved.	[294]
Korea	NCT05011474	Apr 2021-July 2022	LBP	AD-MSCs	autologous	8	8	7	one-time intradiscal injection of 1 mL of 6.0×10^6^ cells/disc combined with HA.				6m	VAS, ODI, magnetic resonance imaging	The differences in VAS, ODI and magnetic resonance imaging were obvious.	[332]
France		2000-2019	ALL	BM-MSCs	allogeneic	51	19	9	BM-MSCs	32	18	Chemotherapy	15y	Pain, deformity of the feet and ankles; increased risk of recurrence or of new cancer.	no increased risk of recurrence or of new cancer in this population after an average follow-up of 15 years.	[333]
Scotland	CTRI/2019/11/022149	Dec 2019-Nov 2020	IOD	PB-MSCs	autologous	17	17	12	PRFM+PBMSCs	17	12	PRFM	6m	PI; GI; CAL; in the radiographic parameters; DD; DFP	DD and DFP decreased significantly; PPD, CAL score improved significantly; EHI score no statistically significant difference.	[334]
Spain	ISRCTN 13093912	Nov 2014-Mar 2019	intra-bony periodontal defects	DP-MSCs	autologous	20	10		XBS+10×10^6^ DP-MSCs/100 mg	10		XBS	12m	AE; CAL; PPD	No AE occurred. There was no significant difference in CAL and PPD.	[335]
Spain	NCT02172885	2012	OI	BM-MSCs	allogeneic	2	2	1	4×10^6^ BM-MSCs/kg/5-6months, 5 times				2.5y	bone parameters; quality of life	Improvement of bone parameters and quality of life	[336]
France		2005-2010	osteonecrosis	MSCs	allogeneic	50	26	17	Cell therapy	24	18	Simple, cell-free core decompression therapy	12y	clinical score	Visual analog scale and Constant score improved significantly.	[337]
China	NCT03955497	2018	OA	AD-MSCs	autologous	45	23	9	HTO+ 3×10^6^ cells/mL AD-MSCs	22	9	HTO	24m	WOMAC and VAS score	The severity of OA was significantly reduced in patients treated with MSCs.	[338]
Iran	IRCT20210423051054N1	2022	OA	UC-MSCs-EV	allogeneic	28	28	1	7×10^9^ particles/cc UC-MSCs-EV	28	1	Placebo injection	6m	VAS, WOMAC questionnaire, and Lequesne Index; AE	VAS, WOMAC questionnaire, and Lequesne Index were not significantly different; No AE occurred.	[339]
Chile	NCT03810521	Mar 2019-May 2019	OA	UC-MSCs	allogeneic	40	40	18	LD group (n=16) 2×10^6^ cells; MD group (n=16) 20×10^6^ cells; HD group (n=8) 80×10^6^ cells				6m	AE; Pritzker OARSI score; WOMAC questionnaire.	Pain and function improved, and the improvement was more pronounced at low and medium doses.	[340]
China	NCT0394357	2018	OA	AD-MSCs	allogeneic	11	6	0	6.7×10^6^ cells	5	0	4× 10^7^cells	1y	AE; WOMAC; VAS; SF-12.	No AE occurred; Pain and functional improvement.	[341]
China	NCT03990805	2019	OA	AD-MSCs	autologous	252	125	39	1×10^8^ AD-MSCs	127	26	An equal dose of saline	6m	AE; WOMAC; VAS	No AE occurred; Pain and functional improvement.	[342]
Iran	IRCT20080728001031N23	2018	OA	AD-MSCs	allogeneic	3	3		1×10^8^ AD-MSCs				6m	Laboratory data; MRI findings; clinical examination	Articular cartilage regeneration was significantly improved.	[343]
Korea	NCT0300071	Nov 2016-Feb 2018	OA	AD-MSCs	allogeneic	26	13	2	AD-MSCs+ MOWHTO	13	5	MOWHTO	24m	MRI evaluation; AE	The regeneration of articular cartilage was significantly improved; No AE occurred.	[344]
Italian	18-054	2018	OA	BM-MSCs	autologous	10	10	4	BM-MSCs				24m	IKDC; KOOS; VAS	Pain relief and functional improvement.	[345]
Spanish	NCT02365142	Aug 2016-July 2018	OA	BM-MSCs	autologous	60	30	17	100×10^6^ BM-MSCs+PRGF	30	16	PRGF, 3 times/week	12m	VAS; WOMAC; X-ray	VAS and WOMAC was significantly improved; X- ray saw no obvious change.	[346]
Brazil			OA	BM-MSCs	autologous	47	14	10	MSCs+PRP	33	14	MSCs (n=16); corticosteroid (n=17).	12m	KOOS; ROM; intra-articular cytokines	Improves and relieves OA symptoms.	[347]
USA	NCT03691909	Sept 2018-Sept 2020	RA	AD-MSCs	autologous	15	15	1	2×10^8^ IV for one time				52w	Hematology; renal function; liver function.	It is effective to improve joint function in RA patients.	[348]
China	NCT01547091	Jan 2011-Dec 2018	RA	UC-MSCs	allogeneic	119	59	6	LG+UC-MSCs	60	7	UC-MSCs	1y	Anti-inflammatory factor	Reduce inflammatory cytokines and increase anti-inflammatory factors	[349]

*SMA* Spinal muscular atrophy, *AE* adverse events, *HINE* the Hammersmith Infant Neurological Examination, *EDX* electrodiagnostic, *LBP* low back pain, *ALL* acute leukemia, *IOD* intraosseous defects, *PI* plaque index, *GI* gingival index, *CAL* clinical attachment level, *DD* defect depth, *EHI* Early wound healing index, *CAL* clinical attachment level, *OI* osteogenesis imperfecta, *XBS* xenogeneic bone substitute, *CAL* clinical attachment level, *PPD* probing pocket depth, *OI* Osteogenesis imperfecta, *OA* osteoarthritis, *ADs* autoimmune disease, *KOOS* knee injury and osteoarthritis outcome score, *MOWHTO* medial open-wedge high tibial osteotomy, *PRGF* platelet rich plasma, *PRP* platelet-rich plasma, *w* weeks, *m* month, *y* year, *USA* United States of America

Data sources -- published literature

### Therapeutic applications in MSDs

MSDs is a common group of diseases affecting human health, usually involving muscles, bones, tendons, ligaments, cartilage and related tissues. The main clinical symptoms include pain, stiffness, swelling and limited motor function [353]. This condition is often attributed to a variety of factors, including repetitive strain, poor posture, chronic stress, or traumatic injury [354]. OA and RA are among the most prevalent MSDs. OA can lead to intractable pain, limit daily activities, and significantly reduce patients'quality of life, although it is not inherently a fatal condition. RA, on the other hand, is a systemic autoimmune disease that can affect multiple joints throughout the body and is not secondary to OA. Currently, clinical treatment primarily focuses on pain relief, rehabilitation, or surgical intervention; however, these approaches are not effective in preventing the progression from OA to RA [355]. As a cell-based therapy, MSCs and MSCs-EXO demonstrate significant potential in the treatment of MSDs. MSCs have the capacity to differentiate into osteoblasts and chondrocytes, and they promote cartilage repair and regeneration through the secretion of various bioactive factors, including growth factors and cytokines [356]. MSCs-EXO can promote chondrocyte proliferation, inhibit cell apoptosis, and enhance the synthesis of cartilage matrix [357]. These characteristics underscore the significant potential of MSCs and MSCs-EXO in the cellular therapy of bone and cartilage diseases.

We have summarized the clinical applications of MSCs and MSCs-EXO in MSDs over the past five years (Table 4). Our analysis reveals that the most common method for administering MSCs or MSCs-EXO in the treatment of OA patients is direct intra-articular injection into the affected joint. This method allows the therapeutic agents to act directly on the damaged area, thereby enhancing treatment efficacy and minimizing side effects. While several alternative modes of administration are also under investigation, intra-articular injection currently remains the preferred strategy. Kim KI et al. [342] reported that 125 patients with OA received intra-articular injections of 1 × 10^8^ AD-MSCs. Compared to the control group, the injection group demonstrated significant meniscus regeneration and a reduction in knee pain. Chen CF et al. [341] also reported that six patients with knee OA received intra-articular injections of 6.7 × 10^6^ autologous AD-MSCs. The results demonstrated that patients experienced a significant reduction in knee pain, no serious adverse reactions were observed, and the quality of articular cartilage improved. Additionally, studies have found that when platelet-rich plasma (PRP) is administered in conjunction with MSCs, PRP significantly enhances the intra-articular retention time and survival rate of MSCs in an anti-inflammatory environment [358]. In the study by Lamo-Espinosa JM et al. [346], patients received intra-articular injections of 100 × 10^6^ autologous BM-MSCs in combination with PRP. Compared to the control group, which received only PRP injections, the experimental group demonstrated significant improvements in joint mobility function without any adverse reactions. A study by Vij R et al. [348] demonstrated that a single intravenous administration of autologous AD-MSCs significantly improved joint function in RA patients and, to some extent, reduced the levels of the inflammatory marker C-reactive protein (CRP). The treatment had minimal impact on renal and liver function, indicating that AD-MSCs are both safe and effective for treating RA. In patients with chronic low back pain, a single intra-discal injection of AD-MSCs combined with hyaluronic acid significantly alleviated pain symptoms and the imaging studies demonstrated a marked reduction in disc herniations [332]. Although these studies provide positive evidence, further large prospective, placebo-controlled trials are needed to validate the long-term efficacy and optimal dosing regimen of MSCs and MSCs-EXO.

**Table 4:** Clinical application of MSCs and MSCs-EXO in MSDs

Nation	NCT	Duration	type	MSCs	source	Sample size	Experimental group			Control group			Time	Primary outcomes	Result	References
							**Sample size**	**Male**	**Therapy**	**Sample size**	**Male**	**Therapy**				
**USA**	NCT03355365	Aug 2018–Jun 2020	MS	MSC-NPs	autologous	54	27	9	six IT injections of 1×10^7^ MSC-NPs	27	7	six IT injections of saline	12m	EDSS Plus	No difference in EDSS Plus improvements.	[295]
**Canada**			MS	MSCs	autogenous	20	9	6	Plasma suspension 5% albumin 10% DMSO and 1-2×10^6^/kg	11	7	Plasma suspension 5% albumin 10% DMSO	48w	Cortical motor excitability and motor conduction	MSCs did not significantly improve clinical and neurophysiology.	[297]
**9countries**	NCT01745783	July 2012-July 2019	MS	BM-MSCs	autologous	144	69	28	Ex-vivo expanded 1-2×10^8^/kg MSCs	75	29	Suspension medium placebo	48w	AE; Whole transcriptome analysis	No AE occurred; The down-regulation error of inflammation-related and antigen presentation (HLA-B) genes has increased. TNF-α, TAP-1 and miR-142 were significantly downregulated.	[314]
**Jordan**	NCT03326505		MS	UC-MSCs	autologous		20	12	First IV 50×10^6^, and one month later the IT 100×10^6^.	15	3	IV 50×10^6^ and IT 100×10^6^ for 1 time.	12m	AE; Whole transcriptome analysis	No AE occurred; The down-regulation error of inflammation-related and antigen presentation (HLA-B) genes has increased. TNF-α, TAP-1 and miR-142 were significantly downregulated.	[317]
**USA**	NCT03799718	Mar 2019-Mar 2021	MS	BM-MSCs	autologous	18	18	10	Three times 1×10^8^-1.25×10^8^ IT				38w	AE; Whole transcriptome analysis	No AE occurred; The down-regulation error of inflammation-related and antigen presentation (HLA-B) genes has increased. TNF-α, TAP-1 and miR-142 were significantly downregulated.	[321]
**USA**	NCT03691909	Sept 2018-Sept 2020	RA	AD-MSCs	autologous	15	15	1	2×10^8^ IV for one time				52w	Hematology, renal function, and liver function	It is effective to improve joint function in RA patients.	[348]
**China**	NCT01547091	Jan 2011-Dec 2018	RA	UC-MSCs	allogeneic	119	59	6	LG and UC-MSCs	60	7	UC-MSCs	1y	Anti-inflammatory factor	Reduce inflammatory cytokines and increase anti-inflammatory factors	[349]
**USA**	NCT03171194		SLE	UC-MSCs	allogeneic	6	6	0	1×10^6^/kg IV				24w	SRI 4; AE	The clinical endpoint of an SRI of 4 was achieved. No AE occurred.	[350]
**Spain**	NCT01056471		MS	AD-MSCs	autologous	30	19	6	Low dose（n=10）1×10^6^/kg, high dose（n=9）4×10^6^/kg	11	3	An equal volume of placebo was injected	12m	AE; laboratory parameters; vital signs and spirometry; EDSS; magnetic-resonance-imaging	AE occurred in only one group and was not related to the study. No changes were shown in other safety parameters.	[351]
**Korea**	NCT03174587		LN	BM-MSCs	allogeneic	7	3	0	2×10^6^/kg IV	3	0	3×10^6^/kg IV	28d	AE	No serious AE occurred.	[352]
**China**	NCT04014166	Nov 2019–Aug 2022	ITP	UC-MSCs	autologous	18	12		Dose-escalation phase:0.5×10^6^,1.0×10^6^,2.0×10^6^/kg per week for 4 times	6		Dose-expansion phase:2.0×10^6^/kg per week for 4 times	7m	AE; platelet counts; peripheral blood immunity	AE occurred. The platelet count and changes in peripheral blood immunity increased.	[359]
**Sweden**	NCT03406585	Jan 2018-Jun 2018	1 diabetes	UC-SCs	allogeneic	9	9	9	Three different doses (2.5×10^7^, 1×10^8^, 2×10^8^) of IV.				4w	Δ-change in C-peptide AUC; AE	No AE occurred; Δ-change in C-peptide AUC was not significantly decreased.	[360]
						15	10	6	2×10^8^ IV	5	2	Same amount of saline IV	12m			
**Israel**	NCT02166021		MS	BM-MSCs	autologous	48	30		1×10^6^/kg IV (n=15), 1×10^6^/kg IT(n=15)	14		Same amount of saline	12m	EDSS; T25 FW; AE; CSF	No long-term AE occurred, and EDSS and T25 FW continued to improve; Significant changes in CSF.	[361]
**China**	NCT01374854		1 diabetes	UC-MSCs	allogeneic	42	21	6	1.10±0.22×10^6^/kg and 0.61±0.26×10^10^ABM-MNCs	21	9	Same amount of saline	8y	Incidence of chronic diabetic complications; safety; islet function; metabolic control	The incidence of complications of chronic diabetes decreased, and there were statistically significant differences in pancreatic islet function decline and metabolic control.	[362]
**China**			AHA	UC-MSCs	allogeneic	18	10	5	PBMCs and UC-MSCs were co-cultured	8	4	PBMCs	3d	Cytokine level	TNF-α and IL-10 were increased, and the proportion of cell population was regulated.	[363]
**China**	ChiCTR 2100045434	2013-2019	1 diabetes	BM-MSCs	allogeneic	53	27	12	1.0×10^6^/kg repeated every three months	26	13	Standard treatment based on intensive insulin therapy	1y	Clinical remission; side effects; rum levels of HbA1c; ranges in fasting and postprandial C-peptide; daily insulin doses	The clinical remission was obvious, and the percentage change of C-peptide after meals increased significantly. No serious side effects were observed.	[364]
**China**	NCT01547091	Jan 2000-Jan 2017	RA	UC-MSCs	allogeneic	64	64	8	2×10^7^ UC-MSCsand DMARDs				3y	Serological markers; DAS 28; DAS 28	UC-MSCs combined with DMARDs are safe and effective.	[365]
**Netherlands**	NTR 4146		JIA	MSCs	allogeneic	6	6	4	2×10^6^/kg repeat IV				52w	VAS well-being, the JADAS-71, the cJADAS10	HAQ and DAS 28 decreased; The anti-CCP antibody decreased. All were statistically significant.	[366]
**Iran**	NCT03333681	July 2016-Apr 2017	RA	BM-MSCs	autologous	9			1×10^6^/kg				12y	Immunology Clinical; Accessory clinical factors	IV BM-MSCs can improve the clinical symptoms of refractory RA patients.	[367]
**China**	NCT00698191	2007-2016	AD	BM-MSCs, UC-MSCs	allogeneic	404	404	60	1×10^6^/kg				43.4±25.9m	AE	AE was acceptable.	[368]
**Korea**	NCT02221258		RA	UC-MSCs	allogeneic	9	9	7	2.5×10^7^, 5×10^7^, 1×10^8^ UC-MSCs				4w	Serum cytokines; DAS 28; AE	The levels of IL-1β, IL-6, IL-8 and TNF-α decreased; No short-term AE occurred; DAS 28 has changed.	[369]

*MS* multiple sclerosis, *EDSS* Expanded Disability Status Scale, *T25 FW* timed 25-foot walk, *ITP* Idiopathic thrombocytopenic purpura, *CSF* cerebrospinal fluid, *RA* rheumatoid arthritis, *AHA* Autoimmune hemolytic anemia, *DAS 28* 28-joint disease activity score, *AD* autoimmune disease, *HAQ* Health Assessment Questionnaire, *MSC-NPs* MSC-neural progenitors, *SLE* systemic lupus erythematosus, *ABM-MNCs* autologous bone marrow mononuclear cells, *JIA* Juvenile idiopathic arthritis, *d* day, *w* weeks, *m* month, *y* year, *USA* United States of America.

Data sources -- published literature

### Therapeutic applications in some other diseases

In addition to the treatment of these diseases, MSCs have also been widely used in diseases such as COVID-19, acute lung injury, chronic obstructive pulmonary disease (COPD), cirrhosis and CKD (Table 5) [370]. For acute lung injury, MSCs can promote the repair of alveolar epithelial cells by secreting a variety of growth factors and cytokines [371]. In COPD, MSCs reduce airway inflammation and reduce airway remodeling [372]. In the treatment of cirrhosis, MSCs can promote liver cell regeneration and reduce liver inflammation and fibrosis [373]. In CKD, MSCs can inhibit renal fibrosis and improve renal function. CKD is characterized by structural or functional abnormalities of the kidneys, typically persisting for more than three months. It can be caused by a variety of conditions, with diabetic nephropathy being the most common etiology. Diabetic nephropathy not only represents the leading cause of CKD but is also one of the most frequent complications associated with diabetes [374]. Hyperglycemia can trigger a cascade of pathophysiological events leading to glomerular and tubulointerstitial fibrosis, characterized by podocyte damage or loss and mesangial cell hypertrophy [375]. Therefore, glycemic control is paramount in the management of diabetic nephropathy. MSCs can release anti-inflammatory factors such as HGF and prostaglandin E2 via paracrine mechanisms to mitigate inflammatory responses, reduce the expression of pro-fibrotic molecules, and decrease collagen and fibronectin deposition. This ultimately ameliorates renal fibrosis and promotes the regeneration and functional recovery of islet beta cells, thereby mitigating kidney damage caused by hyperglycemia [376]. MSCs-EXO can ameliorate the symptoms of diabetic nephropathy by regulating autophagy, inhibiting fibrosis, and alleviating inflammation. For instance, BM-MSCs-EXO can induce autophagy via the mTOR signaling pathway, significantly increasing the expression of LC3 and Beclin-1 in renal tissue while reducing mTOR expression levels. This ultimately improves renal function and repairs pathological damage [377]. Furthermore, MSCs-EXO can exert an anti-apoptotic effect by inhibiting the expression of TGF-β1, maintaining the expression of tight junction proteins, and preventing the epithelial-mesenchymal transition (EMT) of renal tubular epithelial cells [378]. As research continues to advance, the application of MSCs in the treatment of an expanding range of diseases will be further optimized, offering new hope to patients.

**Table 5:** Clinical application of MSCs and MSCs-EXO in others diseases

***Nation***	***NCT***	***Duration***	***type***	***MSCs***	***source***	***Sample size***	***Experimental group***			***Control group***			***Time***	***Primary outcomes***	***Result***	***References***
							**Sample size**	**Male**	**Therapy**	**Sample size**	**Male**	**Therapy**				
*Korea*	NCT03174587		LN	BM-MSCs	allogeneic	7	3	0	2×10^6^/kg IV	3	0	3×10^6^/kg IV	28d	AE	No serious AE occurred.	[352]
*Italy*	2016-004804-77	Sept 2018-Feb 2020	INS	BM-MSCs	autologous	16	10	3	Repeated injections of 1×10^6^/kg BM-MSCs were performed at 7-day intervals	6	3	Repeated injections of 1×10^6^/kg BM-MSCs were performed at 7-day intervals.	12m	AE and clinical parameters	Reduce recurrent and immunosuppressed INS in children.	[379]
*Ireland, Italy, the United Kingdom*	NCT02585622	Mar 2018-Jan 2020	CKD	BM-MSCs	allogeneic	48	36	36	An equal distribution (8×10^7^, 1.6×10^7^, 2.4×10^7^)	12	4	Injection of placebo	18m	AE; eGFR	No AE occurred. eGFR decreased significantly.	[380]
*USA*	NCT03015623		AKI	MSCs	allogeneic	16	12	7	2.5×10^8^	5	1	The hollow fiber plasma separator but devoid of cells.	28d	AE and protein assays	Isolated MSCs are able to stimulate immune responses that trigger an accelerated phenotypic switch from tissue damage to tissue repair.	[381]
*USA*	NCT02266394	2013-2017	ARVD	AD-MSCs	autologous	37	19	11	Low dose (n=6): 1.0×10^5^/kg; Medium dose(n=7): 2.5×10^5^/kg; High dose(n=6): 5.0×10^5^/kg	18	7	Medical only treated	3y	Renal blood flow; GFR; Renal vein cytokine levels Blood pressure; tissue oxygenation	Renal blood flow increased; And GFR changes significantly; Anti-inflammatory factor increased;	[382]
*China*		2008-2013	liver cirrhosis	UC-MSCs, BM-MSCs	allogeneic	26	26	1	1×10^6^ cells/kg IV				2y	AE; Liver function indicators	No AE occurred.	[383]
*China*	ChiCTR2000030261	Feb 2020-Sept 2020	COVID-19 Pneumonia	UC-MSCs-EXO	allogeneic	7	7	2	Atomized inhalation of UC-MSCs-EXO was administered twice a day for 10 minutes each time	39		Oral treatment with conventional antiviral drugs	During hospitalization	secondary infection; allergic reactions; AE.	It will not cause acute or secondary allergic reactions, promote the absorption of lung lesions, and reduce hospital stay.	[384]
*USA*	NCT0683722		COPD	BM-MSCs	allogeneic	29	17	8	BM-MSCs therapy	12	7	Placebo therapy	2y	Lung function; exercise performance; patient reported responses; exacerbation frequency; CRP	The relevant indicators have improved significantly.	[385]
*China*	NCT01902082	Jan 2013-Apr 2013	ARDS	AD-MSCs	allogeneic	12	6	6	1×10^6^ cells/kg therapy	6	5	Placebo therapy	28d	AE; acute lung injury biomarkers	Total number of AE there was no significant difference; The level of SP-D decreased significantly.	[386]

*INS* idiopathic nephrotic syndrome, *AE* adverse events,*CKD* chronic kidney injury, *LN* lupus nephritis, *AKI* acute kidney injury, *ARVD* atherosclerotic renovascular disease, *GFR* glomerular filtration rate, *COPD* chronic obstructive pulmonary disease, *ARDS* acute respiratory distress,*CRP* C-reactive protein, *d* day,*m * month, *y* year,*USA* United States of America

Data sources -- published literature

## Conclusion and prospect

MSCs and MSCs-EXO are extensively utilized in the treatment of various common clinical conditions, particularly in CVDs, neurological disorders, ADs, and MSDs, with some studies achieving remarkable research outcomes. Although MSCs may pose carcinogenic risks such as gene mutation, genetic instability, and excessive proliferation and differentiation during treatment, and their biological activity can diminish in hypoxic microenvironments, thereby affecting therapeutic efficacy, MSCs still demonstrate significant potential as a promising treatment for regenerative medicine and tissue engineering applications.

MSCs-EXO are paracrine products of MSCs and can compensate for certain limitations of MSCs through effective intercellular communication. As a novel form of non-cellular therapy, MSCs-EXO exhibit unique advantages and characteristics in disease treatment. Although MSCs-EXO exhibit greater versatility compared to MSCs, their current clinical application is significantly more limited. The reasons for this include the following: first, the preparation and purification processes for MSCs-EXO have not yet met the standards required for clinical use; second, the storage and transportation of MSCs-EXO necessitate more stringent conditions; third, the optimal dosages of MSCs-EXO for various diseases are still under investigation. These factors collectively constrain the clinical application of MSCs-EXO.

The extensive body of existing clinical data unequivocally demonstrates the efficacy of MSCs and the utilization of MSCs-EXO in the treatment of various clinical diseases. These treatments exhibit high feasibility and safety profiles. Although studies have predominantly demonstrated positive outcomes, heterogeneity is observed in certain studies [255, 258, 261, 266, 271, 274, 295, 297, 304–306, 308, 310, 314, 324, 335, 338, 351]. After an in-depth summary of the above negative results, we found that the main reasons for these heterogeneous results included the limited number of patients included in the study and the insufficient sample size [258, 261, 297, 304–306, 308, 348, 351]. This has led to significant individual differences, such as variations in age, severity of the disease, measurement results [308], thus making it impossible to conduct in-depth multivariate analysis [297]. Furthermore, the lack of corresponding control groups for comparison [304], and a large number of unexpected placebo effects [295, 308], may had an impact on the differences in the results. In addition, due to the limited follow-up time, negative results may occur in the short term [274], and the accurate assessment of some evaluation indicators requires a longer follow-up time. In preclinical research, intervention measures can often be carried out at the early stage of disease development. However, due to the limitations of complex factors such as subject screening and experimental preparation, clinical research usually can only implement intervention measures in the late stage of the disease [324]. Intervention in the early stage of the development of certain diseases, such as the application of MSCs for treatment in the early stage after MI, may cause tissue edema in the myocardial stroma due to the recanalization of the infarct-related arteries, thereby affecting the therapeutic effect [255, 258]. In addition, the in vitro culture time of MSCs is usually long, and the long-term in vitro culture increases the risk of cell contamination [255]. It is worth noting that MSCs cells are relatively large in size, have poor motility and migration capabilities, and are prone to clumps, which may lead to vascular obstruction [255]. Meanwhile, factors such as the selection of dosage during the treatment of MSCs [266], potential cell-mediated effects [271], and biological variability [338] may lead to unsatisfactory therapeutic effects.

To summarize, the heterogeneity observed in MSCs and MSCs-EXO during clinical applications can primarily be attributed to the following factors: (1) Donor variability: The origin of MSCs and MSCs-EXO may vary due to differences in donor age, health status, gender, and genetics. (2) Isolation methods: Different isolation methods are required for various sources of MSCs. During the isolation process, contamination from other cell types, residues, or chemical enzymes may affect the growth and proliferation of target MSCs. (3) Culture conditions: Variations in culture environments, culture conditions, consumable materials, and cell passages of MSCs also contribute to heterogeneity in clinical trials. (4) Purification techniques: The use of different collection methods to obtain clinical-grade, highly purified MSCs and MSCs-EXO is a critical factor influencing the effectiveness of clinical therapy. (5) Transportation conditions: Proper transportation methods are essential to maintain the activity of MSCs and MSCs-EXO during transit. (6) Immunogenicity: Although MSCs exhibit low immunogenicity under normal conditions, disease-induced changes in the microenvironment and inflammation can lead to the upregulation of MHC II molecules on their surface, potentially causing immune rejection and thereby affecting therapeutic outcomes. (7) Injection parameters: Differences in site, frequency, type, quantity, and source of the injected MSCs and MSCs-EXO significantly impact therapeutic efficacy. (8) Disease characteristics: The type and severity of the disease also significantly influence the therapeutic efficacy of stem cells and exosomes. (9) Administration timing: The variations in the administration timing of MSCs and MSCs-EXO, as well as disparities in follow-up durations, may also contribute to differences in therapeutic efficacy. (10) Included patients’ numbers: The limited number of patients enrolled in the study, coupled with an insufficient sample size, contributed to the observed heterogeneity. (11) Placebo: The rationality of placebo selection. (12) Others: Cell-mediated effects, biological variability, injection-site edema, cell contamination, and volume changes, among other factors.

Although there are currently no standardized processes for the isolation, characterization, and administration of MSCs and MSCs-EXO, the disparities in clinical outcomes caused by these factors can be mitigated through existing technologies. Firstly, when selecting the type of MSCs, priority can be given to MSCs with stable biological characteristics (e.g., UC-MSCs), and gene-editing techniques can be employed to minimize donor-to-donor variability. However, this may give rise to certain medical ethics concerns. Secondly, stringent donor-screening criteria should be established, with a preference for young and healthy donors. An efficient and well-defined preparation process should be applied, utilizing advanced extraction techniques such as ultracentrifugation and robust quality control measures like Western blot analysis to ensure the production quality of MSCs and MSCs-EXO. Standardization of cell characterization can also be achieved by identifying specific surface markers. In the early stages of clinical application, its safety and efficacy can be validated through multi-center, large-scale clinical trials, thereby facilitating the translation of this technology from the laboratory to the clinical setting.

The development of engineered exosomes may address the challenges posed by the aforementioned factors to a certain extent. The conjugation of homing peptides, which possess cell or tissue-targeting capabilities, with MSCs-EXO significantly enhances the delivery efficiency of MSCs-EXO. This enables more precise action on specific lesion sites, thereby improving therapeutic efficacy. In addition, by conjugating homing peptides, MSCs-EXO can overcome the limitations of low survival rates and insufficient targeting capabilities observed in traditional cell therapies. Simultaneously, they avoid the risks associated with live-cell treatments, thus further enhancing their applicability in disease management [387]. However, several issues still require further resolution, including the standardized production and quality control of exosomes, as well as the binding stability between homing peptides and exosomes. MSCs-EXO can facilitate complex intercellular signal transduction processes via the transfer of bioactive factors such as proteins and nucleic acids, mitochondrial intercellular transfer, protein–protein interactions, receptor-ligand interactions, and other targeted mechanisms. They not only exhibit anti-inflammatory and immunomodulatory properties but also promote the generation and repair of tissues such as blood vessels and nerves while inhibiting cell apoptosis. During the preclinical experimental phase, MSCs-EXO demonstrated significant synergistic protective effects on the heart, nervous system, immune system, and overall body metabolism.

Therefore, further basic research is essential to elucidate the underlying treatment mechanisms and to enhance both efficacy and stability. Additionally, larger clinical trials are necessary to establish standardized protocols for the isolation, culture, preservation, and application of MSCs and MSCs-EXO. These efforts will contribute to achieving greater effectiveness and stability in the therapeutic application of MSCs and MSCs-EXO.

## Data Availability

Not applicable.

## References

[1] Ramalho-Santos M, Willenbring H. On the origin of the term “stem cell.” Cell Stem Cell. 2007;1(1):35–8. 10.1016/j.stem.2007.05.013. 18371332 10.1016/j.stem.2007.05.013

[2] Friedenstein AJ, Piatetzky S II, Petrakova KV. Osteogenesis in transplants of bone marrow cells. J Embryol Exp Morphol. 1966;16(3):381–90. 5336210

[3] Caplan AI. Mesenchymal stem cells. J Orthop Res. 1991;9(5):641–50. 10.1002/jor.1100090504. 1870029 10.1002/jor.1100090504

[4] Naji A, Eitoku M, Favier B, Deschaseaux F, Rouas-Freiss N, Suganuma N. Biological functions of mesenchymal stem cells and clinical implications. Cell Mol Life Sci. 2019;76(17):3323–48. 10.1007/s00018-019-03125-1. 31055643 10.1007/s00018-019-03125-1 PMC11105258

[5] Bonucci E. Fine structure of early cartilage calcification. J Ultrastruct Res. 1967;20(1):33–50. 10.1016/s0022-5320(67)80034-0. 4195919 10.1016/s0022-5320(67)80034-0

[6] van Niel G, D’Angelo G, Raposo G. Shedding light on the cell biology of extracellular vesicles. Nat Rev Mol Cell Biol. 2018;19(4):213–28. 10.1038/nrm.2017.125. 29339798 10.1038/nrm.2017.125

[7] Pegtel DM, Gould SJ. Exosomes. Annu Rev Biochem. 2019;88:487–514. 10.1146/annurev-biochem-013118-111902. 31220978 10.1146/annurev-biochem-013118-111902

[8] Stegmayr B, Brody I, Ronquist G. A biochemical and ultrastructural study on the endogenous protein kinase activity of secretory granule membranes of prostatic origin in human seminal plasma. J Ultrastruct Res. 1982;78(2):206–14. 10.1016/s0022-5320(82)80024-5. 6283103 10.1016/s0022-5320(82)80024-5

[9] Johnstone RM, Bianchini A, Teng K. Reticulocyte maturation and exosome release: transferrin receptor containing exosomes shows multiple plasma membrane functions. Blood. 1989;74(5):1844–51. 2790208

[10] F Z, J G, Z Z, Y Q, G W, M D, et al. Mesenchymal stem cell-derived exosome: A tumor regulator and carrier for targeted tumor therapy. Cancer letters. 2022;526:29–40. 10.1016/j.canlet.2021.11.015. 10.1016/j.canlet.2021.11.015 34800567

[11] Sato K, Meng F, Glaser S, Alpini G. Exosomes in liver pathology. J Hepatol. 2016;65(1):213–21. 10.1016/j.jhep.2016.03.004. 26988731 10.1016/j.jhep.2016.03.004 PMC4912847

[12] Ibrahim A, Marbán E. Exosomes: Fundamental Biology and Roles in Cardiovascular Physiology. Annu Rev Physiol. 2016;78:67–83. 10.1146/annurev-physiol-021115-104929. 26667071 10.1146/annurev-physiol-021115-104929 PMC5425157

[13] Williams T, Salmanian G, Burns M, Maldonado V, Smith E, Porter RM, et al. Versatility of mesenchymal stem cell-derived extracellular vesicles in tissue repair and regenerative applications. Biochimie. 2023;207:33–48. 10.1016/j.biochi.2022.11.011. 36427681 10.1016/j.biochi.2022.11.011

[14] Missoum A. Recent Updates on Mesenchymal Stem Cell Based Therapy for Acute Renal Failure. Curr Urol. 2020;13(4):189–99. 10.1159/000499272. 31998051 10.1159/000499272 PMC6976998

[15] Friedenstein AJ, Gorskaja JF, Kulagina NN. Fibroblast precursors in normal and irradiated mouse hematopoietic organs. Exp Hematol. 1976;4(5):267–74. 976387

[16] Miao C, Lei M, Hu W, Han S, Wang Q. A brief review: the therapeutic potential of bone marrow mesenchymal stem cells in myocardial infarction. Stem Cell Res Ther. 2017;8(1):242. 10.1186/s13287-017-0697-9. 29096705 10.1186/s13287-017-0697-9 PMC5667518

[17] Cakouros D, Gronthos S. Epigenetic Regulation of Bone Marrow Stem Cell Aging: Revealing Epigenetic Signatures associated with Hematopoietic and Mesenchymal Stem Cell Aging. Aging Dis. 2019;10(1):174–89. 10.14336/ad.2017.1213. 30705777 10.14336/AD.2017.1213 PMC6345334

[18] Zou XF, Zhang BZ, Qian WW, Cheng FM. Bone marrow mesenchymal stem cells in treatment of peripheral nerve injury. World J Stem Cells. 2024;16(8):799–810. 10.4252/wjsc.v16.i8.799. 39219723 10.4252/wjsc.v16.i8.799 PMC11362854

[19] Stolzing A, Jones E, McGonagle D, Scutt A. Age-related changes in human bone marrow-derived mesenchymal stem cells: consequences for cell therapies. Mech Ageing Dev. 2008;129(3):163–73. 10.1016/j.mad.2007.12.002. 18241911 10.1016/j.mad.2007.12.002

[20] Lee JS, Kim SK, Cha JK, Jung BJ, Choi SB, Choi EY, et al. Novel Technique for Isolating Human Bone Marrow Stem Cells Using Hyaluronic Acid Hydrogel. Tissue Eng Part C Methods. 2016;22(10):941–51. 10.1089/ten.TEC.2016.0214. 27609497 10.1089/ten.TEC.2016.0214

[21] Torre ML, Lucarelli E, Guidi S, Ferrari M, Alessandri G, De Girolamo L, et al. Ex vivo expanded mesenchymal stromal cell minimal quality requirements for clinical application. Stem Cells Dev. 2015;24(6):677–85. 10.1089/scd.2014.0299. 25517941 10.1089/scd.2014.0299

[22] Mareschi K, Rustichelli D, Calabrese R, Gunetti M, Sanavio F, Castiglia S, et al. Multipotent mesenchymal stromal stem cell expansion by plating whole bone marrow at a low cellular density: a more advantageous method for clinical use. Stem Cells Int. 2012;2012: 920581. 10.1155/2012/920581. 23715383 10.1155/2012/920581 PMC3195433

[23] Tarte K, Gaillard J, Lataillade JJ, Fouillard L, Becker M, Mossafa H, et al. Clinical-grade production of human mesenchymal stromal cells: occurrence of aneuploidy without transformation. Blood. 2010;115(8):1549–53. 10.1182/blood-2009-05-219907. 20032501 10.1182/blood-2009-05-219907

[24] Castiglia S, Mareschi K, Labanca L, Lucania G, Leone M, Sanavio F, et al. Inactivated human platelet lysate with psoralen: a new perspective for mesenchymal stromal cell production in Good Manufacturing Practice conditions. Cytotherapy. 2014;16(6):750–63. 10.1016/j.jcyt.2013.12.008. 24529555 10.1016/j.jcyt.2013.12.008 PMC7185570

[25] Zhang X, Kong Y, Sun Y, Qian Z, Gao C, Shi X, et al. Bone marrow mesenchymal stem cells conditioned medium protects VSC4.1 cells against 2,5-hexanedione-induced autophagy via NGF-PI3K/Akt/mTOR signaling pathway. Brain Res. 2018;1696:1–9. 10.1016/j.brainres.2018.04.028. 10.1016/j.brainres.2018.04.028 29705604

[26] Rezaei N, Bojnordi MN, Ghasemi HH. Differentiation of bone marrow stromal stem cells seeded on silk scaffold to mature oligodendrocyte using cerebrospinal fluid. J Chem Neuroanat. 2020;106: 101790. 10.1016/j.jchemneu.2020.101790. 32278022 10.1016/j.jchemneu.2020.101790

[27] Cheng MT, Liu CL, Chen TH, Lee OK. Optimization of culture conditions for stem cells derived from human anterior cruciate ligament and bone marrow. Cell Transplant. 2014;23(7):791–803. 10.3727/096368912x666430. 23582177 10.3727/096368912X666430

[28] Meuleman N, Tondreau T, Delforge A, Dejeneffe M, Massy M, Libertalis M, et al. Human marrow mesenchymal stem cell culture: serum-free medium allows better expansion than classical alpha-MEM medium. Eur J Haematol. 2006;76(4):309–16. 10.1111/j.1600-0609.2005.00611.x. 16519702 10.1111/j.1600-0609.2005.00611.x

[29] Zuk PA, Zhu M, Ashjian P, De Ugarte DA, Huang JI, Mizuno H, et al. Human adipose tissue is a source of multipotent stem cells. Mol Biol Cell. 2002;13(12):4279–95. 10.1091/mbc.e02-02-0105. 12475952 10.1091/mbc.E02-02-0105 PMC138633

[30] Bourin P, Bunnell BA, Casteilla L, Dominici M, Katz AJ, March KL, et al. Stromal cells from the adipose tissue-derived stromal vascular fraction and culture expanded adipose tissue-derived stromal/stem cells: a joint statement of the International Federation for Adipose Therapeutics and Science (IFATS) and the International Society for Cellular Therapy (ISCT). Cytotherapy. 2013;15(6):641–8. 10.1016/j.jcyt.2013.02.006. 23570660 10.1016/j.jcyt.2013.02.006 PMC3979435

[31] Yoshimura K, Suga H, Eto H. Adipose-derived stem/progenitor cells: roles in adipose tissue remodeling and potential use for soft tissue augmentation. Regen Med. 2009;4(2):265–73. 10.2217/17460751.4.2.265. 19317645 10.2217/17460751.4.2.265

[32] Bernardo ME, Locatelli F, Fibbe WE. Mesenchymal stromal cells. Ann N Y Acad Sci. 2009;1176:101–17. 10.1111/j.1749-6632.2009.04607.x. 19796238 10.1111/j.1749-6632.2009.04607.x

[33] Kern S, Eichler H, Stoeve J, Klüter H, Bieback K. Comparative analysis of mesenchymal stem cells from bone marrow, umbilical cord blood, or adipose tissue. Stem Cells. 2006;24(5):1294–301. 10.1634/stemcells.2005-0342. 16410387 10.1634/stemcells.2005-0342

[34] Izadpanah R, Trygg C, Patel B, Kriedt C, Dufour J, Gimble JM, et al. Biologic properties of mesenchymal stem cells derived from bone marrow and adipose tissue. J Cell Biochem. 2006;99(5):1285–97. 10.1002/jcb.20904. 16795045 10.1002/jcb.20904 PMC4048742

[35] De Francesco F, Ricci G, D’Andrea F, Nicoletti GF, Ferraro GA. Human Adipose Stem Cells: From Bench to Bedside. Tissue Eng Part B Rev. 2015;21(6):572–84. 10.1089/ten.TEB.2014.0608. 25953464 10.1089/ten.TEB.2014.0608

[36] Kd M, Ai I, Kt E, Wh M. Isolation, culture and characterisation of fibroblast-like cells derived from the Wharton’s jelly portion of human umbilical cord. Biochem Soc Trans. 1991;19(1):29S. 10.1042/bst019029s. 1709890 10.1042/bst019029s

[37] Chetty S, Yarani R, Swaminathan G, Primavera R, Regmi S, Rai S, et al. Umbilical cord mesenchymal stromal cells-from bench to bedside. Front Cell Dev Biol. 2022;10:1006295. 10.3389/fcell.2022.1006295. 36313578 10.3389/fcell.2022.1006295 PMC9597686

[38] Li X, Bai J, Ji X, Li R, Xuan Y, Wang Y. Comprehensive characterization of four different populations of human mesenchymal stem cells as regards their immune properties, proliferation and differentiation. Int J Mol Med. 2014;34(3):695–704. 10.3892/ijmm.2014.1821. 24970492 10.3892/ijmm.2014.1821 PMC4121354

[39] Chen Y, Shen H, Ding Y, Yu Y, Shao L, Shen Z. The application of umbilical cord-derived MSCs in cardiovascular diseases. J Cell Mol Med. 2021;25(17):8103–14. 10.1111/jcmm.16830. 34378345 10.1111/jcmm.16830 PMC8419197

[40] Bongso A, Fong CY. The therapeutic potential, challenges and future clinical directions of stem cells from the Wharton’s jelly of the human umbilical cord. Stem Cell Rev Rep. 2013;9(2):226–40. 10.1007/s12015-012-9418-z. 23233233 10.1007/s12015-012-9418-z

[41] Gao S, Jin Y, Ma J, Wang J, Wang J, Shao Z, et al. Preclinical study of human umbilical cord mesenchymal stem cell sheets for the recovery of ischemic heart tissue. Stem Cell Res Ther. 2022;13(1):252. 10.1186/s13287-022-02919-8. 35690871 10.1186/s13287-022-02919-8 PMC9188245

[42] Margossian T, Reppel L, Makdissy N, Stoltz JF, Bensoussan D, Huselstein C. Mesenchymal stem cells derived from Wharton’s jelly: comparative phenotype analysis between tissue and in vitro expansion. Biomed Mater Eng. 2012;22(4):243–54. 10.3233/bme-2012-0714. 22785368 10.3233/BME-2012-0714

[43] Yoon JH, Roh EY, Shin S, Jung NH, Song EY, Chang JY, et al. Comparison of explant-derived and enzymatic digestion-derived MSCs and the growth factors from Wharton’s jelly. Biomed Res Int. 2013;2013: 428726. 10.1155/2013/428726. 23653895 10.1155/2013/428726 PMC3638666

[44] Semenova E, Grudniak MP, Machaj EK, Bocian K, Chroscinska-Krawczyk M, Trochonowicz M, et al. Mesenchymal Stromal Cells from Different Parts of Umbilical Cord: Approach to Comparison & Characteristics. Stem Cell Rev Rep. 2021;17(5):1780–95. 10.1007/s12015-021-10157-3. 33860454 10.1007/s12015-021-10157-3 PMC8553697

[45] Feng X, Liu J, Xu Y, Zhu J, Chen W, Feng B, et al. Molecular mechanism underlying the difference in proliferation between placenta-derived and umbilical cord-derived mesenchymal stem cells. J Cell Physiol. 2020;235(10):6779–93. 10.1002/jcp.29572. 31990045 10.1002/jcp.29572

[46] Chia WK, Cheah FC, Abdul Aziz NH, Kampan NC, Shuib S, Khong TY, et al. A Review of Placenta and Umbilical Cord-Derived Stem Cells and the Immunomodulatory Basis of Their Therapeutic Potential in Bronchopulmonary Dysplasia. Front Pediatr. 2021;9: 615508. 10.3389/fped.2021.615508. 33791258 10.3389/fped.2021.615508 PMC8006350

[47] Aramburú Junior JS, Eilers Treichel TL, Lemos Pinto Filho ST, Gehrke SA, Machado AK, Cadoná FC, et al. DNA damage in dental pulp mesenchymal stem cells: An in vitro study. Vet Res Forum. 2018;9(4):293–9. 10.30466/vrf.2018.33083. 10.30466/vrf.2018.33083 PMC6346493 30713606

[48] Stanko P, Altanerova U, Jakubechova J, Repiska V, Altaner C. Dental Mesenchymal Stem/Stromal Cells and Their Exosomes. Stem Cells Int. 2018;2018:8973613. 10.1155/2018/8973613. 29760738 10.1155/2018/8973613 PMC5924966

[49] Nakamura S, Yamada Y, Katagiri W, Sugito T, Ito K, Ueda M. Stem cell proliferation pathways comparison between human exfoliated deciduous teeth and dental pulp stem cells by gene expression profile from promising dental pulp. J Endod. 2009;35(11):1536–42. 10.1016/j.joen.2009.07.024. 19840643 10.1016/j.joen.2009.07.024

[50] Lee YC, Chan YH, Hsieh SC, Lew WZ, Feng SW. Comparing the Osteogenic Potentials and Bone Regeneration Capacities of Bone Marrow and Dental Pulp Mesenchymal Stem Cells in a Rabbit Calvarial Bone Defect Model. Int J Mol Sci. 2019;20(20). 10.3390/ijms20205015. 10.3390/ijms20205015 PMC6834129 31658685

[51] M D, JC F, M P, E P-G, A M, F M-G, et al. Phenotypic Identification of Dental Pulp Mesenchymal Stem/Stromal Cells Subpopulations with Multiparametric Flow Cytometry. Methods in molecular biology (Clifton, NJ). 2019;1922:77–90. 10.1007/978-1-4939-9012-2_8. 10.1007/978-1-4939-9012-2_8 30838566

[52] KB G, DS S, AF E, DN S, AC dA, RR DS, et al. Conditioned Medium of Bone Marrow-Derived Mesenchymal Stromal Cells as a Therapeutic Approach to Neuropathic Pain: A Preclinical Evaluation. Stem cells international. 2018;2018:8179013. 10.1155/2018/8179013. 10.1155/2018/8179013 PMC5831939 29535781

[53] Sekiya I, Muneta T, Horie M, Koga H. Arthroscopic Transplantation of Synovial Stem Cells Improves Clinical Outcomes in Knees With Cartilage Defects. Clin Orthop Relat Res. 2015;473(7):2316–26. 10.1007/s11999-015-4324-8. 25925939 10.1007/s11999-015-4324-8 PMC4457765

[54] Guo L, Qin W, Cai Z, Su X. Hybrid Clustering Algorithm Based on Improved Density Peak Clustering. Appl Sci. 2024;14(2):715.

[55] N K, S N, M Y, T O. Properties of Dental Pulp-derived Mesenchymal Stem Cells and the Effects of Culture Conditions. Journal of endodontics. 2017;43(9S):S31-S4. 10.1016/j.joen.2017.06.004. 10.1016/j.joen.2017.06.004 28781092

[56] Noda S, Kawashima N, Yamamoto M, Hashimoto K, Nara K, Sekiya I, et al. Effect of cell culture density on dental pulp-derived mesenchymal stem cells with reference to osteogenic differentiation. Sci Rep. 2019;9(1):5430. 10.1038/s41598-019-41741-w. 30931957 10.1038/s41598-019-41741-w PMC6443725

[57] Shen WC, Lai YC, Li LH, Liao K, Lai HC, Kao SY, et al. Methylation and PTEN activation in dental pulp mesenchymal stem cells promotes osteogenesis and reduces oncogenesis. Nat Commun. 2019;10(1):2226. 10.1038/s41467-019-10197-x. 31110221 10.1038/s41467-019-10197-x PMC6527698

[58] Eiro N, Fraile M, Schneider J, Vizoso FJ. Non Pregnant Human Uterus as Source of Mesenchymal Stem Cells. Curr Stem Cell Res Ther. 2018;13(6):423–31. 10.2174/1381612824666180426120459. 29701160 10.2174/1381612824666180426120459

[59] Hass R, Kasper C, Böhm S, Jacobs R. Different populations and sources of human mesenchymal stem cells (MSC): A comparison of adult and neonatal tissue-derived MSC. Cell Commun Signal. 2011;9:12. 10.1186/1478-811x-9-12. 21569606 10.1186/1478-811X-9-12 PMC3117820

[60] Nili E, Li FJ, Dawson RA, Lau C, McEwan B, Barnett NL, et al. The Impact of Limbal Mesenchymal Stromal Cells on Healing of Acute Ocular Surface Wounds Is Improved by Pre-cultivation and Implantation in the Presence of Limbal Epithelial Cells. Cell Transplant. 2019;28(9–10):1257–70. 10.1177/0963689719858577. 31208228 10.1177/0963689719858577 PMC6767890

[61] Duan W, Wang H, Wang Z, Ren Z, Li X, He F, et al. Multi-functional composite dressings with sustained release of MSC-SLP and anti-adhesion property for accelerating wound healing. Mater Today Bio. 2024;25: 100979. 10.1016/j.mtbio.2024.100979. 38375318 10.1016/j.mtbio.2024.100979 PMC10875241

[62] Costela-Ruiz VJ, Melguizo-Rodríguez L, Bellotti C, Illescas-Montes R, Stanco D, Arciola CR, et al. Different Sources of Mesenchymal Stem Cells for Tissue Regeneration: A Guide to Identifying the Most Favorable One in Orthopedics and Dentistry Applications. Int J Mol Sci. 2022;23(11). 10.3390/ijms23116356. 10.3390/ijms23116356 PMC9181542 35683035

[63] Corselli M, Chen CW, Sun B, Yap S, Rubin JP, Péault B. The tunica adventitia of human arteries and veins as a source of mesenchymal stem cells. Stem Cells Dev. 2012;21(8):1299–308. 10.1089/scd.2011.0200. 21861688 10.1089/scd.2011.0200 PMC3353742

[64] Garikipati VNS, Singh SP, Mohanram Y, Gupta AK, Kapoor D, Nityanand S. Isolation and characterization of mesenchymal stem cells from human fetus heart. PLoS ONE. 2018;13(2): e0192244. 10.1371/journal.pone.0192244. 29420637 10.1371/journal.pone.0192244 PMC5805293

[65] Théry C, Witwer KW, Aikawa E, Alcaraz MJ, Anderson JD, Andriantsitohaina R, et al. Minimal information for studies of extracellular vesicles 2018 (MISEV2018): a position statement of the International Society for Extracellular Vesicles and update of the MISEV2014 guidelines. J Extracell Vesicles. 2018;7(1):1535750. 10.1080/20013078.2018.1535750. 30637094 10.1080/20013078.2018.1535750 PMC6322352

[66] He C, Zheng S, Luo Y, Wang B. Exosome Theranostics: Biology and Translational Medicine. Theranostics. 2018;8(1):237–55. 10.7150/thno.21945. 29290805 10.7150/thno.21945 PMC5743472

[67] Yang D, Zhang W, Zhang H, Zhang F, Chen L, Ma L, et al. Progress, opportunity, and perspective on exosome isolation - efforts for efficient exosome-based theranostics. Theranostics. 2020;10(8):3684–707. 10.7150/thno.41580. 32206116 10.7150/thno.41580 PMC7069071

[68] Théry C, Amigorena S, Raposo G, Clayton A. Isolation and characterization of exosomes from cell culture supernatants and biological fluids. Curr Protoc Cell Biol. 2006;Chapter 3:Unit 3.22. 10.1002/0471143030.cb0322s30. 10.1002/0471143030.cb0322s30 18228490

[69] Ansari FJ, Tafti HA, Amanzadeh A, Rabbani S, Shokrgozar MA, Heidari R, et al. Comparison of the efficiency of ultrafiltration, precipitation, and ultracentrifugation methods for exosome isolation. Biochem Biophys Rep. 2024;38: 101668. 10.1016/j.bbrep.2024.101668. 38405663 10.1016/j.bbrep.2024.101668 PMC10885727

[70] Soares Martins T, Catita J, Martins Rosa I, O ABdCES, Henriques AG. Exosome isolation from distinct biofluids using precipitation and column-based approaches. PLoS One. 2018;13(6):e0198820. 10.1371/journal.pone.0198820. 10.1371/journal.pone.0198820 PMC5995457 29889903

[71] Hartjes TA, Mytnyk S, Jenster GW, van Steijn V, van Royen ME. Extracellular Vesicle Quantification and Characterization: Common Methods and Emerging Approaches. Bioengineering (Basel). 2019;6(1). 10.3390/bioengineering6010007. 10.3390/bioengineering6010007 PMC6466085 30654439

[72] Rohde E, Pachler K, Gimona M. Manufacturing and characterization of extracellular vesicles from umbilical cord-derived mesenchymal stromal cells for clinical testing. Cytotherapy. 2019;21(6):581–92. 10.1016/j.jcyt.2018.12.006. 30979664 10.1016/j.jcyt.2018.12.006

[73] Stawarska A, Bamburowicz-Klimkowska M, Runden-Pran E, Dusinska M, Cimpan MR, Rios-Mondragon I, et al. Extracellular Vesicles as Next-Generation Diagnostics and Advanced Therapy Medicinal Products. Int J Mol Sci. 2024;25(12). 10.3390/ijms25126533. 10.3390/ijms25126533 PMC11204223 38928240

[74] Wang CK, Tsai TH, Lee CH. Regulation of exosomes as biologic medicines: Regulatory challenges faced in exosome development and manufacturing processes. Clin Transl Sci. 2024;17(8): e13904. 10.1111/cts.13904. 39115257 10.1111/cts.13904 PMC11307316

[75] Hessvik NP, Llorente A. Current knowledge on exosome biogenesis and release. Cell Mol Life Sci. 2018;75(2):193–208. 10.1007/s00018-017-2595-9. 28733901 10.1007/s00018-017-2595-9 PMC5756260

[76] Ala M. The beneficial effects of mesenchymal stem cells and their exosomes on myocardial infarction and critical considerations for enhancing their efficacy. Ageing Res Rev. 2023;89: 101980. 10.1016/j.arr.2023.101980. 37302757 10.1016/j.arr.2023.101980

[77] Shetgaonkar GG, Marques SM, CEM DC, Vibhavari RJA, Kumar L, Shirodkar RK. Exosomes as cell-derivative carriers in the diagnosis and treatment of central nervous system diseases. Drug Deliv Transl Res. 2022;12(5):1047–79. 10.1007/s13346-021-01026-0. 10.1007/s13346-021-01026-0 PMC8942947 34365576

[78] Yu X, Odenthal M, Fries JW. Exosomes as miRNA Carriers: Formation-Function-Future. Int J Mol Sci. 2016;17(12). 10.3390/ijms17122028. 10.3390/ijms17122028 PMC5187828 27918449

[79] Sheng ZH, Cai Q. Mitochondrial transport in neurons: impact on synaptic homeostasis and neurodegeneration. Nat Rev Neurosci. 2012;13(2):77–93. 10.1038/nrn3156. 22218207 10.1038/nrn3156 PMC4962561

[80] Hayakawa K, Esposito E, Wang X, Terasaki Y, Liu Y, Xing C, et al. Transfer of mitochondria from astrocytes to neurons after stroke. Nature. 2016;535(7613):551–5. 10.1038/nature18928. 27466127 10.1038/nature18928 PMC4968589

[81] Morrison TJ, Jackson MV, Cunningham EK, Kissenpfennig A, McAuley DF, O’Kane CM, et al. Mesenchymal Stromal Cells Modulate Macrophages in Clinically Relevant Lung Injury Models by Extracellular Vesicle Mitochondrial Transfer. Am J Respir Crit Care Med. 2017;196(10):1275–86. 10.1164/rccm.201701-0170OC. 28598224 10.1164/rccm.201701-0170OC PMC5694830

[82] Qin Y, Jiang X, Yang Q, Zhao J, Zhou Q, Zhou Y. The Functions, Methods, and Mobility of Mitochondrial Transfer Between Cells. Front Oncol. 2021;11: 672781. 10.3389/fonc.2021.672781. 34041035 10.3389/fonc.2021.672781 PMC8141658

[83] Yang J, Liu L, Oda Y, Wada K, Ago M, Matsuda S, et al. Extracellular Vesicles and Cx43-Gap Junction Channels Are the Main Routes for Mitochondrial Transfer from Ultra-Purified Mesenchymal Stem Cells, RECs. Int J Mol Sci. 2023;24(12). 10.3390/ijms241210294. 10.3390/ijms241210294 PMC10299354 37373439

[84] Horibe S, Tanahashi T, Kawauchi S, Murakami Y, Rikitake Y. Mechanism of recipient cell-dependent differences in exosome uptake. BMC Cancer. 2018;18(1):47. 10.1186/s12885-017-3958-1. 29306323 10.1186/s12885-017-3958-1 PMC5756423

[85] Willms E, Cabañas C, Mäger I, Wood MJA, Vader P. Extracellular Vesicle Heterogeneity: Subpopulations, Isolation Techniques, and Diverse Functions in Cancer Progression. Front Immunol. 2018;9:738. 10.3389/fimmu.2018.00738. 29760691 10.3389/fimmu.2018.00738 PMC5936763

[86] Hoshino A, Costa-Silva B, Shen TL, Rodrigues G, Hashimoto A, Tesic Mark M, et al. Tumour exosome integrins determine organotropic metastasis. Nature. 2015;527(7578):329–35. 10.1038/nature15756. 26524530 10.1038/nature15756 PMC4788391

[87] Sorgen PL, Trease AJ, Spagnol G, Delmar M, Nielsen MS. Protein⁻Protein Interactions with Connexin 43: Regulation and Function. International journal of molecular sciences. 2018;19(5). 10.3390/ijms19051428. 10.3390/ijms19051428 PMC5983787 29748463

[88] Shimaoka M, Kawamoto E, Gaowa A, Okamoto T, Park EJ. Connexins and Integrins in Exosomes. Cancers. 2019;11(1). 10.3390/cancers11010106. 10.3390/cancers11010106 PMC6356207 30658425

[89] Mathieu M, Névo N, Jouve M, Valenzuela JI, Maurin M, Verweij FJ, et al. Specificities of exosome versus small ectosome secretion revealed by live intracellular tracking of CD63 and CD9. Nat Commun. 2021;12(1):4389. 10.1038/s41467-021-24384-2. 34282141 10.1038/s41467-021-24384-2 PMC8289845

[90] Fan Y, Pionneau C, Cocozza F, Boëlle PY, Chardonnet S, Charrin S, et al. Differential proteomics argues against a general role for CD9, CD81 or CD63 in the sorting of proteins into extracellular vesicles. J Extracell Vesicles. 2023;12(8): e12352. 10.1002/jev2.12352. 37525398 10.1002/jev2.12352 PMC10390663

[91] Ai Y, Guo C, Garcia-Contreras M, Sánchez BL, Saftics A, Shodubi O, et al. Endocytosis blocks the vesicular secretion of exosome marker proteins. Sci Adv. 2024;10(19):eadi9156. 10.1126/sciadv.adi9156. 10.1126/sciadv.adi9156 PMC11078179 38718108

[92] Wiklander OP, Nordin JZ, O’Loughlin A, Gustafsson Y, Corso G, Mäger I, et al. Extracellular vesicle in vivo biodistribution is determined by cell source, route of administration and targeting. J Extracell Vesicles. 2015;4:26316. 10.3402/jev.v4.26316. 25899407 10.3402/jev.v4.26316 PMC4405624

[93] Watson DC, Bayik D, Srivatsan A, Bergamaschi C, Valentin A, Niu G, et al. Efficient production and enhanced tumor delivery of engineered extracellular vesicles. Biomaterials. 2016;105:195–205. 10.1016/j.biomaterials.2016.07.003. 27522254 10.1016/j.biomaterials.2016.07.003 PMC7156278

[94] Tan F, Li X, Wang Z, Li J, Shahzad K, Zheng J. Clinical applications of stem cell-derived exosomes. Signal Transduct Target Ther. 2024;9(1):17. 10.1038/s41392-023-01704-0. 38212307 10.1038/s41392-023-01704-0 PMC10784577

[95] T LR, Sánchez-Abarca LI, Muntión S, Preciado S, Puig N, López-Ruano G, et al. MSC surface markers (CD44, CD73, and CD90) can identify human MSC-derived extracellular vesicles by conventional flow cytometry. Cell Commun Signal. 2016;14:2. 10.1186/s12964-015-0124-8. 10.1186/s12964-015-0124-8 PMC4709865 26754424

[96] Wang S, Li L, Liu T, Jiang W, Hu X. miR-19a/19b-loaded exosomes in combination with mesenchymal stem cell transplantation in a preclinical model of myocardial infarction. Regen Med. 2020;15(6):1749–59. 10.2217/rme-2019-0136. 32772806 10.2217/rme-2019-0136

[97] Wang L, Jia Q, Xinnong C, Xie Y, Yang Y, Zhang A, et al. Role of cardiac progenitor cell-derived exosome-mediated microRNA-210 in cardiovascular disease. J Cell Mol Med. 2019;23(11):7124–31. 10.1111/jcmm.14562. 31557390 10.1111/jcmm.14562 PMC6815838

[98] Pan Q, Kuang X, Cai S, Wang X, Du D, Wang J, et al. miR-132-3p priming enhances the effects of mesenchymal stromal cell-derived exosomes on ameliorating brain ischemic injury. Stem Cell Res Ther. 2020;11(1):260. 10.1186/s13287-020-01761-0. 32600449 10.1186/s13287-020-01761-0 PMC7322840

[99] Zhai K, Deng L, Wu Y, Li H, Zhou J, Shi Y, et al. Extracellular vesicle-derived miR-146a as a novel crosstalk mechanism for high-fat induced atherosclerosis by targeting SMAD4. J Adv Res. 2024. 10.1016/j.jare.2024.08.012.39127099 10.1016/j.jare.2024.08.012

[100] Poulet C, Njock MS, Moermans C, Louis E, Louis R, Malaise M, et al. Exosomal Long Non-Coding RNAs in Lung Diseases. International journal of molecular sciences. 2020;21(10). 10.3390/ijms21103580. 10.3390/ijms21103580 PMC7279016 32438606

[101] Han Y, Ren J, Bai Y, Pei X, Han Y. Exosomes from hypoxia-treated human adipose-derived mesenchymal stem cells enhance angiogenesis through VEGF/VEGF-R. Int J Biochem Cell Biol. 2019;109:59–68. 10.1016/j.biocel.2019.01.017. 30710751 10.1016/j.biocel.2019.01.017

[102] Divband S, Tasharrofi N, Abroun S, Soufi ZM. Human Umbilical Cord Mesenchymal Stem Cells-Derived Small Extracellular Vesicles Can Be Considered as Cell-Free Therapeutics for Angiogenesis Promotion. Cell J. 2022;24(11):689–96. 10.22074/cellj.2022.8275. 36377219 10.22074/cellj.2022.8275 PMC9663965

[103] Zhou H, Wu Y, Xue J, Yu L. Ameliorative effects of HGF-overexpressed exosomes derived from ADMSCs on oxidative stress in hepatic fibrosis. Histology and histopathology. 2024:18816. 10.14670/hh-18-816. 10.14670/HH-18-816 39397450

[104] Liu Y, Wang M, Yu Y, Li C, Zhang C. Advances in the study of exosomes derived from mesenchymal stem cells and cardiac cells for the treatment of myocardial infarction. Cell Commun Signal. 2023;21(1):202. 10.1186/s12964-023-01227-9. 37580705 10.1186/s12964-023-01227-9 PMC10424417

[105] Wang ZG, He ZY, Liang S, Yang Q, Cheng P, Chen AM. Comprehensive proteomic analysis of exosomes derived from human bone marrow, adipose tissue, and umbilical cord mesenchymal stem cells. Stem Cell Res Ther. 2020;11(1):511. 10.1186/s13287-020-02032-8. 33246507 10.1186/s13287-020-02032-8 PMC7694919

[106] Li Q, Xu Y, Lv K, Wang Y, Zhong Z, Xiao C, et al. Small extracellular vesicles containing miR-486–5p promote angiogenesis after myocardial infarction in mice and nonhuman primates. Sci Transl Med. 2021;13(584). 10.1126/scitranslmed.abb0202. 10.1126/scitranslmed.abb0202 33692129

[107] Shao M, Xu Q, Wu Z, Chen Y, Shu Y, Cao X, et al. Exosomes derived from human umbilical cord mesenchymal stem cells ameliorate IL-6-induced acute liver injury through miR-455-3p. Stem Cell Res Ther. 2020;11(1):37. 10.1186/s13287-020-1550-0. 31973730 10.1186/s13287-020-1550-0 PMC6979401

[108] Dong L, Pu Y, Zhang L, Qi Q, Xu L, Li W, et al. Human umbilical cord mesenchymal stem cell-derived extracellular vesicles promote lung adenocarcinoma growth by transferring miR-410. Cell Death Dis. 2018;9(2):218. 10.1038/s41419-018-0323-5. 29440630 10.1038/s41419-018-0323-5 PMC5833395

[109] Yoo JK, Kim CH, Jung HY, Lee DR, Kim JK. Discovery and characterization of miRNA during cellular senescence in bone marrow-derived human mesenchymal stem cells. Exp Gerontol. 2014;58:139–45. 10.1016/j.exger.2014.07.020. 25087724 10.1016/j.exger.2014.07.020

[110] Lu GM, Rong YX, Liang ZJ, Hunag DL, Ma YF, Luo ZZ, et al. Multiomics global landscape of stemness-related gene clusters in adipose-derived mesenchymal stem cells. Stem Cell Res Ther. 2020;11(1):310. 10.1186/s13287-020-01823-3. 32698873 10.1186/s13287-020-01823-3 PMC7374825

[111] Kusuma GD, Carthew J, Lim R, Frith JE. Effect of the Microenvironment on Mesenchymal Stem Cell Paracrine Signaling: Opportunities to Engineer the Therapeutic Effect. Stem Cells Dev. 2017;26(9):617–31. 10.1089/scd.2016.0349. 28186467 10.1089/scd.2016.0349

[112] Pincela Lins PM, Pirlet E, Szymonik M, Bronckaers A, Nelissen I. Manufacture of extracellular vesicles derived from mesenchymal stromal cells. Trends Biotechnol. 2023;41(7):965–81. 10.1016/j.tibtech.2023.01.003. 36750391 10.1016/j.tibtech.2023.01.003

[113] L PK, Kandoi S, Misra R, S V, K R, Verma RS. The mesenchymal stem cell secretome: A new paradigm towards cell-free therapeutic mode in regenerative medicine. Cytokine Growth Factor Rev. 2019;46:1–9. 10.1016/j.cytogfr.2019.04.002. 10.1016/j.cytogfr.2019.04.002 30954374

[114] Chang C, Yan J, Yao Z, Zhang C, Li X, Mao HQ. Effects of Mesenchymal Stem Cell-Derived Paracrine Signals and Their Delivery Strategies. Adv Healthc Mater. 2021;10(7): e2001689. 10.1002/adhm.202001689. 33433956 10.1002/adhm.202001689 PMC7995150

[115] Han Y, Yang J, Fang J, Zhou Y, Candi E, Wang J, et al. The secretion profile of mesenchymal stem cells and potential applications in treating human diseases. Signal Transduct Target Ther. 2022;7(1):92. 10.1038/s41392-022-00932-0. 35314676 10.1038/s41392-022-00932-0 PMC8935608

[116] Yamaguchi S, Shibata R, Yamamoto N, Nishikawa M, Hibi H, Tanigawa T, et al. Dental pulp-derived stem cell conditioned medium reduces cardiac injury following ischemia-reperfusion. Sci Rep. 2015;5:16295. 10.1038/srep16295. 26542315 10.1038/srep16295 PMC4635346

[117] Lee TL, Lai TC, Lin SR, Lin SW, Chen YC, Pu CM, et al. Conditioned medium from adipose-derived stem cells attenuates ischemia/reperfusion-induced cardiac injury through the microRNA-221/222/PUMA/ETS-1 pathway. Theranostics. 2021;11(7):3131–49. 10.7150/thno.52677. 33537078 10.7150/thno.52677 PMC7847683

[118] Baldassarro VA, Perut F, Cescatti M, Pinto V, Fazio N, Alastra G, et al. Intra-individual variability in the neuroprotective and promyelinating properties of conditioned culture medium obtained from human adipose mesenchymal stromal cells. Stem Cell Res Ther. 2023;14(1):128. 10.1186/s13287-023-03344-1. 37170115 10.1186/s13287-023-03344-1 PMC10173531

[119] Yang Y, Lee EH, Yang Z. Hypoxia-Conditioned Mesenchymal Stem Cells in Tissue Regeneration Application. Tissue Eng Part B Rev. 2022;28(5):966–77. 10.1089/ten.TEB.2021.0145. 34569290 10.1089/ten.TEB.2021.0145

[120] Kim HW, Haider HK, Jiang S, Ashraf M. Ischemic preconditioning augments survival of stem cells via miR-210 expression by targeting caspase-8-associated protein 2. J Biol Chem. 2009;284(48):33161–8. 10.1074/jbc.M109.020925. 19721136 10.1074/jbc.M109.020925 PMC2785158

[121] Hwang N, Ghanta S, Li Q, Lamattina AM, Murzin E, Lederer JA, et al. Carbon monoxide-induced autophagy enhances human mesenchymal stromal cell function via paracrine actions in murine polymicrobial sepsis. Mol Ther. 2024;32(7):2232–47. 10.1016/j.ymthe.2024.05.018. 38734903 10.1016/j.ymthe.2024.05.018 PMC11286814

[122] Parate D, Kadir ND, Celik C, Lee EH, Hui JHP, Franco-Obregón A, et al. Pulsed electromagnetic fields potentiate the paracrine function of mesenchymal stem cells for cartilage regeneration. Stem Cell Res Ther. 2020;11(1):46. 10.1186/s13287-020-1566-5. 32014064 10.1186/s13287-020-1566-5 PMC6998094

[123] Heo JS. Selenium-Stimulated Exosomes Enhance Wound Healing by Modulating Inflammation and Angiogenesis. International journal of molecular sciences. 2022;23(19). 10.3390/ijms231911543. 10.3390/ijms231911543 PMC9570007 36232844

[124] Liu X, Duan B, Cheng Z, Jia X, Mao L, Fu H, et al. SDF-1/CXCR4 axis modulates bone marrow mesenchymal stem cell apoptosis, migration and cytokine secretion. Protein Cell. 2011;2(10):845–54. 10.1007/s13238-011-1097-z. 22058039 10.1007/s13238-011-1097-z PMC4875294

[125] Ariyanti AD, Zhang J, Marcelina O, Nugrahaningrum DA, Wang G, Kasim V, et al. Salidroside-Pretreated Mesenchymal Stem Cells Enhance Diabetic Wound Healing by Promoting Paracrine Function and Survival of Mesenchymal Stem Cells Under Hyperglycemia. Stem Cells Transl Med. 2019;8(4):404–14. 10.1002/sctm.18-0143. 30624028 10.1002/sctm.18-0143 PMC6431607

[126] Feng Y, Huang W, Wani M, Yu X, Ashraf M. Ischemic preconditioning potentiates the protective effect of stem cells through secretion of exosomes by targeting Mecp2 via miR-22. PLoS ONE. 2014;9(2): e88685. 10.1371/journal.pone.0088685. 24558412 10.1371/journal.pone.0088685 PMC3928277

[127] Sánchez-Sánchez R, Gómez-Ferrer M, Reinal I, Buigues M, Villanueva-Bádenas E, Ontoria-Oviedo I, et al. miR-4732-3p in Extracellular Vesicles From Mesenchymal Stromal Cells Is Cardioprotective During Myocardial Ischemia. Front Cell Dev Biol. 2021;9: 734143. 10.3389/fcell.2021.734143. 34532322 10.3389/fcell.2021.734143 PMC8439391

[128] Santos Nascimento D, Mosqueira D, Sousa LM, Teixeira M, Filipe M, Resende TP, et al. Human umbilical cord tissue-derived mesenchymal stromal cells attenuate remodeling after myocardial infarction by proangiogenic, antiapoptotic, and endogenous cell-activation mechanisms. Stem Cell Res Ther. 2014;5(1):5. 10.1186/scrt394. 24411922 10.1186/scrt394 PMC4055157

[129] Yang Y, Wu Y, Yang D, Neo SH, Kadir ND, Goh D, et al. Secretive derived from hypoxia preconditioned mesenchymal stem cells promote cartilage regeneration and mitigate joint inflammation via extracellular vesicles. Bioact Mater. 2023;27:98–112. 10.1016/j.bioactmat.2023.03.017. 37006826 10.1016/j.bioactmat.2023.03.017 PMC10063382

[130] Kim H, Yu MR, Lee H, Kwon SH, Jeon JS, Han DC, et al. Metformin inhibits chronic kidney disease-induced DNA damage and senescence of mesenchymal stem cells. Aging Cell. 2021;20(2): e13317. 10.1111/acel.13317. 33524231 10.1111/acel.13317 PMC7884040

[131] Sun J, Shen H, Shao L, Teng X, Chen Y, Liu X, et al. HIF-1α overexpression in mesenchymal stem cell-derived exosomes mediates cardioprotection in myocardial infarction by enhanced angiogenesis. Stem Cell Res Ther. 2020;11(1):373. 10.1186/s13287-020-01881-7. 32859268 10.1186/s13287-020-01881-7 PMC7455909

[132] Tang J, Wang J, Yang J, Kong X, Zheng F, Guo L, et al. Mesenchymal stem cells over-expressing SDF-1 promote angiogenesis and improve heart function in experimental myocardial infarction in rats. Eur J Cardiothorac Surg. 2009;36(4):644–50. 10.1016/j.ejcts.2009.04.052. 19524448 10.1016/j.ejcts.2009.04.052

[133] Tang YL, Tang Y, Zhang YC, Qian K, Shen L, Phillips MI. Improved graft mesenchymal stem cell survival in ischemic heart with a hypoxia-regulated heme oxygenase-1 vector. J Am Coll Cardiol. 2005;46(7):1339–50. 10.1016/j.jacc.2005.05.079. 16198853 10.1016/j.jacc.2005.05.079

[134] Wu SZ, Li YL, Huang W, Cai WF, Liang J, Paul C, et al. Paracrine effect of CXCR4-overexpressing mesenchymal stem cells on ischemic heart injury. Cell Biochem Funct. 2017;35(2):113–23. 10.1002/cbf.3254. 28233339 10.1002/cbf.3254 PMC5424893

[135] Yang M, Liao M, Liu R, Zhang Q, Zhang S, He Y, et al. Human umbilical cord mesenchymal stem cell-derived extracellular vesicles loaded with miR-223 ameliorate myocardial infarction through P53/S100A9 axis. Genomics. 2022;114(3): 110319. 10.1016/j.ygeno.2022.110319. 35227836 10.1016/j.ygeno.2022.110319

[136] Chen JJ, Zhou SH. Mesenchymal stem cells overexpressing MiR-126 enhance ischemic angiogenesis via the AKT/ERK-related pathway. Cardiol J. 2011;18(6):675–81. 10.5603/cj.2011.0032. 22113756 10.5603/cj.2011.0032

[137] Huang F, Zhu X, Hu XQ, Fang ZF, Tang L, Lu XL, et al. Mesenchymal stem cells modified with miR-126 release angiogenic factors and activate Notch ligand Delta-like-4, enhancing ischemic angiogenesis and cell survival. Int J Mol Med. 2013;31(2):484–92. 10.3892/ijmm.2012.1200. 23229021 10.3892/ijmm.2012.1200

[138] Li Y, Huang J, Wang J, Xia S, Ran H, Gao L, et al. Human umbilical cord-derived mesenchymal stem cell transplantation supplemented with curcumin improves the outcomes of ischemic stroke via AKT/GSK-3β/β-TrCP/Nrf2 axis. J Neuroinflammation. 2023;20(1):49. 10.1186/s12974-023-02738-5. 36829224 10.1186/s12974-023-02738-5 PMC9951499

[139] Cheng S, Lu Q, Liu Q, Ma Y, Chen J, Lu D, et al. Synergistic effects of repeated transcranial magnetic stimulation and mesenchymal stem cells transplantation on alleviating neuroinflammation and PANoptosis in cerebral ischemia. J Neuroinflammation. 2024;21(1):311. 10.1186/s12974-024-03302-5. 39616364 10.1186/s12974-024-03302-5 PMC11607903

[140] Wang Y, Chen L, Wang L, Pei G, Cheng H, Zhang Q, et al. Pulsed Electromagnetic Fields Combined With Adipose-Derived Stem Cells Protect Ischemic Myocardium by Regulating miR-20a-5p/E2F1/p73 Signaling. Stem cells (Dayton, Ohio). 2023;41(7):724–37. 10.1093/stmcls/sxad037. 37207995 10.1093/stmcls/sxad037

[141] Li Z, Yang B, Yang Z, Xie X, Guo Z, Zhao J, et al. Supramolecular Hydrogel with Ultra-Rapid Cell-Mediated Network Adaptation for Enhancing Cellular Metabolic Energetics and Tissue Regeneration. Advanced materials (Deerfield Beach, Fla). 2024;36(15): e2307176. 10.1002/adma.202307176. 38295393 10.1002/adma.202307176

[142] Chen Y, Shu Z, Qian K, Wang J, Zhu H. Harnessing the Properties of Biomaterial to Enhance the Immunomodulation of Mesenchymal Stem Cells. Tissue Eng Part B Rev. 2019;25(6):492–9. 10.1089/ten.TEB.2019.0131. 31436142 10.1089/ten.TEB.2019.0131

[143] Lee MC, Lee JS, Kim S, Jamaiyar A, Wu W, Gonzalez ML, et al. Synergistic effect of Hypoxic Conditioning and Cell-Tethering Colloidal Gels enhanced Productivity of MSC Paracrine Factors and Accelerated Vessel Regeneration. Advanced materials (Deerfield Beach, Fla). 2024:e2408488. 10.1002/adma.202408488. 10.1002/adma.202408488 PMC11757084 39380372

[144] Li X, Li X, Yang J, Lin J, Zhu Y, Xu X, et al. Living and Injectable Porous Hydrogel Microsphere with Paracrine Activity for Cartilage Regeneration. Small. 2023;19(17): e2207211. 10.1002/smll.202207211. 36651038 10.1002/smll.202207211

[145] Sun L, Wang X, Saredy J, Yuan Z, Yang X, Wang H. Innate-adaptive immunity interplay and redox regulation in immune response. Redox Biol. 2020;37: 101759. 10.1016/j.redox.2020.101759. 33086106 10.1016/j.redox.2020.101759 PMC7575795

[146] Wu X, Reboll MR, Korf-Klingebiel M, Wollert KC. Angiogenesis after acute myocardial infarction. Cardiovasc Res. 2021;117(5):1257–73. 10.1093/cvr/cvaa287. 33063086 10.1093/cvr/cvaa287

[147] van den Akker F, Deddens JC, Doevendans PA, Sluijter JP. Cardiac stem cell therapy to modulate inflammation upon myocardial infarction. Biochim Biophys Acta. 2013;1830(2):2449–58. 10.1016/j.bbagen.2012.08.026. 22975401 10.1016/j.bbagen.2012.08.026

[148] Tang J, Chen Y, Wang C, Xia Y, Yu T, Tang M, et al. The role of mesenchymal stem cells in cancer and prospects for their use in cancer therapeutics. MedComm (2020). 2024;5(8):e663. 10.1002/mco2.663. 10.1002/mco2.663 PMC11283587 39070181

[149] Saigusa R, Winkels H, Ley K. T cell subsets and functions in atherosclerosis. Nat Rev Cardiol. 2020;17(7):387–401. 10.1038/s41569-020-0352-5. 32203286 10.1038/s41569-020-0352-5 PMC7872210

[150] Sage AP, Tsiantoulas D, Binder CJ, Mallat Z. The role of B cells in atherosclerosis. Nat Rev Cardiol. 2019;16(3):180–96. 10.1038/s41569-018-0106-9. 30410107 10.1038/s41569-018-0106-9

[151] McHugh J. T(reg) cell-inducing nanoparticles show promise for treating OA. Nat Rev Rheumatol. 2023;19(2):62. 10.1038/s41584-023-00906-8. 36624262 10.1038/s41584-023-00906-8

[152] Sharabi A, Tsokos GC. T cell metabolism: new insights in systemic lupus erythematosus pathogenesis and therapy. Nat Rev Rheumatol. 2020;16(2):100–12. 10.1038/s41584-019-0356-x. 31949287 10.1038/s41584-019-0356-x

[153] Vilahur G, Juan-Babot O, Peña E, Oñate B, Casaní L, Badimon L. Molecular and cellular mechanisms involved in cardiac remodeling after acute myocardial infarction. J Mol Cell Cardiol. 2011;50(3):522–33. 10.1016/j.yjmcc.2010.12.021. 21219908 10.1016/j.yjmcc.2010.12.021

[154] Arabpour M, Saghazadeh A, Rezaei N. Anti-inflammatory and M2 macrophage polarization-promoting effect of mesenchymal stem cell-derived exosomes. Int Immunopharmacol. 2021;97: 107823. 10.1016/j.intimp.2021.107823. 34102486 10.1016/j.intimp.2021.107823

[155] Copp G, Robb KP, Viswanathan S. Culture-expanded mesenchymal stromal cell therapy: does it work in knee osteoarthritis? A pathway to clinical success. Cell Mol Immunol. 2023;20(6):626–50. 10.1038/s41423-023-01020-1. 37095295 10.1038/s41423-023-01020-1 PMC10229578

[156] Court AC, Vega-Letter AM, Parra-Crisóstomo E, Velarde F, García C, Ortloff A, et al. Mitochondrial transfer balances cell redox, energy and metabolic homeostasis in the osteoarthritic chondrocyte preserving cartilage integrity. Theranostics. 2024;14(17):6471–86. 10.7150/thno.96723. 39479450 10.7150/thno.96723 PMC11519804

[157] Peng S, Sun C, Lai C, Zhang L. Exosomes derived from mesenchymal stem cells rescue cartilage injury in osteoarthritis through Ferroptosis by GOT1/CCR2 expression. Int Immunopharmacol. 2023;122: 110566. 10.1016/j.intimp.2023.110566. 37418985 10.1016/j.intimp.2023.110566

[158] Lagneau N, Tournier P, Nativel F, Maugars Y, Guicheux J, Le Visage C, et al. Harnessing cell-material interactions to control stem cell secretion for osteoarthritis treatment. Biomaterials. 2023;296: 122091. 10.1016/j.biomaterials.2023.122091. 36947892 10.1016/j.biomaterials.2023.122091

[159] Pang L, Jin H, Lu Z, Xie F, Shen H, Li X, et al. Treatment with Mesenchymal Stem Cell-Derived Nanovesicle-Containing Gelatin Methacryloyl Hydrogels Alleviates Osteoarthritis by Modulating Chondrogenesis and Macrophage Polarization. Adv Healthc Mater. 2023;12(17): e2300315. 10.1002/adhm.202300315. 36848378 10.1002/adhm.202300315

[160] Colonna M. TREM1 Blockade: Killing Two Birds with One Stone. Trends Immunol. 2019;40(9):781–3. 10.1016/j.it.2019.07.008. 31439414 10.1016/j.it.2019.07.008

[161] Dabrowska S, Andrzejewska A, Lukomska B, Janowski M. Neuroinflammation as a target for treatment of stroke using mesenchymal stem cells and extracellular vesicles. J Neuroinflammation. 2019;16(1):178. 10.1186/s12974-019-1571-8. 31514749 10.1186/s12974-019-1571-8 PMC6743114

[162] Kwon HS, Koh SH. Neuroinflammation in neurodegenerative disorders: the roles of microglia and astrocytes. Transl Neurodegener. 2020;9(1):42. 10.1186/s40035-020-00221-2. 33239064 10.1186/s40035-020-00221-2 PMC7689983

[163] Stephenson J, Nutma E, van der Valk P, Amor S. Inflammation in CNS neurodegenerative diseases. Immunology. 2018;154(2):204–19. 10.1111/imm.12922. 29513402 10.1111/imm.12922 PMC5980185

[164] Chen Y, Li J, Ma B, Li N, Wang S, Sun Z, et al. MSC-derived exosomes promote recovery from traumatic brain injury via microglia/macrophages in rat. Aging. 2020;12(18):18274–96. 10.18632/aging.103692. 32966240 10.18632/aging.103692 PMC7585083

[165] Liu W, Rong Y, Wang J, Zhou Z, Ge X, Ji C, et al. Exosome-shuttled miR-216a-5p from hypoxic preconditioned mesenchymal stem cells repair traumatic spinal cord injury by shifting microglial M1/M2 polarization. J Neuroinflammation. 2020;17(1):47. 10.1186/s12974-020-1726-7. 32019561 10.1186/s12974-020-1726-7 PMC7001326

[166] Kang J, Guo Y. Human Umbilical Cord Mesenchymal Stem Cells Derived Exosomes Promote Neurological Function Recovery in a Rat Spinal Cord Injury Model. Neurochem Res. 2022;47(6):1532–40. 10.1007/s11064-022-03545-9. 35132478 10.1007/s11064-022-03545-9

[167] Yang Y, Liu Q, Deng S, Shao Q, Peng L, Ling Y, et al. Human umbilical cord derived mesenchymal stem cells overexpressing HO-1 attenuate neural injury and enhance functional recovery by inhibiting inflammation in stroke mice. CNS Neurosci Ther. 2024;30(2): e14412. 10.1111/cns.14412. 37592866 10.1111/cns.14412 PMC10848045

[168] Matusiewicz A, Stróżyńska-Byrska J, Olesińska M. Polyautoimmunity in rheumatological conditions. Int J Rheum Dis. 2019;22(3):386–91. 10.1111/1756-185x.13454. 30548416 10.1111/1756-185X.13454

[169] Porsch F, Binder CJ. Autoimmune diseases and atherosclerotic cardiovascular disease. Nat Rev Cardiol. 2024;21(11):780–807. 10.1038/s41569-024-01045-7. 38937626 10.1038/s41569-024-01045-7

[170] Zaripova LN, Midgley A, Christmas SE, Beresford MW, Pain C, Baildam EM, et al. Mesenchymal Stem Cells in the Pathogenesis and Therapy of Autoimmune and Autoinflammatory Diseases. International journal of molecular sciences. 2023;24(22). 10.3390/ijms242216040. 10.3390/ijms242216040 PMC10671211 38003230

[171] Yasuda K, Takeuchi Y, Hirota K. The pathogenicity of Th17 cells in autoimmune diseases. Semin Immunopathol. 2019;41(3):283–97. 10.1007/s00281-019-00733-8. 30891627 10.1007/s00281-019-00733-8

[172] Park JS, Perl A. Endosome Traffic Modulates Pro-Inflammatory Signal Transduction in CD4(+) T Cells-Implications for the Pathogenesis of Systemic Lupus Erythematosus. International journal of molecular sciences. 2023;24(13). 10.3390/ijms241310749. 10.3390/ijms241310749 PMC10341602 37445926

[173] Ding S, Ren T, Song S, Peng C, Liu C, Wu J, et al. Combined application of mesenchymal stem cells and different glucocorticoid dosing alleviates osteoporosis in SLE murine models. Immun Inflamm Dis. 2024;12(6): e1319. 10.1002/iid3.1319. 38888448 10.1002/iid3.1319 PMC11184931

[174] Ma W, Che J, Chen W, Wang D, Zhang H, Zhao Y. Dexamethasone-Integrated Mesenchymal Stem Cells for Systemic Lupus Erythematosus Treatment via Multiple Immunomodulatory Mechanisms. ACS Nano. 2024;18(20):13249–65. 10.1021/acsnano.4c02420. 38720584 10.1021/acsnano.4c02420

[175] Ma L, Wu H, Cao J, Zhang N, Li Y, Zheng J, et al. Mesenchymal Stem Cell-Based Biomimetic Liposome for Targeted Treatment of Rheumatoid Arthritis. ACS Appl Mater Interfaces. 2024;16(36):47206–15. 10.1021/acsami.4c09080. 39190615 10.1021/acsami.4c09080

[176] Guo J, Lin GS, Bao CY, Hu ZM, Hu MY. Anti-inflammation role for mesenchymal stem cells transplantation in myocardial infarction. Inflammation. 2007;30(3–4):97–104. 10.1007/s10753-007-9025-3. 17497204 10.1007/s10753-007-9025-3

[177] Liu Y, Guan R, Yan J, Zhu Y, Sun S, Qu Y. Mesenchymal Stem Cell-Derived Extracellular Vesicle-Shuttled microRNA-302d-3p Represses Inflammation and Cardiac Remodeling Following Acute Myocardial Infarction. J Cardiovasc Transl Res. 2022;15(4):754–71. 10.1007/s12265-021-10200-1. 35194734 10.1007/s12265-021-10200-1

[178] Zhao J, Li X, Hu J, Chen F, Qiao S, Sun X, et al. Mesenchymal stromal cell-derived exosomes attenuate myocardial ischaemia-reperfusion injury through miR-182-regulated macrophage polarization. Cardiovasc Res. 2019;115(7):1205–16. 10.1093/cvr/cvz040. 30753344 10.1093/cvr/cvz040 PMC6529919

[179] Harrell CR, Fellabaum C, Jovicic N, Djonov V, Arsenijevic N, Volarevic V. Molecular Mechanisms Responsible for Therapeutic Potential of Mesenchymal Stem Cell-Derived Secretome. Cells. 2019;8(5). 10.3390/cells8050467. 10.3390/cells8050467 PMC6562906 31100966

[180] Liu J, Jiang M, Deng S, Lu J, Huang H, Zhang Y, et al. miR-93-5p-Containing Exosomes Treatment Attenuates Acute Myocardial Infarction-Induced Myocardial Damage. Mol Ther Nucleic Acids. 2018;11:103–15. 10.1016/j.omtn.2018.01.010. 29858047 10.1016/j.omtn.2018.01.010 PMC5852413

[181] Wei Z, Qiao S, Zhao J, Liu Y, Li Q, Wei Z, et al. miRNA-181a over-expression in mesenchymal stem cell-derived exosomes influenced inflammatory response after myocardial ischemia-reperfusion injury. Life Sci. 2019;232: 116632. 10.1016/j.lfs.2019.116632. 31278944 10.1016/j.lfs.2019.116632

[182] Cassatella MA, Mosna F, Micheletti A, Lisi V, Tamassia N, Cont C, et al. Toll-like receptor-3-activated human mesenchymal stromal cells significantly prolong the survival and function of neutrophils. Stem Cells. 2011;29(6):1001–11. 10.1002/stem.651. 21563279 10.1002/stem.651

[183] Jiang D, Muschhammer J, Qi Y, Kügler A, de Vries JC, Saffarzadeh M, et al. Suppression of Neutrophil-Mediated Tissue Damage-A Novel Skill of Mesenchymal Stem Cells. Stem Cells. 2016;34(9):2393–406. 10.1002/stem.2417. 27299700 10.1002/stem.2417 PMC5572139

[184] Loh JT, Zhang B, Teo JKH, Lai RC, Choo ABH, Lam KP, et al. Mechanism for the attenuation of neutrophil and complement hyperactivity by MSC exosomes. Cytotherapy. 2022;24(7):711–9. 10.1016/j.jcyt.2021.12.003. 35177337 10.1016/j.jcyt.2021.12.003 PMC8843421

[185] Su VY, Lin CS, Hung SC, Yang KY. Mesenchymal Stem Cell-Conditioned Medium Induces Neutrophil Apoptosis Associated with Inhibition of the NF-κB Pathway in Endotoxin-Induced Acute Lung Injury. Int J Mol Sci. 2019;20(9). 10.3390/ijms20092208. 10.3390/ijms20092208 PMC6540353 31060326

[186] Cho DI, Kim MR, Jeong HY, Jeong HC, Jeong MH, Yoon SH, et al. Mesenchymal stem cells reciprocally regulate the M1/M2 balance in mouse bone marrow-derived macrophages. Exp Mol Med. 2014;46(1): e70. 10.1038/emm.2013.135. 24406319 10.1038/emm.2013.135 PMC3909888

[187] Chen Y, Zuo J, Chen W, Yang Z, Zhang Y, Hua F, et al. The enhanced effect and underlying mechanisms of mesenchymal stem cells with IL-33 overexpression on myocardial infarction. Stem Cell Res Ther. 2019;10(1):295. 10.1186/s13287-019-1392-9. 31547872 10.1186/s13287-019-1392-9 PMC6757387

[188] Wang J, Lee CJ, Deci MB, Jasiewicz N, Verma A, Canty JM, et al. MiR-101a loaded extracellular nanovesicles as bioactive carriers for cardiac repair. Nanomedicine. 2020;27: 102201. 10.1016/j.nano.2020.102201. 32278100 10.1016/j.nano.2020.102201 PMC7647388

[189] Nicholson DW. Caspase structure, proteolytic substrates, and function during apoptotic cell death. Cell Death Differ. 1999;6(11):1028–42. 10.1038/sj.cdd.4400598. 10578171 10.1038/sj.cdd.4400598

[190] Fan TJ, Han LH, Cong RS, Liang J. Caspase family proteases and apoptosis. Acta Biochim Biophys Sin (Shanghai). 2005;37(11):719–27. 10.1111/j.1745-7270.2005.00108.x. 16270150 10.1111/j.1745-7270.2005.00108.x

[191] Shi Y. Mechanisms of caspase activation and inhibition during apoptosis. Mol Cell. 2002;9(3):459–70. 10.1016/s1097-2765(02)00482-3. 11931755 10.1016/s1097-2765(02)00482-3

[192] Mocanu MM, Baxter GF, Yellon DM. Caspase inhibition and limitation of myocardial infarct size: protection against lethal reperfusion injury. Br J Pharmacol. 2000;130(2):197–200. 10.1038/sj.bjp.0703336. 10807653 10.1038/sj.bjp.0703336 PMC1572087

[193] Schwarz K, Simonis G, Yu X, Wiedemann S, Strasser RH. Apoptosis at a distance: remote activation of caspase-3 occurs early after myocardial infarction. Mol Cell Biochem. 2006;281(1–2):45–54. 10.1007/s11010-006-0233-1. 16328956 10.1007/s11010-006-0233-1

[194] Chandrashekhar Y, Sen S, Anway R, Shuros A, Anand I. Long-term caspase inhibition ameliorates apoptosis, reduces myocardial troponin-I cleavage, protects left ventricular function, and attenuates remodeling in rats with myocardial infarction. J Am Coll Cardiol. 2004;43(2):295–301. 10.1016/j.jacc.2003.09.026. 14736452 10.1016/j.jacc.2003.09.026

[195] Liu Q. Lentivirus mediated interference of Caspase-3 expression ameliorates the heart function on rats with acute myocardial infarction. Eur Rev Med Pharmacol Sci. 2014;18(13):1852–8. 25010613

[196] Gao L, Bledsoe G, Yin H, Shen B, Chao L, Chao J. Tissue kallikrein-modified mesenchymal stem cells provide enhanced protection against ischemic cardiac injury after myocardial infarction. Circ J. 2013;77(8):2134–44. 10.1253/circj.cj-12-1585. 23697984 10.1253/circj.cj-12-1585

[197] Ham O, Lee SY, Lee CY, Park JH, Lee J, Seo HH, et al. let-7b suppresses apoptosis and autophagy of human mesenchymal stem cells transplanted into ischemia/reperfusion injured heart 7by targeting caspase-3. Stem Cell Res Ther. 2015;6(1):147. 10.1186/s13287-015-0134-x. 26296645 10.1186/s13287-015-0134-x PMC4546263

[198] Liu X, Li X, Zhu W, Zhang Y, Hong Y, Liang X, et al. Exosomes from mesenchymal stem cells overexpressing MIF enhance myocardial repair. J Cell Physiol. 2020;235(11):8010–22. 10.1002/jcp.29456. 31960418 10.1002/jcp.29456

[199] Xu H, Wang Z, Liu L, Zhang B, Li B. Exosomes derived from adipose tissue, bone marrow, and umbilical cord blood for cardioprotection after myocardial infarction. J Cell Biochem. 2020;121(3):2089–102. 10.1002/jcb.27399. 31736169 10.1002/jcb.27399

[200] Arslan F, Lai RC, Smeets MB, Akeroyd L, Choo A, Aguor EN, et al. Mesenchymal stem cell-derived exosomes increase ATP levels, decrease oxidative stress and activate PI3K/Akt pathway to enhance myocardial viability and prevent adverse remodeling after myocardial ischemia/reperfusion injury. Stem Cell Res. 2013;10(3):301–12. 10.1016/j.scr.2013.01.002. 23399448 10.1016/j.scr.2013.01.002

[201] Wang X, Chen Y, Zhao Z, Meng Q, Yu Y, Sun J, et al. Engineered Exosomes With Ischemic Myocardium-Targeting Peptide for Targeted Therapy in Myocardial Infarction. J Am Heart Assoc. 2018;7(15): e008737. 10.1161/jaha.118.008737. 30371236 10.1161/JAHA.118.008737 PMC6201471

[202] Mao Q, Liang XL, Zhang CL, Pang YH, Lu YX. LncRNA KLF3-AS1 in human mesenchymal stem cell-derived exosomes ameliorates pyroptosis of cardiomyocytes and myocardial infarction through miR-138-5p/Sirt1 axis. Stem Cell Res Ther. 2019;10(1):393. 10.1186/s13287-019-1522-4. 31847890 10.1186/s13287-019-1522-4 PMC6918658

[203] Luther KM, Haar L, McGuinness M, Wang Y, Lynch Iv TL, Phan A, et al. Exosomal miR-21a-5p mediates cardioprotection by mesenchymal stem cells. J Mol Cell Cardiol. 2018;119:125–37. 10.1016/j.yjmcc.2018.04.012. 29698635 10.1016/j.yjmcc.2018.04.012

[204] Cheng H, Chang S, Xu R, Chen L, Song X, Wu J, et al. Hypoxia-challenged MSC-derived exosomes deliver miR-210 to attenuate post-infarction cardiac apoptosis. Stem Cell Res Ther. 2020;11(1):224. 10.1186/s13287-020-01737-0. 32513270 10.1186/s13287-020-01737-0 PMC7278138

[205] Deng S, Zhou X, Ge Z, Song Y, Wang H, Liu X, et al. Exosomes from adipose-derived mesenchymal stem cells ameliorate cardiac damage after myocardial infarction by activating S1P/SK1/S1PR1 signaling and promoting macrophage M2 polarization. Int J Biochem Cell Biol. 2019;114: 105564. 10.1016/j.biocel.2019.105564. 31276786 10.1016/j.biocel.2019.105564

[206] Zhang CS, Shao K, Liu CW, Li CJ, Yu BT. Hypoxic preconditioning BMSCs-exosomes inhibit cardiomyocyte apoptosis after acute myocardial infarction by upregulating microRNA-24. Eur Rev Med Pharmacol Sci. 2019;23(15):6691–9. 10.26355/eurrev_201908_18560. 31378912 10.26355/eurrev_201908_18560

[207] Chen Q, Liu Y, Ding X, Li Q, Qiu F, Wang M, et al. Bone marrow mesenchymal stem cell-secreted exosomes carrying microRNA-125b protect against myocardial ischemia reperfusion injury via targeting SIRT7. Mol Cell Biochem. 2020;465(1–2):103–14. 10.1007/s11010-019-03671-z. 31858380 10.1007/s11010-019-03671-z PMC6955239

[208] Luo Q, Guo D, Liu G, Chen G, Hang M, Jin M. Exosomes from MiR-126-Overexpressing Adscs Are Therapeutic in Relieving Acute Myocardial Ischaemic Injury. Cell Physiol Biochem. 2017;44(6):2105–16. 10.1159/000485949. 29241208 10.1159/000485949

[209] Tan SJO, Floriano JF, Nicastro L, Emanueli C, Catapano F. Novel Applications of Mesenchymal Stem Cell-derived Exosomes for Myocardial Infarction Therapeutics. Biomolecules. 2020;10(5). 10.3390/biom10050707. 10.3390/biom10050707 PMC7277090 32370160

[210] Eble JA, Niland S. The extracellular matrix of blood vessels. Curr Pharm Des. 2009;15(12):1385–400. 10.2174/138161209787846757. 19355976 10.2174/138161209787846757

[211] Carmeliet P, Jain RK. Principles and mechanisms of vessel normalization for cancer and other angiogenic diseases. Nat Rev Drug Discov. 2011;10(6):417–27. 10.1038/nrd3455. 21629292 10.1038/nrd3455

[212] De Smet F, Segura I, De Bock K, Hohensinner PJ, Carmeliet P. Mechanisms of vessel branching: filopodia on endothelial tip cells lead the way. Arterioscler Thromb Vasc Biol. 2009;29(5):639–49. 10.1161/atvbaha.109.185165. 19265031 10.1161/ATVBAHA.109.185165

[213] Potente M, Gerhardt H, Carmeliet P. Basic and therapeutic aspects of angiogenesis. Cell. 2011;146(6):873–87. 10.1016/j.cell.2011.08.039. 21925313 10.1016/j.cell.2011.08.039

[214] Ribatti D, Crivellato E. “Sprouting angiogenesis”, a reappraisal. Dev Biol. 2012;372(2):157–65. 10.1016/j.ydbio.2012.09.018. 23031691 10.1016/j.ydbio.2012.09.018

[215] Bronckaers A, Hilkens P, Martens W, Gervois P, Ratajczak J, Struys T, et al. Mesenchymal stem/stromal cells as a pharmacological and therapeutic approach to accelerate angiogenesis. Pharmacol Ther. 2014;143(2):181–96. 10.1016/j.pharmthera.2014.02.013. 24594234 10.1016/j.pharmthera.2014.02.013

[216] Tang JM, Wang JN, Zhang L, Zheng F, Yang JY, Kong X, et al. VEGF/SDF-1 promotes cardiac stem cell mobilization and myocardial repair in the infarcted heart. Cardiovasc Res. 2011;91(3):402–11. 10.1093/cvr/cvr053. 21345805 10.1093/cvr/cvr053 PMC3139446

[217] Oswald J, Boxberger S, Jørgensen B, Feldmann S, Ehninger G, Bornhäuser M, et al. Mesenchymal stem cells can be differentiated into endothelial cells in vitro. Stem Cells. 2004;22(3):377–84. 10.1634/stemcells.22-3-377. 15153614 10.1634/stemcells.22-3-377

[218] Hao L, Sun H, Wang J, Wang T, Wang M, Zou Z. Mesenchymal stromal cells for cell therapy: besides supporting hematopoiesis. Int J Hematol. 2012;95(1):34–46. 10.1007/s12185-011-0991-8. 22183780 10.1007/s12185-011-0991-8

[219] Kheirandish M, Gavgani SP, Samiee S. The effect of hypoxia preconditioning on the neural and stemness genes expression profiling in human umbilical cord blood mesenchymal stem cells. Transfus Apher Sci. 2017;56(3):392–9. 10.1016/j.transci.2017.03.015. 28428031 10.1016/j.transci.2017.03.015

[220] Kim YS, Noh MY, Cho KA, Kim H, Kwon MS, Kim KS, et al. Hypoxia/Reoxygenation-Preconditioned Human Bone Marrow-Derived Mesenchymal Stromal Cells Rescue Ischemic Rat Cortical Neurons by Enhancing Trophic Factor Release. Mol Neurobiol. 2015;52(1):792–803. 10.1007/s12035-014-8912-5. 25288154 10.1007/s12035-014-8912-5

[221] Hu X, Wei L, Taylor TM, Wei J, Zhou X, Wang JA, et al. Hypoxic preconditioning enhances bone marrow mesenchymal stem cell migration via Kv2.1 channel and FAK activation. Am J Physiol Cell Physiol. 2011;301(2):C362–72. 10.1152/ajpcell.00013.2010. 10.1152/ajpcell.00013.2010 PMC3154562 21562308

[222] Lu F, Zhao X, Wu J, Cui Y, Mao Y, Chen K, et al. MSCs transfected with hepatocyte growth factor or vascular endothelial growth factor improve cardiac function in the infarcted porcine heart by increasing angiogenesis and reducing fibrosis. Int J Cardiol. 2013;167(6):2524–32. 10.1016/j.ijcard.2012.06.052. 22981278 10.1016/j.ijcard.2012.06.052

[223] Sun L, Cui M, Wang Z, Feng X, Mao J, Chen P, et al. Mesenchymal stem cells modified with angiopoietin-1 improve remodeling in a rat model of acute myocardial infarction. Biochem Biophys Res Commun. 2007;357(3):779–84. 10.1016/j.bbrc.2007.04.010. 17445769 10.1016/j.bbrc.2007.04.010

[224] Schmidt A, Ladage D, Schinköthe T, Klausmann U, Ulrichs C, Klinz FJ, et al. Basic fibroblast growth factor controls migration in human mesenchymal stem cells. Stem Cells. 2006;24(7):1750–8. 10.1634/stemcells.2005-0191. 16822883 10.1634/stemcells.2005-0191

[225] Langer HF, Stellos K, Steingen C, Froihofer A, Schönberger T, Krämer B, et al. Platelet derived bFGF mediates vascular integrative mechanisms of mesenchymal stem cells in vitro. J Mol Cell Cardiol. 2009;47(2):315–25. 10.1016/j.yjmcc.2009.03.011. 19328809 10.1016/j.yjmcc.2009.03.011

[226] Miyamoto K, Yazawa T, Mizutani T, Imamichi Y, Kawabe SY, Kanno M, et al. Stem cell differentiation into steroidogenic cell lineages by NR5A family. Mol Cell Endocrinol. 2011;336(1–2):123–6. 10.1016/j.mce.2010.11.031. 21134412 10.1016/j.mce.2010.11.031

[227] Park S, Choi Y, Jung N, Yu Y, Ryu KH, Kim HS, et al. Myogenic differentiation potential of human tonsil-derived mesenchymal stem cells and their potential for use to promote skeletal muscle regeneration. Int J Mol Med. 2016;37(5):1209–20. 10.3892/ijmm.2016.2536. 27035161 10.3892/ijmm.2016.2536 PMC4829138

[228] Yu Y, Huang H, Ye J, Li Y, Xie R, Zeng L, et al. 3D Spheroids Facilitate Differentiation of Human Adipose-Derived Mesenchymal Stem Cells into Hepatocyte-Like Cells via p300-Mediated H3K56 Acetylation. Stem Cells Transl Med. 2024;13(2):151–65. 10.1093/stcltm/szad076. 37936499 10.1093/stcltm/szad076 PMC10872693

[229] Gerami MH, Khorram R, Rasoolzadegan S, Mardpour S, Nakhaei P, Hashemi S, et al. Emerging role of mesenchymal stem/stromal cells (MSCs) and MSCs-derived exosomes in bone- and joint-associated musculoskeletal disorders: a new frontier. Eur J Med Res. 2023;28(1):86. 10.1186/s40001-023-01034-5. 36803566 10.1186/s40001-023-01034-5 PMC9939872

[230] Silva GV, Litovsky S, Assad JA, Sousa AL, Martin BJ, Vela D, et al. Mesenchymal stem cells differentiate into an endothelial phenotype, enhance vascular density, and improve heart function in a canine chronic ischemia model. Circulation. 2005;111(2):150–6. 10.1161/01.Cir.0000151812.86142.45. 15642764 10.1161/01.CIR.0000151812.86142.45

[231] Alviano F, Fossati V, Marchionni C, Arpinati M, Bonsi L, Franchina M, et al. Term Amniotic membrane is a high throughput source for multipotent Mesenchymal Stem Cells with the ability to differentiate into endothelial cells in vitro. BMC Dev Biol. 2007;7:11. 10.1186/1471-213x-7-11. 17313666 10.1186/1471-213X-7-11 PMC1810523

[232] Vittorio O, Jacchetti E, Pacini S, Cecchini M. Endothelial differentiation of mesenchymal stromal cells: when traditional biology meets mechanotransduction. Integr Biol (Camb). 2013;5(2):291–9. 10.1039/c2ib20152f. 23086215 10.1039/c2ib20152f

[233] Shafei AE, Ali MA, Ghanem HG, Shehata AI, Abdelgawad AA, Handal HR, et al. Mesenchymal stem cell therapy: A promising cell-based therapy for treatment of myocardial infarction. J Gene Med. 2017;19(12). 10.1002/jgm.2995. 10.1002/jgm.2995 29044850

[234] Pati S, Khakoo AY, Zhao J, Jimenez F, Gerber MH, Harting M, et al. Human mesenchymal stem cells inhibit vascular permeability by modulating vascular endothelial cadherin/β-catenin signaling. Stem Cells Dev. 2011;20(1):89–101. 10.1089/scd.2010.0013. 20446815 10.1089/scd.2010.0013 PMC3128758

[235] Liu K, Ji K, Guo L, Wu W, Lu H, Shan P, et al. Mesenchymal stem cells rescue injured endothelial cells in an in vitro ischemia-reperfusion model via tunneling nanotube like structure-mediated mitochondrial transfer. Microvasc Res. 2014;92:10–8. 10.1016/j.mvr.2014.01.008. 24486322 10.1016/j.mvr.2014.01.008

[236] Hu X, Ning X, Zhao Q, Zhang Z, Zhang C, Xie M, et al. Islet-1 Mesenchymal Stem Cells-Derived Exosome-Incorporated Angiogenin-1 Hydrogel for Enhanced Acute Myocardial Infarction Therapy. ACS Appl Mater Interfaces. 2022;14(32):36289–303. 10.1021/acsami.2c04686. 35920579 10.1021/acsami.2c04686

[237] Lee JR, Park BW, Kim J, Choo YW, Kim HY, Yoon JK, et al. Nanovesicles derived from iron oxide nanoparticles-incorporated mesenchymal stem cells for cardiac repair. Sci Adv. 2020;6(18):eaaz0952. 10.1126/sciadv.aaz0952. 10.1126/sciadv.aaz0952 PMC7195131 32494669

[238] Kang K, Ma R, Cai W, Huang W, Paul C, Liang J, et al. Exosomes Secreted from CXCR4 Overexpressing Mesenchymal Stem Cells Promote Cardioprotection via Akt Signaling Pathway following Myocardial Infarction. Stem Cells Int. 2015;2015: 659890. 10.1155/2015/659890. 26074976 10.1155/2015/659890 PMC4436515

[239] Huang P, Wang L, Li Q, Tian X, Xu J, Xu J, et al. Atorvastatin enhances the therapeutic efficacy of mesenchymal stem cells-derived exosomes in acute myocardial infarction via up-regulating long non-coding RNA H19. Cardiovasc Res. 2020;116(2):353–67. 10.1093/cvr/cvz139. 31119268 10.1093/cvr/cvz139 PMC8204482

[240] Zhu D, Liu S, Huang K, Wang Z, Hu S, Li J, et al. Intrapericardial Exosome Therapy Dampens Cardiac Injury via Activating Foxo3. Circ Res. 2022;131(10):e135–50. 10.1161/circresaha.122.321384. 36252111 10.1161/CIRCRESAHA.122.321384 PMC9667926

[241] Yao J, Huang K, Zhu D, Chen T, Jiang Y, Zhang J, et al. A Minimally Invasive Exosome Spray Repairs Heart after Myocardial Infarction. ACS Nano. 2021;15(7):11099–111. 10.1021/acsnano.1c00628. 34152126 10.1021/acsnano.1c00628

[242] Roth GA, Mensah GA, Johnson CO, Addolorato G, Ammirati E, Baddour LM, et al. Global Burden of Cardiovascular Diseases and Risk Factors, 1990–2019: Update From the GBD 2019 Study. J Am Coll Cardiol. 2020;76(25):2982–3021. 10.1016/j.jacc.2020.11.010. 33309175 10.1016/j.jacc.2020.11.010 PMC7755038

[243] Shi HT, Huang ZH, Xu TZ, Sun AJ, Ge JB. New diagnostic and therapeutic strategies for myocardial infarction via nanomaterials. EBioMedicine. 2022;78: 103968. 10.1016/j.ebiom.2022.103968. 35367772 10.1016/j.ebiom.2022.103968 PMC8983382

[244] Piamsiri C, Maneechote C, Siri-Angkul N, Chattipakorn SC, Chattipakorn N. Targeting necroptosis as therapeutic potential in chronic myocardial infarction. J Biomed Sci. 2021;28(1):25. 10.1186/s12929-021-00722-w. 33836761 10.1186/s12929-021-00722-w PMC8034148

[245] Hayashi M, Shimizu W, Albert CM. The spectrum of epidemiology underlying sudden cardiac death. Circ Res. 2015;116(12):1887–906. 10.1161/circresaha.116.304521. 26044246 10.1161/CIRCRESAHA.116.304521 PMC4929621

[246] Strauer BE, Brehm M, Zeus T, Köstering M, Hernandez A, Sorg RV, et al. Repair of infarcted myocardium by autologous intracoronary mononuclear bone marrow cell transplantation in humans. Circulation. 2002;106(15):1913–8. 10.1161/01.cir.0000034046.87607.1c. 12370212 10.1161/01.cir.0000034046.87607.1c

[247] Chen SL, Fang WW, Ye F, Liu YH, Qian J, Shan SJ, et al. Effect on left ventricular function of intracoronary transplantation of autologous bone marrow mesenchymal stem cell in patients with acute myocardial infarction. Am J Cardiol. 2004;94(1):92–5. 10.1016/j.amjcard.2004.03.034. 15219514 10.1016/j.amjcard.2004.03.034

[248] Hare JM, Traverse JH, Henry TD, Dib N, Strumpf RK, Schulman SP, et al. A randomized, double-blind, placebo-controlled, dose-escalation study of intravenous adult human mesenchymal stem cells (prochymal) after acute myocardial infarction. J Am Coll Cardiol. 2009;54(24):2277–86. 10.1016/j.jacc.2009.06.055. 19958962 10.1016/j.jacc.2009.06.055 PMC3580848

[249] Kim SH, Cho JH, Lee YH, Lee JH, Kim SS, Kim MY, et al. Improvement in Left Ventricular Function with Intracoronary Mesenchymal Stem Cell Therapy in a Patient with Anterior Wall ST-Segment Elevation Myocardial Infarction. Cardiovasc Drugs Ther. 2018;32(4):329–38. 10.1007/s10557-018-6804-z. 29956042 10.1007/s10557-018-6804-z PMC6133167

[250] Wollert KC, Meyer GP, Müller-Ehmsen J, Tschöpe C, Bonarjee V, Larsen AI, et al. Intracoronary autologous bone marrow cell transfer after myocardial infarction: the BOOST-2 randomised placebo-controlled clinical trial. Eur Heart J. 2017;38(39):2936–43. 10.1093/eurheartj/ehx188. 28431003 10.1093/eurheartj/ehx188

[251] Lunde K, Solheim S, Aakhus S, Arnesen H, Abdelnoor M, Egeland T, et al. Intracoronary injection of mononuclear bone marrow cells in acute myocardial infarction. N Engl J Med. 2006;355(12):1199–209. 10.1056/NEJMoa055706. 16990383 10.1056/NEJMoa055706

[252] Mathiasen AB, Qayyum AA, Jørgensen E, Helqvist S, Kofoed KF, Haack-Sørensen M, et al. Bone marrow-derived mesenchymal stromal cell treatment in patients with ischaemic heart failure: final 4-year follow-up of the MSC-HF trial. Eur J Heart Fail. 2020;22(5):884–92. 10.1002/ejhf.1700. 31863561 10.1002/ejhf.1700

[253] Bolli R, Kahlon A. Time to end the war on cell therapy. Eur J Heart Fail. 2020;22(5):893–7. 10.1002/ejhf.1767. 32100951 10.1002/ejhf.1767

[254] Attar A, Farjoud Kouhanjani M, Hessami K, Vosough M, Kojuri J, Ramzi M, et al. Effect of once versus twice intracoronary injection of allogeneic-derived mesenchymal stromal cells after acute myocardial infarction: BOOSTER-TAHA7 randomized clinical trial. Stem Cell Res Ther. 2023;14(1):264. 10.1186/s13287-023-03495-1. 37740221 10.1186/s13287-023-03495-1 PMC10517503

[255] Zhang R, Yu J, Zhang N, Li W, Wang J, Cai G, et al. Bone marrow mesenchymal stem cells transfer in patients with ST-segment elevation myocardial infarction: single-blind, multicenter, randomized controlled trial. Stem Cell Res Ther. 2021;12(1):33. 10.1186/s13287-020-02096-6. 33413636 10.1186/s13287-020-02096-6 PMC7791674

[256] Lee JW, Lee SH, Youn YJ, Ahn MS, Kim JY, Yoo BS, et al. A randomized, open-label, multicenter trial for the safety and efficacy of adult mesenchymal stem cells after acute myocardial infarction. J Korean Med Sci. 2014;29(1):23–31. 10.3346/jkms.2014.29.1.23. 24431901 10.3346/jkms.2014.29.1.23 PMC3890472

[257] Gao LR, Chen Y, Zhang NK, Yang XL, Liu HL, Wang ZG, et al. Intracoronary infusion of Wharton’s jelly-derived mesenchymal stem cells in acute myocardial infarction: double-blind, randomized controlled trial. BMC Med. 2015;13:162. 10.1186/s12916-015-0399-z. 26162993 10.1186/s12916-015-0399-z PMC4499169

[258] Chullikana A, Majumdar AS, Gottipamula S, Krishnamurthy S, Kumar AS, Prakash VS, et al. Randomized, double-blind, phase I/II study of intravenous allogeneic mesenchymal stromal cells in acute myocardial infarction. Cytotherapy. 2015;17(3):250–61. 10.1016/j.jcyt.2014.10.009. 25484310 10.1016/j.jcyt.2014.10.009

[259] Chen SL, Fang WW, Qian J, Ye F, Liu YH, Shan SJ, et al. Improvement of cardiac function after transplantation of autologous bone marrow mesenchymal stem cells in patients with acute myocardial infarction. Chin Med J (Engl). 2004;117(10):1443–8. 15498362

[260] Mohyeddin-Bonab M, Mohamad-Hassani MR, Alimoghaddam K, Sanatkar M, Gasemi M, Mirkhani H, et al. Autologous in vitro expanded mesenchymal stem cell therapy for human old myocardial infarction. Arch Iran Med. 2007;10(4):467–73. 17903051

[261] Gao LR, Pei XT, Ding QA, Chen Y, Zhang NK, Chen HY, et al. A critical challenge: dosage-related efficacy and acute complication intracoronary injection of autologous bone marrow mesenchymal stem cells in acute myocardial infarction. Int J Cardiol. 2013;168(4):3191–9. 10.1016/j.ijcard.2013.04.112. 23651816 10.1016/j.ijcard.2013.04.112

[262] Heldman AW, DiFede DL, Fishman JE, Zambrano JP, Trachtenberg BH, Karantalis V, et al. Transendocardial mesenchymal stem cells and mononuclear bone marrow cells for ischemic cardiomyopathy: the TAC-HFT randomized trial. JAMA. 2014;311(1):62–73. 10.1001/jama.2013.282909. 24247587 10.1001/jama.2013.282909 PMC4111133

[263] Katritsis DG, Sotiropoulou PA, Karvouni E, Karabinos I, Korovesis S, Perez SA, et al. Transcoronary transplantation of autologous mesenchymal stem cells and endothelial progenitors into infarcted human myocardium. Catheter Cardiovasc Interv. 2005;65(3):321–9. 10.1002/ccd.20406. 15954106 10.1002/ccd.20406

[264] Rodrigo SF, van Ramshorst J, Hoogslag GE, Boden H, Velders MA, Cannegieter SC, et al. Intramyocardial injection of autologous bone marrow-derived ex vivo expanded mesenchymal stem cells in acute myocardial infarction patients is feasible and safe up to 5 years of follow-up. J Cardiovasc Transl Res. 2013;6(5):816–25. 10.1007/s12265-013-9507-7. 23982478 10.1007/s12265-013-9507-7 PMC3790917

[265] Yang Z, Zhang F, Ma W, Chen B, Zhou F, Xu Z, et al. A novel approach to transplanting bone marrow stem cells to repair human myocardial infarction: delivery via a noninfarct-relative artery. Cardiovasc Ther. 2010;28(6):380–5. 10.1111/j.1755-5922.2009.00116.x. 20337639 10.1111/j.1755-5922.2009.00116.x

[266] Wang X, Xi WC, Wang F. The beneficial effects of intracoronary autologous bone marrow stem cell transfer as an adjunct to percutaneous coronary intervention in patients with acute myocardial infarction. Biotechnol Lett. 2014;36(11):2163–8. 10.1007/s10529-014-1589-z. 24975729 10.1007/s10529-014-1589-z

[267] Choudry F, Hamshere S, Saunders N, Veerapen J, Bavnbek K, Knight C, et al. A randomized double-blind control study of early intra-coronary autologous bone marrow cell infusion in acute myocardial infarction: the REGENERATE-AMI clinical trial†. Eur Heart J. 2016;37(3):256–63. 10.1093/eurheartj/ehv493. 26405233 10.1093/eurheartj/ehv493 PMC4712349

[268] Houtgraaf JH, den Dekker WK, van Dalen BM, Springeling T, de Jong R, van Geuns RJ, et al. First experience in humans using adipose tissue-derived regenerative cells in the treatment of patients with ST-segment elevation myocardial infarction. J Am Coll Cardiol. 2012;59(5):539–40. 10.1016/j.jacc.2011.09.065. 22281257 10.1016/j.jacc.2011.09.065

[269] Wöhrle J, von Scheidt F, Schauwecker P, Wiesneth M, Markovic S, Schrezenmeier H, et al. Impact of cell number and microvascular obstruction in patients with bone-marrow derived cell therapy: final results from the randomized, double-blind, placebo controlled intracoronary Stem Cell therapy in patients with Acute Myocardial Infarction (SCAMI) trial. Clin Res Cardiol. 2013;102(10):765–70. 10.1007/s00392-013-0595-9. 23896972 10.1007/s00392-013-0595-9

[270] Schächinger V, Erbs S, Elsässer A, Haberbosch W, Hambrecht R, Hölschermann H, et al. Intracoronary bone marrow-derived progenitor cells in acute myocardial infarction. N Engl J Med. 2006;355(12):1210–21. 10.1056/NEJMoa060186. 16990384 10.1056/NEJMoa060186

[271] Janssens S, Dubois C, Bogaert J, Theunissen K, Deroose C, Desmet W, et al. Autologous bone marrow-derived stem-cell transfer in patients with ST-segment elevation myocardial infarction: double-blind, randomised controlled trial. Lancet. 2006;367(9505):113–21. 10.1016/s0140-6736(05)67861-0. 16413875 10.1016/S0140-6736(05)67861-0

[272] Benedek I, Bucur O, Benedek T. Intracoronary infusion of mononuclear bone marrow-derived stem cells is associated with a lower plaque burden after four years. J Atheroscler Thromb. 2014;21(3):217–29. 10.5551/jat.19745. 24126180 10.5551/jat.19745

[273] Peregud-Pogorzelska M, Przybycień K, Baumert B, Kotowski M, Pius-Sadowska E, Safranow K, et al. The Effect of Intracoronary Infusion of Autologous Bone Marrow-Derived Lineage-Negative Stem/Progenitor Cells on Remodeling of Post-Infarcted Heart in Patient with Acute Myocardial Infarction. Int J Med Sci. 2020;17(8):985–94. 10.7150/ijms.42561. 32410827 10.7150/ijms.42561 PMC7211150

[274] Hopp E, Lunde K, Solheim S, Aakhus S, Arnesen H, Forfang K, et al. Regional myocardial function after intracoronary bone marrow cell injection in reperfused anterior wall infarction - a cardiovascular magnetic resonance tagging study. J Cardiovasc Magn Reson. 2011;13(1):22. 10.1186/1532-429x-13-22. 21414223 10.1186/1532-429X-13-22 PMC3068099

[275] Meyer GP, Wollert KC, Lotz J, Steffens J, Lippolt P, Fichtner S, et al. Intracoronary bone marrow cell transfer after myocardial infarction: eighteen months’ follow-up data from the randomized, controlled BOOST (BOne marrOw transfer to enhance ST-elevation infarct regeneration) trial. Circulation. 2006;113(10):1287–94. 10.1161/circulationaha.105.575118. 16520413 10.1161/CIRCULATIONAHA.105.575118

[276] Lunde K, Solheim S, Aakhus S, Arnesen H, Moum T, Abdelnoor M, et al. Exercise capacity and quality of life after intracoronary injection of autologous mononuclear bone marrow cells in acute myocardial infarction: results from the Autologous Stem cell Transplantation in Acute Myocardial Infarction (ASTAMI) randomized controlled trial. Am Heart J. 2007;154(4):710.e1-8. 10.1016/j.ahj.2007.07.003. 17892996 10.1016/j.ahj.2007.07.003

[277] Golpanian S, El-Khorazaty J, Mendizabal A, DiFede DL, Suncion VY, Karantalis V, et al. Effect of aging on human mesenchymal stem cell therapy in ischemic cardiomyopathy patients. J Am Coll Cardiol. 2015;65(2):125–32. 10.1016/j.jacc.2014.10.040. 25593053 10.1016/j.jacc.2014.10.040 PMC4405121

[278] Malliaras K, Makkar RR, Smith RR, Cheng K, Wu E, Bonow RO, et al. Intracoronary cardiosphere-derived cells after myocardial infarction: evidence of therapeutic regeneration in the final 1-year results of the CADUCEUS trial (CArdiosphere-Derived aUtologous stem CElls to reverse ventricUlar dySfunction). J Am Coll Cardiol. 2014;63(2):110–22. 10.1016/j.jacc.2013.08.724. 24036024 10.1016/j.jacc.2013.08.724 PMC3947063

[279] Florea V, Rieger AC, DiFede DL, El-Khorazaty J, Natsumeda M, Banerjee MN, et al. Dose Comparison Study of Allogeneic Mesenchymal Stem Cells in Patients With Ischemic Cardiomyopathy (The TRIDENT Study). Circ Res. 2017;121(11):1279–90. 10.1161/circresaha.117.311827. 28923793 10.1161/CIRCRESAHA.117.311827 PMC8742223

[280] Suncion VY, Ghersin E, Fishman JE, Zambrano JP, Karantalis V, Mandel N, et al. Does transendocardial injection of mesenchymal stem cells improve myocardial function locally or globally?: An analysis from the Percutaneous Stem Cell Injection Delivery Effects on Neomyogenesis (POSEIDON) randomized trial. Circ Res. 2014;114(8):1292–301. 10.1161/circresaha.114.302854. 24449819 10.1161/CIRCRESAHA.114.302854 PMC4067050

[281] Hare JM, Fishman JE, Gerstenblith G, DiFede Velazquez DL, Zambrano JP, Suncion VY, et al. Comparison of allogeneic vs autologous bone marrow–derived mesenchymal stem cells delivered by transendocardial injection in patients with ischemic cardiomyopathy: the POSEIDON randomized trial. JAMA. 2012;308(22):2369–79. 10.1001/jama.2012.25321. 23117550 10.1001/jama.2012.25321 PMC4762261

[282] Hsiao LC, Lin YN, Shyu WC, Ho M, Lu CR, Chang SS, et al. First-in-human pilot trial of combined intracoronary and intravenous mesenchymal stem cell therapy in acute myocardial infarction. Front Cardiovasc Med. 2022;9: 961920. 10.3389/fcvm.2022.961920. 36017096 10.3389/fcvm.2022.961920 PMC9395611

[283] Li X, Hu YD, Guo Y, Chen Y, Guo DX, Zhou HL, et al. Safety and efficacy of intracoronary human umbilical cord-derived mesenchymal stem cell treatment for very old patients with coronary chronic total occlusion. Curr Pharm Des. 2015;21(11):1426–32. 10.2174/1381612821666141126100636. 25427243 10.2174/1381612821666141126100636

[284] Prat-Vidal C, Rodríguez-Gómez L, Aylagas M, Nieto-Nicolau N, Gastelurrutia P, Agustí E, et al. First-in-human PeriCord cardiac bioimplant: Scalability and GMP manufacturing of an allogeneic engineered tissue graft. EBioMedicine. 2020;54: 102729. 10.1016/j.ebiom.2020.102729. 32304998 10.1016/j.ebiom.2020.102729 PMC7163319

[285] Nauta AJ, Westerhuis G, Kruisselbrink AB, Lurvink EG, Willemze R, Fibbe WE. Donor-derived mesenchymal stem cells are immunogenic in an allogeneic host and stimulate donor graft rejection in a nonmyeloablative setting. Blood. 2006;108(6):2114–20. 10.1182/blood-2005-11-011650. 16690970 10.1182/blood-2005-11-011650 PMC1895546

[286] Harrell CR, Jovicic N, Djonov V, Volarevic V. Therapeutic Use of Mesenchymal Stem Cell-Derived Exosomes: From Basic Science to Clinics. Pharmaceutics. 2020;12(5). 10.3390/pharmaceutics12050474. 10.3390/pharmaceutics12050474 PMC7313713 32456070

[287] J D, Y S, S Z, L C, B L, J Y, et al. Exosomes: key players in cancer and potential therapeutic strategy. Signal transduction and targeted therapy. 2020;5(1):145. 10.1038/s41392-020-00261-0. 10.1038/s41392-020-00261-0 PMC7406508 32759948

[288] Global incidence, prevalence, years lived with disability (YLDs), disability-adjusted life-years (DALYs), and healthy life expectancy (HALE) for 371 diseases and injuries in 204 countries and territories and 811 subnational locations, 1990–2021: a systematic analysis for the Global Burden of Disease Study 2021. Lancet (London, England). 2024;403(10440):2133–61. 10.1016/s0140-6736(24)00757-8. 10.1016/S0140-6736(24)00757-8 PMC11122111 38642570

[289] Gupta A, Vardalakis N, Wagner FB. Neuroprosthetics: from sensorimotor to cognitive disorders. Commun Biol. 2023;6(1):14. 10.1038/s42003-022-04390-w. 36609559 10.1038/s42003-022-04390-w PMC9823108

[290] Namini MS, Daneshimehr F, Beheshtizadeh N, Mansouri V, Ai J, Jahromi HK, et al. Cell-free therapy based on extracellular vesicles: a promising therapeutic strategy for peripheral nerve injury. Stem Cell Res Ther. 2023;14(1):254. 10.1186/s13287-023-03467-5. 37726794 10.1186/s13287-023-03467-5 PMC10510237

[291] Mercuri E, Sumner CJ, Muntoni F, Darras BT, Finkel RS. Spinal muscular atrophy. Nat Rev Dis Primers. 2022;8(1):52. 10.1038/s41572-022-00380-8. 35927425 10.1038/s41572-022-00380-8

[292] El-Badawy A, Amer M, Abdelbaset R, Sherif SN, Abo-Elela M, Ghallab YH, et al. Adipose Stem Cells Display Higher Regenerative Capacities and More Adaptable Electro-Kinetic Properties Compared to Bone Marrow-Derived Mesenchymal Stromal Cells. Sci Rep. 2016;6:37801. 10.1038/srep37801. 27883074 10.1038/srep37801 PMC5121630

[293] Tögel F, Yang Y, Zhang P, Hu Z, Westenfelder C. Bioluminescence imaging to monitor the in vivo distribution of administered mesenchymal stem cells in acute kidney injury. Am J Physiol Renal Physiol. 2008;295(1):F315–21. 10.1152/ajprenal.00098.2008. 18480180 10.1152/ajprenal.00098.2008 PMC4063418

[294] Mohseni R, Hamidieh AA, Shoae-Hassani A, Ghahvechi-Akbari M, Majma A, Mohammadi M, et al. An open-label phase 1 clinical trial of the allogeneic side population adipose-derived mesenchymal stem cells in SMA type 1 patients. Neurol Sci. 2022;43(1):399–410. 10.1007/s10072-021-05291-2. 34032944 10.1007/s10072-021-05291-2

[295] Harris VK, Stark J, Williams A, Roche M, Malin M, Kumar A, et al. Efficacy of intrathecal mesenchymal stem cell-neural progenitor therapy in progressive MS: results from a phase II, randomized, placebo-controlled clinical trial. Stem Cell Res Ther. 2024;15(1):151. 10.1186/s13287-024-03765-6. 38783390 10.1186/s13287-024-03765-6 PMC11119709

[296] Sung Y, Lee SM, Park M, Choi HJ, Kang S, Choi BI, et al. Treatment of traumatic optic neuropathy using human placenta-derived mesenchymal stem cells in Asian patients. Regen Med. 2020;15(10):2163–79. 10.2217/rme-2020-0044. 33315474 10.2217/rme-2020-0044

[297] Tremblay F, Ansari Y, Remaud A, Freedman MS. Neurophysiological outcomes following mesenchymal stem cell therapy in multiple sclerosis. Clin Neurophysiol. 2022;136:69–81. 10.1016/j.clinph.2022.01.125. 35149266 10.1016/j.clinph.2022.01.125

[298] Cotten CM, Fisher K, Malcolm W, Gustafson KE, Cheatham L, Marion A, et al. A Pilot Phase I Trial of Allogeneic Umbilical Cord Tissue-Derived Mesenchymal Stromal Cells in Neonates With Hypoxic-Ischemic Encephalopathy. Stem Cells Transl Med. 2023;12(6):355–64. 10.1093/stcltm/szad027. 37285522 10.1093/stcltm/szad027 PMC10267572

[299] Petrou P, Kassis I, Ginzberg A, Hallimi M, Karussis D. Effects of Mesenchymal Stem Cell Transplantation on Cerebrospinal Fluid Biomarkers in Progressive Multiple Sclerosis. Stem Cells Transl Med. 2022;11(1):55–8. 10.1093/stcltm/szab017. 35641166 10.1093/stcltm/szab017 PMC8895488

[300] Özmert E, Arslan U. Management of toxic optic neuropathy via a combination of Wharton’s jelly-derived mesenchymal stem cells with electromagnetic stimulation. Stem Cell Res Ther. 2021;12(1):518. 10.1186/s13287-021-02577-2. 34579767 10.1186/s13287-021-02577-2 PMC8477499

[301] Siwek T, Jezierska-Woźniak K, Maksymowicz S, Barczewska M, Sowa M, Badowska W, et al. Repeat Administration of Bone Marrow-Derived Mesenchymal Stem Cells for Treatment of Amyotrophic Lateral Sclerosis. Med Sci Monit. 2020;26: e927484. 10.12659/msm.927484. 33301428 10.12659/MSM.927484 PMC7737405

[302] Awidi A, Al Shudifat A, El Adwan N, Alqudah M, Jamali F, Nazer F, et al. Safety and potential efficacy of expanded mesenchymal stromal cells of bone marrow and umbilical cord origins in patients with chronic spinal cord injuries: a phase I/II study. Cytotherapy. 2024;26(8):825–31. 10.1016/j.jcyt.2024.03.480. 38703153 10.1016/j.jcyt.2024.03.480

[303] Albu S, Kumru H, Coll R, Vives J, Vallés M, Benito-Penalva J, et al. Clinical effects of intrathecal administration of expanded Wharton jelly mesenchymal stromal cells in patients with chronic complete spinal cord injury: a randomized controlled study. Cytotherapy. 2021;23(2):146–56. 10.1016/j.jcyt.2020.08.008. 32981857 10.1016/j.jcyt.2020.08.008

[304] Zamani H, Soufizomorrod M, Oraee-Yazdani S, Naviafar D, Akhlaghpasand M, Seddighi A, et al. Safety and feasibility of autologous olfactory ensheathing cell and bone marrow mesenchymal stem cell co-transplantation in chronic human spinal cord injury: a clinical trial. Spinal Cord. 2022;60(1):63–70. 10.1038/s41393-021-00687-5. 34504283 10.1038/s41393-021-00687-5

[305] Li J, Bai X, Guan X, Yuan H, Xu X. Treatment of Optic Canal Decompression Combined with Umbilical Cord Mesenchymal Stem (Stromal) Cells for Indirect Traumatic Optic Neuropathy: A Phase 1 Clinical Trial. Ophthalmic Res. 2021;64(3):398–404. 10.1159/000512469. 33091914 10.1159/000512469

[306] Law ZK, Tan HJ, Chin SP, Wong CY, Wan Yahya WNN, Muda AS, et al. The effects of intravenous infusion of autologous mesenchymal stromal cells in patients with subacute middle cerebral artery infarct: a phase 2 randomized controlled trial on safety, tolerability and efficacy. Cytotherapy. 2021;23(9):833–40. 10.1016/j.jcyt.2021.03.005. 33992536 10.1016/j.jcyt.2021.03.005

[307] Gu J, Huang L, Zhang C, Wang Y, Zhang R, Tu Z, et al. Therapeutic evidence of umbilical cord-derived mesenchymal stem cell transplantation for cerebral palsy: a randomized, controlled trial. Stem Cell Res Ther. 2020;11(1):43. 10.1186/s13287-019-1545-x. 32014055 10.1186/s13287-019-1545-x PMC6998370

[308] Chung JW, Chang WH, Bang OY, Moon GJ, Kim SJ, Kim SK, et al. Efficacy and Safety of Intravenous Mesenchymal Stem Cells for Ischemic Stroke. Neurology. 2021;96(7):e1012–23. 10.1212/wnl.0000000000011440. 33472925 10.1212/WNL.0000000000011440

[309] Zhuo Y, Li WS, Lu W, Li X, Ge LT, Huang Y, et al. TGF-β1 mediates hypoxia-preconditioned olfactory mucosa mesenchymal stem cells improved neural functional recovery in Parkinson’s disease models and patients. Mil Med Res. 2024;11(1):48. 10.1186/s40779-024-00550-7. 39034405 10.1186/s40779-024-00550-7 PMC11265117

[310] Baak LM, Wagenaar N, van der Aa NE, Groenendaal F, Dudink J, Tataranno ML, et al. Feasibility and safety of intranasally administered mesenchymal stromal cells after perinatal arterial ischaemic stroke in the Netherlands (PASSIoN): a first-in-human, open-label intervention study. Lancet Neurol. 2022;21(6):528–36. 10.1016/s1474-4422(22)00117-x. 35568047 10.1016/S1474-4422(22)00117-X

[311] Bydon M, Qu W, Moinuddin FM, Hunt CL, Garlanger KL, Reeves RK, et al. Intrathecal delivery of adipose-derived mesenchymal stem cells in traumatic spinal cord injury: Phase I trial. Nat Commun. 2024;15(1):2201. 10.1038/s41467-024-46259-y. 38561341 10.1038/s41467-024-46259-y PMC10984970

[312] Kim HJ, Cho KR, Jang H, Lee NK, Jung YH, Kim JP, et al. Intracerebroventricular injection of human umbilical cord blood mesenchymal stem cells in patients with Alzheimer’s disease dementia: a phase I clinical trial. Alzheimers Res Ther. 2021;13(1):154. 10.1186/s13195-021-00897-2. 34521461 10.1186/s13195-021-00897-2 PMC8439008

[313] Cudkowicz ME, Lindborg SR, Goyal NA, Miller RG, Burford MJ, Berry JD, et al. A randomized placebo-controlled phase 3 study of mesenchymal stem cells induced to secrete high levels of neurotrophic factors in amyotrophic lateral sclerosis. Muscle Nerve. 2022;65(3):291–302. 10.1002/mus.27472. 34890069 10.1002/mus.27472 PMC9305113

[314] Uccelli A, Laroni A, Ali R, Battaglia MA, Blinkenberg M, Brundin L, et al. Safety, tolerability, and activity of mesenchymal stem cells versus placebo in multiple sclerosis (MESEMS): a phase 2, randomised, double-blind crossover trial. Lancet Neurol. 2021;20(11):917–29. 10.1016/s1474-4422(21)00301-x. 34687636 10.1016/S1474-4422(21)00301-X

[315] Pastor JC, Pastor-Idoate S, López-Paniagua M, Para M, Blazquez F, Murgui E, et al. Intravitreal allogeneic mesenchymal stem cells: a non-randomized phase II clinical trial for acute non-arteritic optic neuropathy. Stem Cell Res Ther. 2023;14(1):261. 10.1186/s13287-023-03500-7. 37735668 10.1186/s13287-023-03500-7 PMC10512539

[316] Lee J, Chang WH, Chung JW, Kim SJ, Kim SK, Lee JS, et al. Efficacy of Intravenous Mesenchymal Stem Cells for Motor Recovery After Ischemic Stroke: A Neuroimaging Study. Stroke. 2022;53(1):20–8. 10.1161/strokeaha.121.034505. 34583525 10.1161/STROKEAHA.121.034505

[317] Jamali F, Aldughmi M, Atiani S, Al-Radaideh A, Dahbour S, Alhattab D, et al. Human Umbilical Cord-Derived Mesenchymal Stem Cells in the Treatment of Multiple Sclerosis Patients: Phase I/II Dose-Finding Clinical Study. Cell Transplant. 2024;33:9636897241233044. 10.1177/09636897241233045. 38450623 10.1177/09636897241233045 PMC10921855

[318] Amanat M, Majmaa A, Zarrabi M, Nouri M, Akbari MG, Moaiedi AR, et al. Clinical and imaging outcomes after intrathecal injection of umbilical cord tissue mesenchymal stem cells in cerebral palsy: a randomized double-blind sham-controlled clinical trial. Stem Cell Res Ther. 2021;12(1):439. 10.1186/s13287-021-02513-4. 34362453 10.1186/s13287-021-02513-4 PMC8343813

[319] Sun JM, Case LE, McLaughlin C, Burgess A, Skergan N, Crane S, et al. Motor function and safety after allogeneic cord blood and cord tissue-derived mesenchymal stromal cells in cerebral palsy: An open-label, randomized trial. Dev Med Child Neurol. 2022;64(12):1477–86. 10.1111/dmcn.15325. 35811372 10.1111/dmcn.15325 PMC9796267

[320] Akhlaghpasand M, Tavanaei R, Hosseinpoor M, Yazdani KO, Soleimani A, Zoshk MY, et al. Safety and potential effects of intrathecal injection of allogeneic human umbilical cord mesenchymal stem cell-derived exosomes in complete subacute spinal cord injury: a first-in-human, single-arm, open-label, phase I clinical trial. Stem Cell Res Ther. 2024;15(1):264. 10.1186/s13287-024-03868-0. 39183334 10.1186/s13287-024-03868-0 PMC11346059

[321] Cohen JA, Lublin FD, Lock C, Pelletier D, Chitnis T, Mehra M, et al. Evaluation of neurotrophic factor secreting mesenchymal stem cells in progressive multiple sclerosis. Mult Scler. 2023;29(1):92–106. 10.1177/13524585221122156. 36113170 10.1177/13524585221122156 PMC9896300

[322] Barczewska M, Maksymowicz S, Zdolińska-Malinowska I, Siwek T, Grudniak M. Umbilical Cord Mesenchymal Stem Cells in Amyotrophic Lateral Sclerosis: an Original Study. Stem Cell Rev Rep. 2020;16(5):922–32. 10.1007/s12015-020-10016-7. 32725316 10.1007/s12015-020-10016-7 PMC7456414

[323] Bang OY, Kim EH, Cho YH, Oh MJ, Chung JW, Chang WH, et al. Circulating Extracellular Vesicles in Stroke Patients Treated With Mesenchymal Stem Cells: A Biomarker Analysis of a Randomized Trial. Stroke. 2022;53(7):2276–86. 10.1161/strokeaha.121.036545. 35341320 10.1161/STROKEAHA.121.036545

[324] de Celis-Ruiz E, Fuentes B, Alonso de Leciñana M, Gutiérrez-Fernández M, Borobia AM, Gutiérrez-Zúñiga R, et al. Final Results of Allogeneic Adipose Tissue-Derived Mesenchymal Stem Cells in Acute Ischemic Stroke (AMASCIS): A Phase II, Randomized, Double-Blind, Placebo-Controlled, Single-Center, Pilot Clinical Trial. Cell Transplant. 2022;31:9636897221083863. 10.1177/09636897221083863. 10.1177/09636897221083863 PMC8943307 35301883

[325] Jaillard A, Hommel M, Moisan A, Zeffiro TA, Favre-Wiki IM, Barbieux-Guillot M, et al. Autologous Mesenchymal Stem Cells Improve Motor Recovery in Subacute Ischemic Stroke: a Randomized Clinical Trial. Transl Stroke Res. 2020;11(5):910–23. 10.1007/s12975-020-00787-z. 32462427 10.1007/s12975-020-00787-z

[326] Petrou P, Kassis I, Yaghmour NE, Ginzberg A, Karussis D. A phase II clinical trial with repeated intrathecal injections of autologous mesenchymal stem cells in patients with amyotrophic lateral sclerosis. Front Biosci (Landmark Ed). 2021;26(10):693–706. 10.52586/4980. 34719198 10.52586/4980

[327] Lu Z, Zhu L, Liu Z, Wu J, Xu Y, Zhang CJ. IV/IT hUC-MSCs Infusion in RRMS and NMO: A 10-Year Follow-Up Study. Front Neurol. 2020;11:967. 10.3389/fneur.2020.00967. 33013641 10.3389/fneur.2020.00967 PMC7506071

[328] Lai B, Wu CH, Wu CY, Luo SF, Lai JH. Ferroptosis and Autoimmune Diseases Front Immunol. 2022;13: 916664. 10.3389/fimmu.2022.916664. 35720308 10.3389/fimmu.2022.916664 PMC9203688

[329] Zx X, Js M, Sg Z. An updated advance of autoantibodies in autoimmune diseases. Autoimmun Rev. 2021;20(2): 102743. 10.1016/j.autrev.2020.102743. 33333232 10.1016/j.autrev.2020.102743

[330] Yang C, Sun J, Tian Y, Li H, Zhang L, Yang J, et al. Immunomodulatory Effect of MSCs and MSCs-Derived Extracellular Vesicles in Systemic Lupus Erythematosus. Front Immunol. 2021;12: 714832. 10.3389/fimmu.2021.714832. 34603289 10.3389/fimmu.2021.714832 PMC8481702

[331] Nakano M, Nagaishi K, Konari N, Saito Y, Chikenji T, Mizue Y, et al. Bone marrow-derived mesenchymal stem cells improve diabetes-induced cognitive impairment by exosome transfer into damaged neurons and astrocytes. Sci Rep. 2016;6:24805. 10.1038/srep24805. 27102354 10.1038/srep24805 PMC4840335

[332] Lee DH, Park KS, Shin HE, Kim SB, Choi H, An SB, et al. Safety and Feasibility of Intradiscal Administration of Matrilin-3-Primed Adipose-Derived Mesenchymal Stromal Cell Spheroids for Chronic Discogenic Low Back Pain: Phase 1 Clinical Trial. Int J Mol Sci. 2023;24(23). 10.3390/ijms242316827. 10.3390/ijms242316827 PMC10706656 38069151

[333] Hernigou P, Auregan JC, Dubory A, Flouzat Lachaniette CH, Rouard H. Ankle osteonecrosis in fifty-one children and adolescent’s leukemia survivors: a prospective randomized study on percutaneous mesenchymal stem cells treatment. Int Orthop. 2021;45(9):2383–93. 10.1007/s00264-021-05051-z. 33893522 10.1007/s00264-021-05051-z

[334] Sreeparvathy R, Belludi SA, Prabhu A. Platelet Rich Fibrin Matrix (PRFM) and Peripheral Blood Mesenchymal Stem Cells (PBMSCs) in the management of intraosseous defects - A randomized clinical trial. J Appl Oral Sci. 2024;32: e20230442. 10.1590/1678-7757-2023-0442. 39109750 10.1590/1678-7757-2023-0442 PMC11321798

[335] Sánchez N, Fierravanti L, Núñez J, Vignoletti F, González-Zamora M, Santamaría S, et al. Periodontal regeneration using a xenogeneic bone substitute seeded with autologous periodontal ligament-derived mesenchymal stem cells: A 12-month quasi-randomized controlled pilot clinical trial. J Clin Periodontol. 2020;47(11):1391–402. 10.1111/jcpe.13368. 32946590 10.1111/jcpe.13368

[336] Infante A, Gener B, Vázquez M, Olivares N, Arrieta A, Grau G, et al. Reiterative infusions of MSCs improve pediatric osteogenesis imperfecta eliciting a pro-osteogenic paracrine response: TERCELOI clinical trial. Clin Transl Med. 2021;11(1): e265. 10.1002/ctm2.265. 33463067 10.1002/ctm2.265 PMC7805402

[337] Hernigou P, Hernigou J, Scarlat M. Mesenchymal stem cell therapy improved outcome of early post-traumatic shoulder osteonecrosis: a prospective randomized clinical study of fifty patients with over ten year follow-up. Int Orthop. 2021;45(10):2643–52. 10.1007/s00264-021-05160-9. 34351460 10.1007/s00264-021-05160-9

[338] Sun H, Zhai H, Han K, Ma H, Tan Y, Li S, et al. Clinical outcomes of autologous adipose-derived mesenchymal stem cell combined with high tibial osteotomy for knee osteoarthritis are correlated with stem cell stemness and senescence. J Transl Med. 2024;22(1):1039. 10.1186/s12967-024-05814-3. 39558365 10.1186/s12967-024-05814-3 PMC11575038

[339] Bolandnazar NS, Raeissadat SA, Haghighatkhah H, Rayegani SM, Oshnari RS, Keshel SH, et al. Safety and efficacy of placental mesenchymal stromal cells-derived extracellular vesicles in knee osteoarthritis: a randomized, triple-blind, placebo-controlled clinical trial. BMC Musculoskelet Disord. 2024;25(1):856. 10.1186/s12891-024-07979-w. 39465400 10.1186/s12891-024-07979-w PMC11514941

[340] Matas J, García C, Poblete D, Vernal R, Ortloff A, Luque-Campos N, et al. A Phase I Dose-Escalation Clinical Trial to Assess the Safety and Efficacy of Umbilical Cord-Derived Mesenchymal Stromal Cells in Knee Osteoarthritis. Stem Cells Transl Med. 2024;13(3):193–203. 10.1093/stcltm/szad088. 38366909 10.1093/stcltm/szad088 PMC10940813

[341] Chen CF, Chen YC, Fu YS, Tsai SW, Wu PK, Chen CM, et al. Safety and Tolerability of Intra-Articular Injection of Adipose-Derived Mesenchymal Stem Cells GXCPC1 in 11 Subjects With Knee Osteoarthritis: A Nonrandomized Pilot Study Without a Control Arm. Cell Transplant. 2024;33:9636897231221882. 10.1177/09636897231221882. 38205679 10.1177/09636897231221882 PMC10785714

[342] Kim KI, Lee MC, Lee JH, Moon YW, Lee WS, Lee HJ, et al. Clinical Efficacy and Safety of the Intra-articular Injection of Autologous Adipose-Derived Mesenchymal Stem Cells for Knee Osteoarthritis: A Phase III, Randomized, Double-Blind. Placebo-Controlled Trial Am J Sports Med. 2023;51(9):2243–53. 10.1177/03635465231179223. 37345256 10.1177/03635465231179223

[343] Sadri B, Hassanzadeh M, Bagherifard A, Mohammadi J, Alikhani M, Moeinabadi-Bidgoli K, et al. Cartilage regeneration and inflammation modulation in knee osteoarthritis following injection of allogeneic adipose-derived mesenchymal stromal cells: a phase II, triple-blinded, placebo controlled, randomized trial. Stem Cell Res Ther. 2023;14(1):162. 10.1186/s13287-023-03359-8. 37316949 10.1186/s13287-023-03359-8 PMC10268462

[344] Kim JH, Kim KI, Yoon WK, Song SJ, Jin W. Intra-articular Injection of Mesenchymal Stem Cells After High Tibial Osteotomy in Osteoarthritic Knee: Two-Year Follow-up of Randomized Control Trial. Stem Cells Transl Med. 2022;11(6):572–85. 10.1093/stcltm/szac023. 35674255 10.1093/stcltm/szac023 PMC9216209

[345] Viganò M, Ragni E, Di Matteo B, Gambaro FM, Perucca Orfei C, Spinelli G, et al. A single step, centrifuge-free method to harvest bone marrow highly concentrated in mesenchymal stem cells: results of a pilot trial. Int Orthop. 2022;46(2):391–400. 10.1007/s00264-021-05243-7. 34727209 10.1007/s00264-021-05243-7

[346] Lamo-Espinosa JM, Blanco JF, Sánchez M, Moreno V, Granero-Moltó F, Sánchez-Guijo F, et al. Phase II multicenter randomized controlled clinical trial on the efficacy of intra-articular injection of autologous bone marrow mesenchymal stem cells with platelet rich plasma for the treatment of knee osteoarthritis. J Transl Med. 2020;18(1):356. 10.1186/s12967-020-02530-6. 32948200 10.1186/s12967-020-02530-6 PMC7501623

[347] Bastos R, Mathias M, Andrade R, Amaral R, Schott V, Balduino A, et al. Intra-articular injection of culture-expanded mesenchymal stem cells with or without addition of platelet-rich plasma is effective in decreasing pain and symptoms in knee osteoarthritis: a controlled, double-blind clinical trial. Knee Surg Sports Traumatol Arthrosc. 2020;28(6):1989–99. 10.1007/s00167-019-05732-8. 31587091 10.1007/s00167-019-05732-8

[348] Vij R, Stebbings KA, Kim H, Park H, Chang D. Safety and efficacy of autologous, adipose-derived mesenchymal stem cells in patients with rheumatoid arthritis: a phase I/IIa, open-label, non-randomized pilot trial. Stem Cell Res Ther. 2022;13(1):88. 10.1186/s13287-022-02763-w. 35241141 10.1186/s13287-022-02763-w PMC8896321

[349] Qi T, Gao H, Dang Y, Huang S, Peng M. Cervus and cucumis peptides combined umbilical cord mesenchymal stem cells therapy for rheumatoid arthritis. Medicine (Baltimore). 2020;99(28): e21222. 10.1097/md.0000000000021222. 32664175 10.1097/MD.0000000000021222 PMC7360298

[350] Kamen DL, Wallace C, Li Z, Wyatt M, Paulos C, Wei C, et al. Safety, immunological effects and clinical response in a phase I trial of umbilical cord mesenchymal stromal cells in patients with treatment refractory SLE. Lupus Sci Med. 2022;9(1). 10.1136/lupus-2022-000704. 10.1136/lupus-2022-000704 PMC9277402 35820718

[351] Fernández O, Izquierdo G, Fernández V, Leyva L, Reyes V, Guerrero M, et al. Adipose-derived mesenchymal stem cells (AdMSC) for the treatment of secondary-progressive multiple sclerosis: A triple blinded, placebo controlled, randomized phase I/II safety and feasibility study. PLoS ONE. 2018;13(5): e0195891. 10.1371/journal.pone.0195891. 29768414 10.1371/journal.pone.0195891 PMC5955528

[352] Chun S, Choi CB, Kim MS, Nam JY, Lee TY, Lee YT, et al. Safety and tolerability of bone marrow-derived mesenchymal stem cells in lupus animal models and a phase I clinical trial in humans. Lupus. 2022;31(10):1245–53. 10.1177/09612033221111957. 35802867 10.1177/09612033221111957

[353] Alwabli Y, Almatroudi MA, Alharbi MA, Alharbi MY, Alreshood S, Althwiny FA. Work-Related Musculoskeletal Disorders Among Medical Practitioners in the Hospitals of Al’Qassim Region, Saudi Arabia. Cureus. 2020;12(5): e8382. 10.7759/cureus.8382. 32637265 10.7759/cureus.8382 PMC7331922

[354] Chenna D, Pentapati KC, Kumar M, Madi M, Siddiq H. Prevalence of musculoskeletal disorders among dental healthcare providers: A systematic review and meta-analysis. F1000Res. 2022;11:1062. 10.12688/f1000research.124904.2. 10.12688/f1000research.124904.1 PMC9709350 36505095

[355] Kodama J, Wilkinson KJ, Otsuru S. MSC-EV therapy for bone/cartilage diseases. Bone Rep. 2022;17: 101636. 10.1016/j.bonr.2022.101636. 36389627 10.1016/j.bonr.2022.101636 PMC9663871

[356] Saeedi P, Halabian R, Imani Fooladi AA. A revealing review of mesenchymal stem cells therapy, clinical perspectives and Modification strategies. Stem Cell Investig. 2019;6:34. 10.21037/sci.2019.08.11. 31620481 10.21037/sci.2019.08.11 PMC6789202

[357] Zhang X, He J, Wang W. Progress in the use of mesenchymal stromal cells for osteoarthritis treatment. Cytotherapy. 2021;23(6):459–70. 10.1016/j.jcyt.2021.01.008. 33736933 10.1016/j.jcyt.2021.01.008

[358] Gilbertie JM, Long JM, Schubert AG, Berglund AK, Schaer TP, Schnabel LV. Pooled Platelet-Rich Plasma Lysate Therapy Increases Synoviocyte Proliferation and Hyaluronic Acid Production While Protecting Chondrocytes From Synoviocyte-Derived Inflammatory Mediators. Front Vet Sci. 2018;5:150. 10.3389/fvets.2018.00150. 30023361 10.3389/fvets.2018.00150 PMC6039577

[359] Chen Y, Xu Y, Chi Y, Sun T, Gao Y, Dou X, et al. Efficacy and safety of human umbilical cord-derived mesenchymal stem cells in the treatment of refractory immune thrombocytopenia: a prospective, single arm, phase I trial. Signal Transduct Target Ther. 2024;9(1):102. 10.1038/s41392-024-01793-5. 38653983 10.1038/s41392-024-01793-5 PMC11039759

[360] Carlsson PO, Espes D, Sisay S, Davies LC, Smith CIE, Svahn MG. Umbilical cord-derived mesenchymal stromal cells preserve endogenous insulin production in type 1 diabetes: a Phase I/II randomised double-blind placebo-controlled trial. Diabetologia. 2023;66(8):1431–41. 10.1007/s00125-023-05934-3. 37221247 10.1007/s00125-023-05934-3 PMC10317874

[361] Harris VK, Stark JW, Yang S, Zanker S, Tuddenham J, Sadiq SA. Mesenchymal stem cell-derived neural progenitors in progressive MS: Two-year follow-up of a phase I study. Neurol Neuroimmunol Neuroinflamm. 2021;8(1). 10.1212/nxi.0000000000000928. 10.1212/NXI.0000000000000928 PMC7738177 33277427

[362] Wu Z, Xu X, Cai J, Chen J, Huang L, Wu W, et al. Prevention of chronic diabetic complications in type 1 diabetes by co-transplantation of umbilical cord mesenchymal stromal cells and autologous bone marrow: a pilot randomized controlled open-label clinical study with 8-year follow-up. Cytotherapy. 2022;24(4):421–7. 10.1016/j.jcyt.2021.09.015. 35086778 10.1016/j.jcyt.2021.09.015

[363] Ma C, Feng Y, Yang L, Wang S, Sun X, Tai S, et al. In vitro Immunomodulatory Effects of Human Umbilical Cord-Derived Mesenchymal Stem Cells on Peripheral Blood Cells from Warm Autoimmune Hemolytic Anemia Patients. Acta Haematol. 2022;145(1):63–71. 10.1159/000506759. 34284381 10.1159/000506759

[364] Lu J, Shen SM, Ling Q, Wang B, Li LR, Zhang W, et al. One repeated transplantation of allogeneic umbilical cord mesenchymal stromal cells in type 1 diabetes: an open parallel controlled clinical study. Stem Cell Res Ther. 2021;12(1):340. 10.1186/s13287-021-02417-3. 34112266 10.1186/s13287-021-02417-3 PMC8194026

[365] Wang L, Huang S, Li S, Li M, Shi J, Bai W, et al. Efficacy and Safety of Umbilical Cord Mesenchymal Stem Cell Therapy for Rheumatoid Arthritis Patients: A Prospective Phase I/II Study. Drug Des Devel Ther. 2019;13:4331–40. 10.2147/dddt.S225613. 31908418 10.2147/DDDT.S225613 PMC6930836

[366] Swart JF, de Roock S, Nievelstein RAJ, Slaper-Cortenbach ICM, Boelens JJ, Wulffraat NM. Bone-marrow derived mesenchymal stromal cells infusion in therapy refractory juvenile idiopathic arthritis patients. Rheumatology (Oxford). 2019;58(10):1812–7. 10.1093/rheumatology/kez157. 31070229 10.1093/rheumatology/kez157 PMC6758577

[367] Ghoryani M, Shariati-Sarabi Z, Tavakkol-Afshari J, Ghasemi A, Poursamimi J, Mohammadi M. Amelioration of clinical symptoms of patients with refractory rheumatoid arthritis following treatment with autologous bone marrow-derived mesenchymal stem cells: A successful clinical trial in Iran. Biomed Pharmacother. 2019;109:1834–40. 10.1016/j.biopha.2018.11.056. 30551438 10.1016/j.biopha.2018.11.056

[368] Liang J, Zhang H, Kong W, Deng W, Wang D, Feng X, et al. Safety analysis in patients with autoimmune disease receiving allogeneic mesenchymal stem cells infusion: a long-term retrospective study. Stem Cell Res Ther. 2018;9(1):312. 10.1186/s13287-018-1053-4. 30428931 10.1186/s13287-018-1053-4 PMC6236873

[369] Park EH, Lim HS, Lee S, Roh K, Seo KW, Kang KS, et al. Intravenous Infusion of Umbilical Cord Blood-Derived Mesenchymal Stem Cells in Rheumatoid Arthritis: A Phase Ia Clinical Trial. Stem Cells Transl Med. 2018;7(9):636–42. 10.1002/sctm.18-0031. 30112846 10.1002/sctm.18-0031 PMC6127229

[370] Ellison-Hughes GM, Colley L, O’Brien KA, Roberts KA, Agbaedeng TA, Ross MD. The Role of MSC Therapy in Attenuating the Damaging Effects of the Cytokine Storm Induced by COVID-19 on the Heart and Cardiovascular System. Front Cardiovasc Med. 2020;7: 602183. 10.3389/fcvm.2020.602183. 33363221 10.3389/fcvm.2020.602183 PMC7756089

[371] Walter J, Ware LB, Matthay MA. Mesenchymal stem cells: mechanisms of potential therapeutic benefit in ARDS and sepsis. Lancet Respir Med. 2014;2(12):1016–26. 10.1016/s2213-2600(14)70217-6. 25465643 10.1016/S2213-2600(14)70217-6

[372] Vlahos R, Bozinovski S. Role of alveolar macrophages in chronic obstructive pulmonary disease. Front Immunol. 2014;5:435. 10.3389/fimmu.2014.00435. 25309536 10.3389/fimmu.2014.00435 PMC4160089

[373] Kharaziha P, Hellström PM, Noorinayer B, Farzaneh F, Aghajani K, Jafari F, et al. Improvement of liver function in liver cirrhosis patients after autologous mesenchymal stem cell injection: a phase I-II clinical trial. Eur J Gastroenterol Hepatol. 2009;21(10):1199–205. 10.1097/MEG.0b013e32832a1f6c. 19455046 10.1097/MEG.0b013e32832a1f6c

[374] Anders HJ, Huber TB, Isermann B, Schiffer M. CKD in diabetes: diabetic kidney disease versus nondiabetic kidney disease. Nat Rev Nephrol. 2018;14(6):361–77. 10.1038/s41581-018-0001-y. 29654297 10.1038/s41581-018-0001-y

[375] Barrera-Chimal J, Jaisser F. Pathophysiologic mechanisms in diabetic kidney disease: A focus on current and future therapeutic targets. Diabetes Obes Metab. 2020;22(Suppl 1):16–31. 10.1111/dom.13969. 32267077 10.1111/dom.13969

[376] Khalilpourfarshbafi M, Hajiaghaalipour F, Selvarajan KK, Adam A. Mesenchymal Stem Cell-Based Therapies against Podocyte Damage in Diabetic Nephropathy. Tissue Eng Regen Med. 2017;14(3):201–10. 10.1007/s13770-017-0026-5. 30603477 10.1007/s13770-017-0026-5 PMC6171601

[377] Jin J, Qian F, Zheng D, He W, Gong J, He Q. Mesenchymal Stem Cells Attenuate Renal Fibrosis via Exosomes-Mediated Delivery of microRNA Let-7i-5p Antagomir. Int J Nanomedicine. 2021;16:3565–78. 10.2147/ijn.S299969. 34079249 10.2147/IJN.S299969 PMC8164705

[378] Yang Y, Wang J, Zhang Y, Hu X, Li L, Chen P. Exosomes derived from mesenchymal stem cells ameliorate renal fibrosis via delivery of miR-186-5p. Hum Cell. 2022;35(1):83–97. 10.1007/s13577-021-00617-w. 34585365 10.1007/s13577-021-00617-w

[379] Vivarelli M, Colucci M, Algeri M, Zotta F, Emma F, L'Erario I, et al. A phase I study of autologous mesenchymal stromal cells for severe steroid-dependent nephrotic syndrome. JCI Insight. 2023;8(18). 10.1172/jci.insight.169424. 10.1172/jci.insight.169424 PMC10561718 37561590

[380] Perico N, Remuzzi G, Griffin MD, Cockwell P, Maxwell AP, Casiraghi F, et al. Safety and Preliminary Efficacy of Mesenchymal Stromal Cell (ORBCEL-M) Therapy in Diabetic Kidney Disease: A Randomized Clinical Trial (NEPHSTROM). J Am Soc Nephrol. 2023;34(10):1733–51. 10.1681/asn.0000000000000189. 37560967 10.1681/ASN.0000000000000189 PMC10561817

[381] Swaminathan M, Kopyt N, Atta MG, Radhakrishnan J, Umanath K, Nguyen S, et al. Pharmacological effects of ex vivo mesenchymal stem cell immunotherapy in patients with acute kidney injury and underlying systemic inflammation. Stem Cells Transl Med. 2021;10(12):1588–601. 10.1002/sctm.21-0043. 34581517 10.1002/sctm.21-0043 PMC8641088

[382] Abumoawad A, Saad A, Ferguson CM, Eirin A, Herrmann SM, Hickson LJ, et al. In a Phase 1a escalating clinical trial, autologous mesenchymal stem cell infusion for renovascular disease increases blood flow and the glomerular filtration rate while reducing inflammatory biomarkers and blood pressure. Kidney Int. 2020;97(4):793–804. 10.1016/j.kint.2019.11.022. 32093917 10.1016/j.kint.2019.11.022 PMC7284953

[383] J L, H Z, C Z, D W, X M, S Z, et al. Effects of allogeneic mesenchymal stem cell transplantation in the treatment of liver cirrhosis caused by autoimmune diseases. International journal of rheumatic diseases. 2017;20(9):1219–26. 10.1111/1756-185x.13015. 10.1111/1756-185X.13015 28217916

[384] Chu M, Wang H, Bian L, Huang J, Wu D, Zhang R, et al. Nebulization Therapy with Umbilical Cord Mesenchymal Stem Cell-Derived Exosomes for COVID-19 Pneumonia. Stem Cell Rev Rep. 2022;18(6):2152–63. 10.1007/s12015-022-10398-w. 35665467 10.1007/s12015-022-10398-w PMC9166932

[385] Weiss DJ, Segal K, Casaburi R, Hayes J, Tashkin D. Effect of mesenchymal stromal cell infusions on lung function in COPD patients with high CRP levels. Respir Res. 2021;22(1):142. 10.1186/s12931-021-01734-8. 33964910 10.1186/s12931-021-01734-8 PMC8106850

[386] Zheng G, Huang L, Tong H, Shu Q, Hu Y, Ge M, et al. Treatment of acute respiratory distress syndrome with allogeneic adipose-derived mesenchymal stem cells: a randomized, placebo-controlled pilot study. Respir Res. 2014;15(1):39. 10.1186/1465-9921-15-39. 24708472 10.1186/1465-9921-15-39 PMC3994204

[387] Harrell CR, Volarevic A, Djonov V, Volarevic V. Mesenchymal Stem-Cell-Derived Exosomes as Novel Drug Carriers in Anti-Cancer Treatment: A Myth or Reality? Cells. 2025;14(3). 10.3390/cells14030202. 10.3390/cells14030202 PMC11817634 39936993

